# Rigidified Derivative of the Non-macrocyclic Ligand H_4_OCTAPA for Stable Lanthanide(III) Complexation

**DOI:** 10.1021/acs.inorgchem.2c00501

**Published:** 2022-03-11

**Authors:** Fátima Lucio-Martínez, Zoltán Garda, Balázs Váradi, Ferenc Krisztián Kálmán, David Esteban-Gómez, Éva Tóth, Gyula Tircsó, Carlos Platas-Iglesias

**Affiliations:** † Centro de Investigacións Científicas Avanzadas (CICA) and Departamento de Química, Facultade de Ciencias, Universidade da Coruña, 15071 A Coruña, Galicia, Spain; ‡ Department of Physical Chemistry, University of Debrecen, Egyetem tér 1, H-4010 Debrecen, Hungary; § Doctoral School of Chemistry, University of Debrecen, Egyetem tér 1, H-4010 Debrecen, Hungary; ∥ Centre de Biophysique Moléculaire, CNRS UPR 4301, Université d’Orléans, rue Charles Sadron, 45071 Orléans, Cedex 2, France

## Abstract

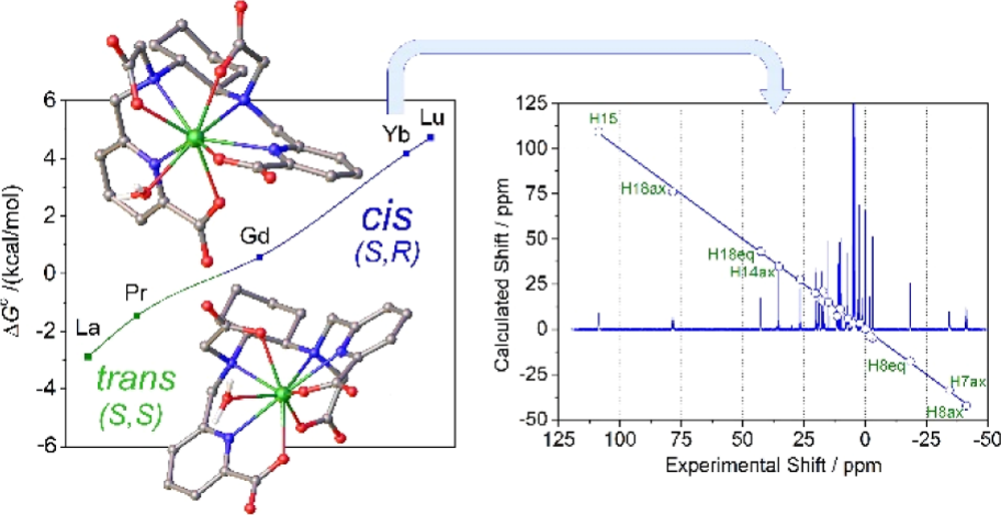

The stability constants of lanthanide complexes with the potentially octadentate ligand *CHX*OCTAPA^4–^, which contains a rigid 1,2-diaminocyclohexane scaffold functionalized with two acetate and two picolinate pendant arms, reveal the formation of stable complexes [log *K*_LaL_ = 17.82(1) and log *K*_YbL_ = 19.65(1)]. Luminescence studies on the Eu^3+^ and Tb^3+^ analogues evidenced rather high emission quantum yields of 3.4 and 11%, respectively. The emission lifetimes recorded in H_2_O and D_2_O solutions indicate the presence of a water molecule coordinated to the metal ion. ^1^H nuclear magnetic relaxation dispersion profiles and ^17^O NMR chemical shift and relaxation measurements point to a rather low water exchange rate of the coordinated water molecule (*k*_ex_^298^ = 1.58 × 10^6^ s^–1^) and relatively high relaxivities of 5.6 and 4.5 mM^–1^ s^–1^ at 20 MHz and 25 and 37 °C, respectively. Density functional theory calculations and analysis of the paramagnetic shifts induced by Yb^3+^ indicate that the complexes adopt an unprecedented cis geometry with the two picolinate groups situated on the same side of the coordination sphere. Dissociation kinetics experiments were conducted by investigating the exchange reactions of LuL occurring with Cu^2+^. The results confirmed the beneficial effect of the rigid cyclohexyl group on the inertness of the Lu^3+^ complex. Complex dissociation occurs following proton- and metal-assisted pathways. The latter is relatively efficient at neutral pH, thanks to the formation of a heterodinuclear hydroxo complex.

## Abstract 2

A non-macrocyclic ligand containing a rigid cyclohexyl spacer forms thermodynamically stable complexes with the lanthanide(III) ions in aqueous solution. The complexes also show remarkable kinetic inertness, though a structural change facilitates dissociation through the metal-assisted mechanism for the small lanthanides. The Gd(III) complex displays a relatively high relaxivity due to the presence of a water molecule coordinated to the metal ion, while the Eu(III) and Tb(III) analogues display strong metal-centered luminescence.

## Introduction

Stable complexation of lanthanide ions (Ln^3+^) in aqueous solution is a coordination chemistry problem that has received much attention in the last 3 decades. This interest is related to a great extent to the important medical and biomedical properties of some lanthanide complexes, which include (1) the use of Gd^3+^ complexes as contrast agents in magnetic resonance imaging (MRI),^1−5^ (2) the potential of luminescent Ln^3+^ complexes, particularly Eu^3+^ and Tb^3+^, in optical imaging and bioanalytical applications,^6−8^ and (3) the interesting properties of radioisotopes in the lanthanide series (i.e., ^177^Lu) for radiopharmaceutical applications.^9,10^ All these applications require stable complexation of metal ions and slow dissociation kinetics to avoid undesirable effects (toxicity issues).^11,12^ Furthermore, the application of Ln^3+^ complexes as radiopharmaceuticals requires a fast complexation of the radioisotope under mild conditions.^13^ Chelates for the preparation of efficient luminescent complexes must contain chromophore units suitable for indirect excitation of the relevant Ln^3+^ excited state, while protecting the metal ion from the vibrational quenching associated to the coordination of water molecules.^14^

**Chart 1:**
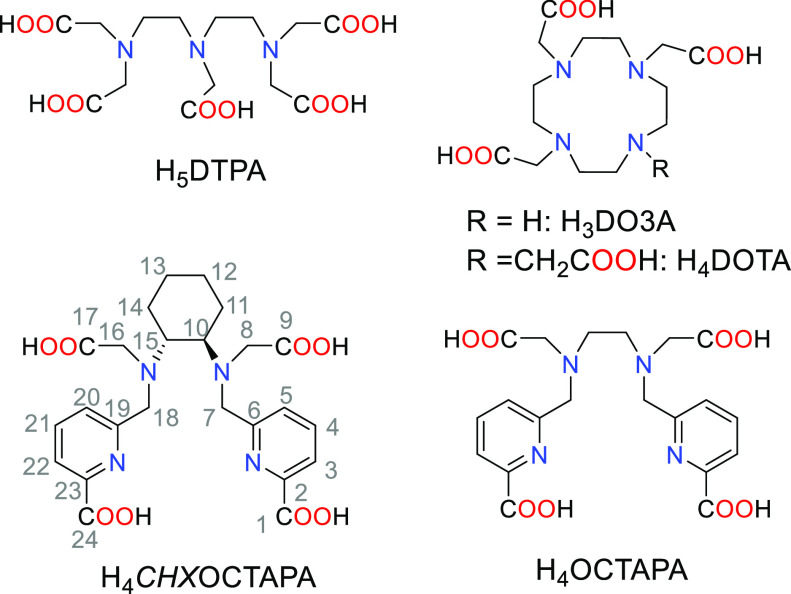
Ligands Discussed in the Present Work

The chelates used for stable Ln^3+^ complexes are often either macrocyclic or non-macrocyclic systems containing hard carboxylate or phosphonate donor groups whose denticity ranges from 7 to 10.^15−17^ Ligands with lower denticity like EDTA result in complexes endowed with low stability,^18^ while octa- or nonadentate ligands generally present favorable complexation properties.^19,20^ Macrocyclic ligands often form complexes with superior thermodynamic stability and exceptional kinetic inertness,^21^ but in some cases lead to very slow complexation kinetics.^21−24^ On the other hand, non-macrocyclic ligands such as DTPA^5–^ (Chart 1) and DTPA bisamides often present faster dissociation kinetics, which is problematic for medical applications.^11,25^ Gd^3+^ complexes with DTPA bisamides were considered to have superior kinetic inertness than the DTPA^5–^ analogue. However, more recent studies demonstrated that different anions present in vivo catalyze the dissociation of Gd^3+^ complexes with DTPA bisamides.^11^

In 2004, we reported the potentially octadentate ligand H_4_OCTAPA (Chart 1), whose Gd^3+^ complex was originally designed as a potential MRI contrast agent candidate.^26^ This study demonstrated the presence of a water molecule in the inner coordination sphere. Subsequent investigations performed by Mazzanti,^27,28^ Orvig,^29,30^ and our own group^31^ pointed to a high thermodynamic stability of the lanthanide complexes, which, however, exhibit fast dissociation kinetics. Orvig and co-workers showed that OCTAPA presents very promising properties for the development of ^111^In, ^90^Y, and ^177^Lu radiopharmaceuticals.^32,33^ Bifunctional derivatives of H_4_OCTAPA were also reported and successfully tested in vivo upon radiolabeling with these radioisotopes.^34−36^ The rigidified ligand *CHX*OCTAPA^4–^ (also known as H_4_CDDADPA^4–^) was reported almost simultaneously by the group of Orvig and us.^37,38^ The corresponding Gd^3+^ complex is remarkably inert with respect to dissociation, with dissociation rate constants comparable to those of macrocyclic complexes such as [Gd(DO3A)].

In this paper, we present a detailed characterization of the Ln^3+^ complexes of *CHX*OCTAPA using a wide range of experimental and computational techniques. A multinuclear (^1^H and ^13^C) NMR study and density functional theory (DFT) calculations were used to establish the structure of the complexes in solution, including the analysis of the paramagnetic Yb^3+^-induced ^1^H NMR shifts. These studies revealed unexpected features of the structure in solution of these complexes. We also present a full characterization of the relaxometric properties of the Gd^3+^ complex involving ^1^H nuclear magnetic relaxation dispersion (NMRD) studies and ^17^O NMR chemical shifts and relaxation rates. A detailed analysis of the photophysical properties of the Eu^3+^ and Tb^3+^ complexes, including quantum yield determination, is reported. Finally, we also determined the stability of some of the complexes across the lanthanide series and assessed their kinetics of dissociation. The stabilities of the complexes formed with divalent metal ions of biological relevance are also reported.

## Results and Discussion

### Stability of the Ln^3+^ Complexes

Stability constant determination requires measuring the protonation constants of the ligand using the same electrolyte background. The protonation constants of *CHX*OCTAPA^4–^ in 0.15 M NaCl reported previously by Orvig^37^ and us^38^ were in good agreement, though slight discrepancies can be noticed for log *K*_5_^H^ and log *K*_6_^H^ (Table S1, Supporting Information). These protonation processes take place in the pH range where complex dissociation occurs, and thus the accurate determination of their values is critical for determining stability constants. We therefore performed new potentiometric titrations using a higher ligand concentration (4.38 mM) in the pH range 1.65–11.95, which allows for a more accurate estimation of protonation constants (Figure S30, Supporting Information). These experiments yielded log *K*_5_^H^ = 1.59(1) and log *K*_6_^H^ = 0.61(4).

**Figure 1:**
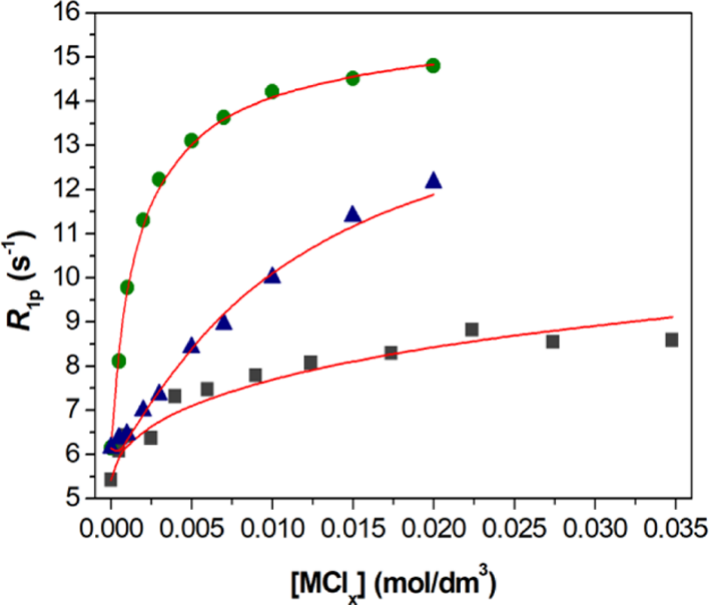
Relaxometric titrations (25 °C, 0.15 M NaCl) of the [Gd(CHXOCTAPA)]^−^ complex with LaCl_3_ (squares, *c*_Lig_ = *c*_Gd3+_ = 1.001 mM at pH = 4.69), YbCl_3_ (circles, *c*_Lig_ = *c*_Gd3+_ = 1.113 mM at pH = 4.79), and ZnCl_2_ (triangles, *c*_Lig_ = *c*_Gd_^3+^ = 1.001 mM at pH = 4.81). All solutions were buffered using 50 mM DMP. The solid lines show the fit of the data for stability constant determination.

The stability of the Gd^3+^ complex with *CHX*OCTAPA^4–^ was reported in a previous paper.^38^ This complex was found to be nearly fully formed at pH ∼ 2, which complicates stability constant determination using potentiometric titrations. The stability of the complex could be determined using the relaxometric method with aqueous solutions buffered with dimethylpiperazine (DMP).^40^ Relaxivity, *r*_1p_, refers to the paramagnetic longitudinal relaxation rate enhancement of water protons for a 1 mM concentration of the paramagnetic Gd^3+^ ion.^41^ The relaxivity of [Gd(H_2_O)_8_]^3+^ is considerably higher than that of the [Gd(*CHX*OCTAPA)]^−^ complex, and thus complex dissociation provoked by the addition of competing metal ions (La^3+^, Yb^3+^, or Zn^2+^) causes an important increase of the relaxation rate of water protons (Figure 1). These experiments were carried out using the batch method and long equilibration times (4 weeks) to ensure that thermodynamic equilibrium was attained. The titration profiles observed for La^3+^ and Yb^3+^ are remarkably different, with addition of Yb^3+^ inducing a rather sharp inflection point. This anticipates that the stability of the Yb^3+^ complex is slightly higher than that of the La^3+^ analogue. The fit of the relaxation data confirms this qualitative analysis, yielding stability constants of log *K*_YbL_ = 19.60(5) and log *K*_LaL_ = 18.09(3).

The stability of the complexes with *CHX*OCTAPA^4–^ experiences a slight increase from La^3+^ to Gd^3+^ as the charge density of the metal ion increases. This is the most common trend observed for Ln^3+^ complexes,^42^ though it is often more pronounced than observed here.^19^ Only a few cases of reversed stability were reported for the complexes of macrocyclic ligands.^43−45^ The complexes with Gd^3+^ and Yb^3+^ present very similar stability. The complexes with DTPA^5–^ present a similar trend, with an initial increase in stability for the light lanthanide ions, the stability constants becoming nearly constant for the heaviest lanthanides (Table 1).^20,46^

**Table 1:** Protonation and Stability Constants of the Metal Complexes Formed with *CHX*OCTAPA^4–^ and Related Ligands (25 °C, 0.15 M NaCl)

	*CHX*OCTAPA^4–^	OCTAPA^4–^a	DTPA^5–^	DO3A^3–^g	DOTA^4–^
log *K*_LaL_	17.82(1); 18.09(3)^k^	19.92	19.49^e^	18.63	21.7^i^
log *K*_LaHL_	2.00(1)		2.60^e^		
log *K*_LaH-1L_	12.75(4)				
log *K*_GdL_	19.92(1)	20.23	22.03^d^/22.46^e^	21.56/19.06^h^	24.7^i^
log *K*_GdHL_	1.02(4)		1.96^d^/2.39^e^		
log *K*_GdH-1L_	12.45(2)				
log *K*_YbL_	19.65(1), 19.60(5)^k^	19.90^b^			
log *K*_YbHL_	1.89(2)				
log *K*_YbH-1L_	12.24(2)				
log *K*_LuL_		20.49/20.08^c^	22.44^e^	21.44	25.4^i^
log *K*_LuHL_			2.18^e^		
log *K*_MgL_	5.96(1)	6.12	9.27^e^	11.64	11.49^g^
log *K*_MgHL_	6.03(3)	5.24	6.85^e^		
log *K*_MgH2L_		4.54			
log *K*_CaL_	8.42(2)	9.55/9.4	10.7^f^	12.57	16.11^g^
log *K*_CaHL_	4.83(5)	3.92	6.11^f^	4.60	
log *K*_CaH2L_	4.57(6)	2.56			
log *K*_Ca2L_	3.88(7)	1.55			
log *K*_ZnL_	16.97(3)	18.91	17.58^d^	21.57	20.21^g^
log *K*_ZnHL_	4.04(3)	3.91	5.37^d^	3.47	
log *K*_ZnH2L_	3.15(2)	3.54	2.38^d^	2.07	
log *K*_ZnH3L_	1.34(4)				
log *K*_ZnH-1L_	11.63(7)				
log *K*_Zn2L_	3.99(5)	2.3	4.33^d^		
log *K*_Zn2HL_	3.26(4)				
log *K*_Zn2L(OH)_	7.63(4)				
log *K*_Zn2L(OH)2_	8.39(2)				
log *K*_CuL_	20.76(6)^j^	22.08	23.40^d^	25.75	24.83^g^
log *K*_CuHL_	4.02(9)^j^	3.95	4.63^d^	3.65	
log *K*_CuH2L_	4.07(2)^j^	3.21	2.67^d^	1.69	
log *K*_CuH3L_			2.03^d^		
log *K*_CuH-1L_	12.26(5)^j^				
log *K*_Cu2L_	5.64(6)^j^	3.2	6.56^d^		
log *K*_Cu2HL_	3.33(6)^j^		2.20^d^		
log *K*_Cu2L(OH)_	7.80(11)^j^				
log *K*_Cu2L(OH)2_	9.10(11)^j^				

a Data from ref (31) in 0.15 M NaCl unless otherwise indicated.

b Data in 0.16 M NaCl from ref (29).

c Data from ref (34).

d Data in 0.15 M NaCl from ref (20).

e Data in 0.1 M KCl from ref (46).

f Data in 0.1 M NaCl from ref (46).

g Data in 0.1 M KCl from ref (48) unless otherwise stated.

h Data in 0.15 M NaCl from ref (49).

i Data in 0.1 M NaCl from ref (50).

j Data obtained by simultaneous fitting of UV–vis and pH-potentiometry titration data obtained at 1:1 and 2:1 metal-to-ligand ratio.

k Determined using relaxometric titrations.

The stability constants determined for the Ln^3+^ complexes of *CHX*OCTAPA^4–^ by different methods (pH-potentiometry and ^1^H-relaxometry) are in excellent agreement, being comparable with those reported for the analogues with OCTAPA^4–^ (Table 1).^29,31^ This indicates that the replacement of the ethylenediamine spacer by a more rigid cyclohexyl group does not have a significant impact on complex stability, as observed recently for uranyl complexes.^47^ The stability constants characterizing the Ln^3+^ complexes of *CHX*OCTAPA^4–^ are similar to those of DO3A^3–^,^48,49^ but remain lower than those reported for the analogous DOTA^4–^ complexes.^48,50^ We note that the stability constants determined in 0.1 M KCl and 0.15 M NaCl for the Gd^3+^ complex of DTPA^5–^ are in good agreement, while there is a significant difference in the case of DO3A^3–^. This shows that Na^+^ cations form a relatively stable complex with DO3A^3–^ derivatives.^49^

Potentiometric titrations using a high ligand concentration (4.38 mM) in the presence of equimolar concentrations of La^3+^, Gd^3+^, and Yb^3+^ allowed for determining stability and protonation constants of the metal complexes. The log *K*_LnL_ values obtained for the La^3+^ and Yb^3+^ complexes are in excellent agreement with those obtained by relaxometry. These experiments afforded also the protonation constant of the complexes and also evidenced the formation of hydroxo complexes at high pH (log *K*_LnH-1L_ > 12, Figures S7 and S8). For Gd^3+^, the stability constant determined by potentiometry log *K*_GdL_ = 19.92(1) is slightly lower than that obtained previously by relaxometry (log *K*_GdL_ = 20.68).^38^ This slight discrepancy is related to the different set of ligand protonation constants used in the analysis.

The stability constants of the Mg^2+^ and Ca^2+^ complexes of *CHX*OCTAPA^4–^ could be determined using direct potentiometric titrations. Both cations form different protonated complex species in solution. Similarly, potentiometric titrations, using both 1:1 and 1:2 (M/L) stoichiometric ratios, allowed for determining the protonation constants of the complexes formed with Zn^2+^ and Cu^2+^. These metal ions also form relatively stable dinuclear complexes characterized by the corresponding equilibrium constants *K*_M2L_ and different hydroxo complexes at basic pH, yielding rather complex species distributions in solution (Table 1; see also Figures S1–S8, Supporting Information).

The stability constant of the Zn^2+^ and Cu^2+^ complexes is too high to be determined using direct pH potentiometric titrations, and thus UV–vis spectrophotometric experiments were carried out under acidic pH to determine the stability of the Cu^2+^ complex, following the changes of the d–d absorption band at *ca*. 710 nm with pH (Figure S9, Supporting Information). The stability of the Zn^2+^ complex was obtained by competition titration with Gd^3+^ using relaxometry. The log *K*_ML_ values characterizing the formation of the Ca^2+^, Zn^2+^ and Cu^2+^ complexes with *CHX*OCTAPA^4–^ are 1–2 log *K* units lower than those of the corresponding complexes formed with OCTAPA^4–^. This is in contrast to previous studies, which evidenced a gain in complex stability with small metal ions upon incorporation of rigid cyclohexyl groups.^56^ This imparts *CHX*OCTAPA^4–^ with a higher selectivity for the Ln^3+^ ions than OCTAPA^4–^ over potentially competing divalent metal ions in vivo.

### Photophysical Properties

**Figure 2:**
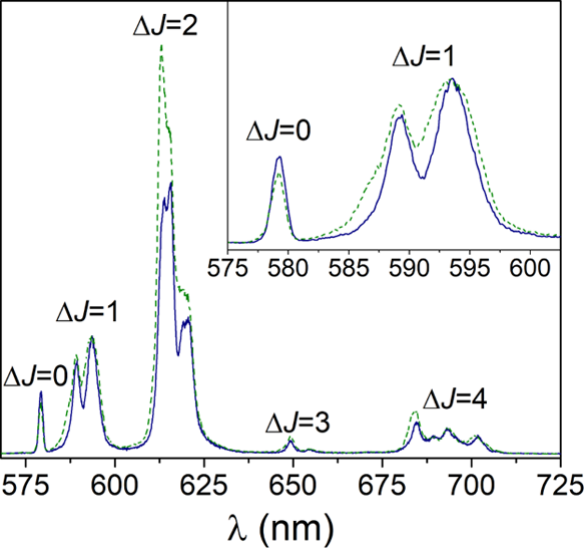
Emission spectra of the Eu^3+^ complexes with *CHX*OCTAPA^4–^ (blue solid line) and OCTAPA^4–^ (green dashed line) recorded in H_2_O solution at pH 7.1 (λ_ex_ = 279 nm; absorption and emission slits 1 nm, 10^–4^ M).

Ligands containing picolinate moieties were found to act as rather efficient sensitizers of the luminescent emission of Eu^3+^ and particularly Tb^3+^.^57−60^ Furthermore, picolinate units can be easily functionalized to tune their photophysical properties and provide efficient two-photon absorption.^61−63^ Thus, we have investigated the emission spectra of the [Ln(*CHX*OCTAPA)]^−^ (Ln = Eu, Tb) complexes in aqueous solution. The emission spectrum of the Eu^3+^ complex is dominated by the ^5^D_0_ → ^7^F_2_ (Δ*J* = 2) transition and presents a rather intense ^5^D_0_ → ^7^F_0_ transition (Figure 2). This spectral pattern is typical of Eu^3+^ in a coordination environment with a low symmetry.^64^ The lifetime of the excited ^5^D_0_ state measured in H_2_O solution (598 μs) is typical of Eu^3+^ complexes containing one coordinated water molecule (*q* = 1). Lifetime measurements recorded in D_2_O solutions afford a much longer lifetime of 2363 μs, as would be expected considering the efficient vibrational quenching of Eu^3+^ luminescence provoked by O–H oscillators of coordinated water molecules.^51^ The use of the empirical relationship proposed by Horrocks gives a *q* value of 1.0 ± 0.1, confirming the presence of a water molecule coordinated to the metal center (Table 2).^51^

**Table 2:** Spectroscopic Properties of [Ln(*CHX*OCTAPA)]^−^ and [Ln(OCTAPA)]^−^ Complexes Measured in Aqueous Solutions (pH 7.1)^c^

	[Eu(CHXOCTAPA)]^−^	[Eu(OCTAPA)]^−^	[Tb(CHXOCTAPA)]^−^	[Tb(OCTAPA)]^−^
λmax/nm	272	272	271	272
ε/M^–1^ cm^–1^	7.66 × 10^3^	7.50 × 10^3^	8.34 × 10^3^	9.36 × 10^3^
τ_H2O_/ms^a^	0.598	0.584	1.527	1.473
τ_D2O_/ms^a^	2.363	2.292	2.822	2.863
Φ_H2O_/%^b^	3.4	4.5	11	12
*Q*	1.0	1.1	1.3	1.2
τ_Rad_/ms	6.57	6.07		
Φ_Eu_/%	9.60	9.10		
η_sens_	0.37	0.47		

a λ_exc_ = 279 nm, estimated error ± 5%; *q*_Eu_ = 1.11(Δ*k*_obs_ – 0.31), ref (51); *q*_Tb_ = 5.0(Δ*k*_obs_ – 0.06), ref (52), with (Δ*k*_obs_ = 1/τ_H2O_ – 1/τ_D2O_).

b Determined using the trispicolinate complexes are standard, refs (53) and (54), λ_exc_ = 279 nm, estimated error ± 15%.

c Determined according to ref (55).

The emission spectrum recorded for the Tb^3+^ complex presents the ^5^D_4_ → ^7^F_J_ transitions expected for this metal ion, with *J* ranging from 6 to 3 (Figure S11, Supporting Information). The emission lifetimes of the excited ^5^D_4_ state recorded in H_2_O and D_2_O provide a *q* value of 1.3,^52^ in agreement with the results obtained for Eu^3+^.

The emission quantum yields measured for the Eu^3+^ (3.4%) and Tb^3+^ (11%) complexes were obtained using the corresponding trispicolinate complexes as secondary standards^53,54^ and are within the normal range reported for monohydrated chelates containing picolinate units.^57,65,66^ Thus, it is surprising that quantum yields one order of magnitude lower were reported by Platas-Iglesias et al. for the OCTAPA^4–^ analogues using quinine sulfate as standard (0.3 and 1.9% for Eu^3+^ and Tb^3+^ respectively).^26^ Furthermore, higher quantum yields for the latter complexes were presented in a PhD thesis,^67^ suggesting that the values reported by Platas-Iglesias were incorrect. We therefore reexamined the photophysical properties of the complexes with OCTAPA^4–^ (Table 2). These studies confirmed that the emission quantum yields of the Eu^3+^ and Tb^3+^ complexes with CHXOCTAPA^4–^ and OCTAPA^4–^ are very similar. The emission lifetimes measured for the two families of complexes are also very close, confirming the formation of *q* = 1 species in solution. The emission spectra recorded for the two Eu^3+^ complexes are rather similar, with a comparable splitting of the magnetic dipole ^5^D_0_ → ^7^F_1_ transition (∼140 cm^–1^). We notice that the hypersensitive Δ*J* = 2 transition is more intense in OCTAPA^4–^ than in CHXOCTAPA^4–^, while the intensity of the magnetic dipole Δ*J* = 1 transition remains very similar (Figure 2). This results in Δ*J* = 2/Δ*J* = 1 intensity ratios of 2.6 and 2.9 for the complexes with CHXOCTAPA^4–^ and OCTAPA^4–^, respectively. It has been shown that the relative intensity of these transitions is very sensitive to changes in the metal coordination environment.^68,69^ Because the nature of the donor atoms and the number of coordinated water molecules is identical in the two complexes, these results suggest that the two complexes are characterized by somewhat different coordination polyhedra.

Further insights into the sensitization efficiency of Eu^3+^ by the picolinate chromophores can be gathered by applying the methodology developed by Werts,^55^ which allows for estimating the radiative lifetime of the Eu^3+^-centered emission τ_Rad_, the metal-centered emission quantum yield Φ_Eu_ and the efficiency of the sensitization process η_sen_ (Table 2). The results of this analysis show that the observed emission quantum yields are limited by rather low Φ_Eu_ values associated to the quenching effect of the coordinated water molecule and a modest sensitization efficiency.^70−72^

### Structure of the Ln^3+^ Complexes in Solution

The structure of the [Ln(*CHX*OCTAPA)]^−^ complexes was investigated in D_2_O solutions at pH 7.0 using ^1^H and ^13^C NMR spectroscopy. We initiated the study by examining the NMR spectra of the diamagnetic La^3+^ and Lu^3+^ complexes. The spectra of the Lu^3+^ complex are consistent with the presence of a main isomer in solution and a *C*_1_ symmetry, as it shows 24 proton resonances and the same number of carbon signals (Figure S20, Supporting Information). A full attribution of the NMR data was attained with the aid of 2D COSY, HSQC, and HMBC experiments (Table S2, Supporting Information). The spectrum points to a rigid structure of the complex in solution, as the ^1^H spectrum displays well-resolved AB spin systems for the methylene protons. The spectra of the La^3+^ complex are, however, more complicated, evidencing the presence of two isomers in solution with very similar populations.

**Figure 3:**
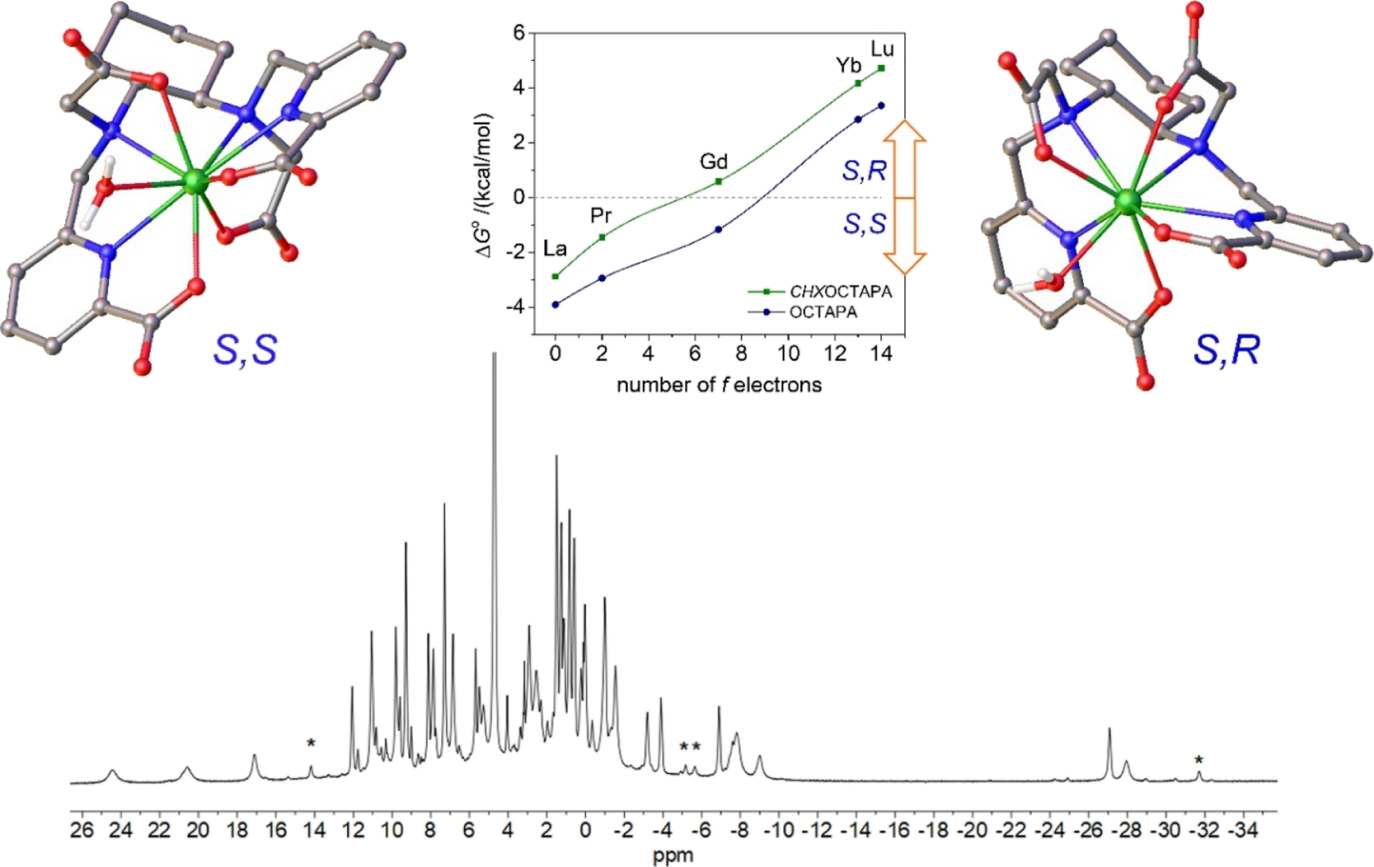
Top: Structures of the two isomers of [Gd(*CHX*OCTAPA) (H_2_O)]^−^·2H_2_O (second-sphere water molecules omitted for clarity) and relative energies calculated across the lanthanide series for the complexes with CHXOCTAPA^4–^ and OCTAPA^4–^. Bottom: ^1^H NMR spectrum of the Ce^3+^ complex recorded in D_2_O solution (300 MHz, 25 °C, pH 7.0). Asterisks denote a minor species present in solution.

The ^1^H NMR spectrum of the paramagnetic Ce^3+^ complex presents paramagnetically shifted signals in the approximate range 25 to −35 ppm (Figure 3). The spectrum is consistent with the presence of two isomers in solution, while only one isomer was observed previously for the Eu^3+^ complex.^38^ All together, these results indicate that the complexes of the large lanthanide ions (La–Ce) are present in solution in the form of two diastereoisomers, while only one isomer is observed for Eu^3+^ and the heavier Ln^3+^ ions. DFT calculations were performed to understand the nature of the two diastereoisomers present in solution for the [Ln(*CHX*OCTAPA)]^−^ complexes. A careful exploration of the potential energy surface provided two minimum energy geometries with rather small energy differences (Figure 3). These two minimum energy structures differ in the arrangement of the picolinate and acetate groups. One of the structures is characterized by nearly linear angles defined by the two pyridyl N atoms and the metal ion (N_PY_–Ln–N_PY_, ∼170°) and has been denoted as the trans isomer. Conversely, the second isomer (cis) is characterized by the coordination of picolinate (and acetate) groups on the same side of the metal ions, resulting in N_PY_–Ln–N_PY_ angles of ∼120°. The trans isomer is the most stable one at the beginning of the lanthanide series (La–Pr), while the cis isomer is predicted to be more stable for the second part of the lanthanide series (Gd–Lu). Analogous calculations performed for the [Ln(OCTAPA)]^−^ complexes provide a similar trend for the relative energies, though the cis isomer is stabilized later on along the series. As a result, our calculations predict that the most stable form for the [Gd(OCTAPA)]^−^ complex is the trans isomer, which is in nice agreement with the X-ray structure reported by Mazzanti.^27^ A trans structure was also established for the light Ln^3+^ complexes with OCTAPA^4–^ by analysis of the paramagnetic ^1^H NMR shifts.^26^ The cis isomer is characterized by different configurations of the amine N atoms (*S*,*R* or *R*,*S*), while these N atoms have the same configuration in the trans isomer (*S*,*S* or *R*,*R*, Figure 3).

The ^1^H NMR spectra of Yb^3+^ complexes encode structural information that can be used to validate structural models obtained with DFT calculations.^73^ The ^1^H NMR signals due to ligand nuclei in paramagnetic Yb^3+^ complexes experience large frequency shifts induced by the pseudocontact mechanism (δ^PC^), which is related to the anisotropy of the magnetic susceptibility associated to the 4f electrons.^74,75^ The pseudocontact shift can be expressed as in eq 1 when the reference frame coincides with the principal directions of the magnetic susceptibility tensor χ
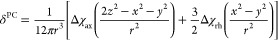
1where *r*^2^ = *x*^2^ + *y*^2^ + *z*^2^, *x*, *y*, and *z* are the Cartesian coordinates of a nucleus *i* relative to the location of a Yb^3+^ ion placed at the origin, and Δχ_ax_ and Δχ_rh_ are the axial and rhombic parameters of the symmetric magnetic susceptibility tensor.

**Figure 4:**
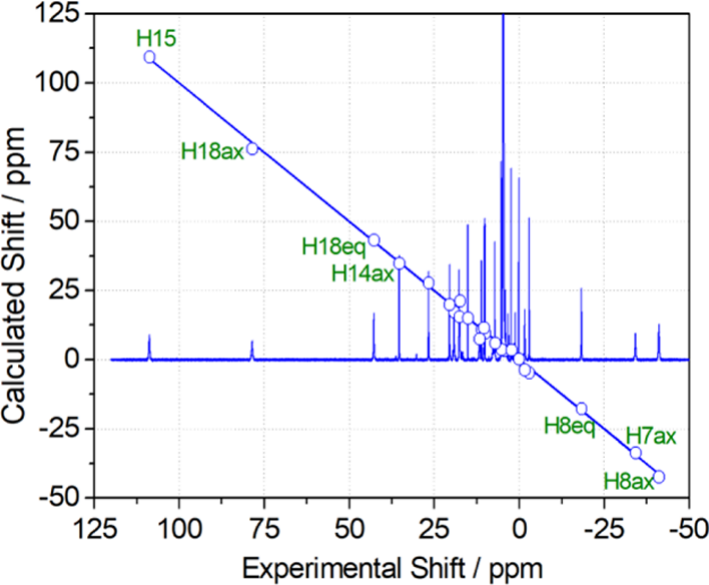
^1^H NMR spectrum of [Yb(*CHX*OCTAPA)]^−^ (300 MHz, 25 °C, pH 7.0) and plot of the calculated chemical shifts versus those obtained with eq 1 and the structure of the cis isomer. The line represents the identity line.

The ^1^H NMR spectrum of the Yb^3+^ complex of *CHX*OCTAPA is well resolved, presenting paramagnetically shifted resonances in the range +109 to −41 ppm (Figure 4). The spectrum was assigned on the basis of line-width analysis, as the paramagnetic contribution to the linewidths of ^1^H resonances depends on 1/*r*^6^.^76^ Thus, those protons located at shorter distances from the paramagnetic ion are characterized by broader resonances. Additional information for the assignment of the ^1^H NMR spectrum was gained from ^1^H,^1^H–COSY measurements, which show cross-peaks relating the protons of the pyridyl units, the geminal CH_2_ protons of the acetate and picolinate groups, and the protons of the cyclohexyl unit placed at a three-bond distance. The analysis of the paramagnetic shifts was accomplished by using eq 1, using the diamagnetic shifts observed for the Lu^3+^ analogue (Table S2, Supporting Information). Given the lack of any symmetry axis in the complex, the position of the magnetic axes cannot be anticipated. Thus, we performed a least squares fitting of the paramagnetic shifts to eq 1 by using five fitting parameters: The axial (Δχ_ax_/12π) and rhombic (Δχ_rh_/8π) parts of the magnetic susceptibility tensor and three Euler angles relating the input orientation and that of the magnetic susceptibility tensor. The structure of the complex obtained with DFT calculations was used as a structural model.

The agreement of the chemical shifts observed for the Yb^3+^ complex and those calculated with eq 1 (and the estimates of the diamagnetic shifts using the Lu^3+^ complex) is excellent, with deviations <4.2 ppm and a mean deviation of 1.26 ppm (Figure 4, see also Table S2, Supporting Information). This is confirmed by the agreement factor AF_j_ = 0.050, which is similar to or better than those reported previously and considered to be satisfactory (0.06–0.11).^77−81^ Lower agreement factors were also calculated for symmetrical systems, but in those cases, the fit of the data involved a low number of experimental chemical shifts.^73^ This analysis indicates that the structure of the cis isomer obtained with DFT represents a good approximation of the actual structure of the complex in solution. Conversely, an unacceptable fit was obtained by using the trans isomer as the structural model (AF_j_ = 0.363), with deviations of the experimental and calculated data of up to ∼34 ppm. As would be expected, the magnetic susceptibility tensor determined for the fit of the data for the cis isomer is rhombic, with Δχ_ax_/12π = −2379 ± 29 ppm Å^3^ and Δχ_rh_/8π = 919 ± 65 ppm Å^3^. The orientation of the magnetic axis is such that one of the picolinate lies close to the *yz* plane and one of the carboxylate groups on the *xz* plane (Figure S22, Supporting Information).

### ^1^H NMRD and ^17^O NMR Studies

**Figure 5:**
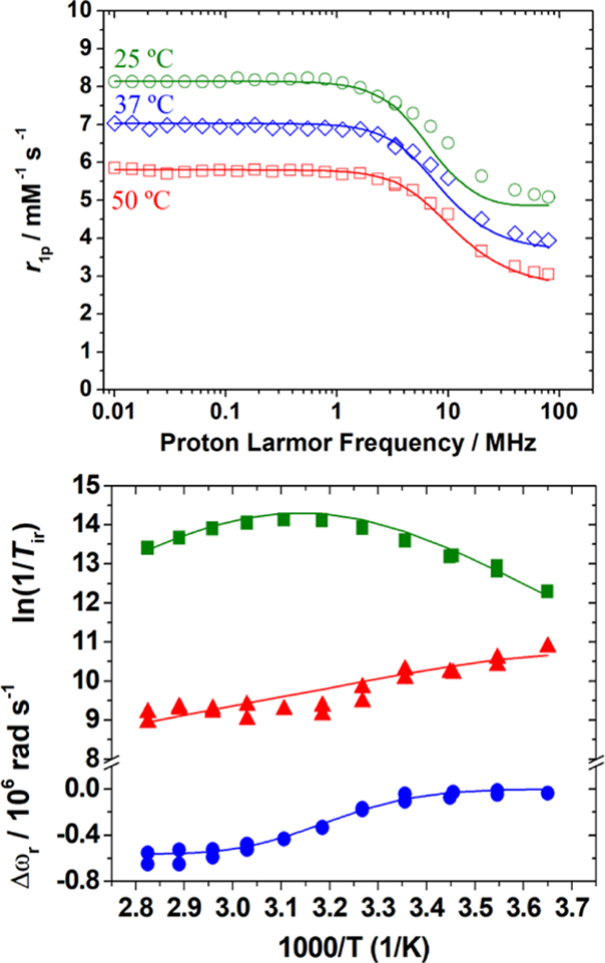
Top: ^1^H NMRD profiles recorded at different temperatures for [Gd(*CHX*OCTAPA)]^−^ (pH 7.27). Bottom: Reduced transverse (green ■) and longitudinal (red ▲) ^17^O NMR relaxation rates and ^17^O NMR chemical shifts (blue ●) measured for [Gd(*CHX*OCTAPA)]^−^ at 9.4 T (0.0199 mM, pH = 7.27). The lines represent the fit of the data as explained in the text.

The relaxivity of [Gd(*CHX*OCTAPA)]^−^ was investigated in the proton Larmor frequency range 0.01–80 MHz, corresponding to magnetic field strengths varying between 2.34 × 10^–4^ and 1.88 T (Figure 5). The relaxivities recorded at 20 MHz (Table 3) are slightly higher than those reported for [Gd(OCTAPA)]^−^, [Gd(DOTA)]^−^, and [Gd(DTPA)]^2–^, but still consistent with the presence of a water molecule in the inner coordination sphere, as indicated by emission lifetime measurements (see above). As expected, fast rotation of the complex in solution limits proton relaxivity, which decreases with increasing temperature. Because the inner-sphere contribution to ^1^H relaxivity is affected by a relatively large number of parameters, we have also recorded reduced longitudinal (1/*T*_1r_) and transverse (1/*T*_2r_) ^17^O NMR relaxation rates and reduced chemical shifts (Δω_r_) of an aqueous solution of the complex (19.9 mM, pH = 7.27). These studies provide independent information about some important parameters that control ^1^H relaxivity, especially the exchange rate of the coordinated water molecule(s) (*k*_ex_^298^) and the rotational correlation time (τ_R_^298^).^82^ The 1/*T*_2r_ values increase with decreasing temperature at high temperatures, reach a maximum at *ca*. 322 K, and then decrease. This is typical of systems that experience a changeover from a slow exchange regime at low temperature to a fast exchange condition at high temperature.^83^ The inflection point observed for the 1/*T*_2r_ values is also clearly visible in the chemical shift data.

**Table 3:** Parameters Obtained from the Simultaneous Analysis of ^17^O NMR and ^1^H NMRD Data

	*CHX*OCTAPA^4–^	OCTAPA^4–^b	DTPA^5–^c	DOTA^4–^c
*r*_1p_ at 25/37 °C, 20 MHz/mM^–1^ s^–1^	5.6/4.5	5.0/3.9	4.7/4.0	4.7/3.8
k_ex_^298^/10^6^ s^–1^	1.58 ± 0.09	5.0	3.3	4.1
Δ*H*^⧧^/kJ mol^–1^	54.6 ± 1.8	40.1	51.6	49.8
τ_RH_^298^/ps	75 ± 3	55^b^	58^b^	77
E_r_/kJ mol^–1^	19.5 ± 1.2	17.9	17.3	16.1
τ_v_^298^/ps	11.3 ± 0.06	12.6	25	11
E_v_/kJ mol^–1^	1.0^a^	1.0^a^	1.6	1.0^a^
Δ^2^/10^20^ s^–2^	1.04 ± 0.06	1.2	0.46	0.16
D_GdH_^298^/10^–10^ m^2^ s^–1^	20.0^a^	19	20	22
E_DGdH_/kJ mol^–1^	22^a^	30.1	19.4	20.2
A/ℏ/10^6^ rad s^–1^	–3.06 ± 0.08	–2.31	–3.8	–3.7
χ(1 + η^2/3^)^1/2^/MHz	10.7^a^	17^b^	14^b^	10
r_GdH_/Å	3.005^a^	2.969^a^	3.1^a^	3.1^a^
r_GdO_/Å	2.480^a^	2.54^a^	2.5^a^	2.5^a^
a_GdH_/Å	3.5^a^	3.4^a^	3.5^a^	3.5^a^
*q*^298^	1^a^	1^a^	1^a^	1^a^

a Parameters fixed during the fitting procedure.

b Data from ref (26).

c Data from ref (90).

A simultaneous fitting of the ^1^H NMRD and ^17^O NMR data of [Gd(*CHX*OCTAPA)]^−^ was performed using a well-established methodology that treats the inner-sphere contribution to relaxivity with the Solomon–Bloembergen–Morgan theory^84−86^ and the outer-sphere mechanism with the translational diffusion model proposed by Freed.^87^ The ^17^O NMR data were fitted with the standard Swift–Connick^88,89^ equations. Several parameters have been fixed during the fitting procedure: the number of water molecules coordinated to the Gd^3+^ ion was fixed to *q* = 1 on the basis of the luminescence lifetime measurements described above, the distance of closest approach for the outer-sphere contribution *a*_GdH_ was fixed at 3.5 Å, and the distances between the Gd^3+^ ion and the H and O atoms of the coordinated water molecule (*r*_GdH_ and *r*_GdO_) were set to the values obtained from DFT calculations. The value of the ^17^O quadrupole coupling constant χ(1 + η^2/3^)^1/2^ was also estimated using DFT calculations. In previous studies, the quadrupole coupling constant was allowed to vary during the fitting procedure,^90^ providing fitted values that deviated markedly from that obtained for acidified water (7.58 MHz).^91^ As a result, the fits of the data gave low rotational correlation times τ_R_ (Table 3). However, it has been demonstrated that coordination to Gd^3+^ provokes negligible changes in the quadrupole constant.^92^ Our calculations provided χ = 7.77 MHz and an asymmetry parameter η = 0.84 (χ = 6.68 MHz and η = 0.93 for pure water), yielding a χ(1 + η^2/3^)^1/2^ value of 10.7 MHz. Additional parameters that were fixed to reasonable values were the diffusion coefficient D_GdH_^298^ (20 × 10^–10^ m^2^ s^–1^), its activation energy *E*_DGdH_ (22 kJ mol^–1^), and the activation energy for the modulation of the zero field splitting interaction (*E*_v_ = 1 kJ mol^–1^). The rotational correlation time τ_R_ affects both the *T*_1_^17^O relaxation rates and *r*_1p_ values. However, it has been shown that rotational correlation time characterizing the Ln–H_water_ vector is ∼65% shorter than that of the Ln–O_water_ vector.^93^ Thus, we included in the fitting two different τ_R_ values with the constraint that τ_RH_/τ_RO_ = 0.65.

An excellent fit of the ^17^O NMR and ^1^H NMRD data was obtained using the parameters listed in Table 3. The water exchange rate *k*_ex_^298^ is lower than those determined for the complexes with OCTAPA^4–^ and DTPA^5–^. A faster average exchange rate was also determined for the complexes with DOTA^4–^, though in the latter case two isomers with very different water exchange parameters are present in solution.^94^ The rigidity of the *CHX*OCTAPA^4–^ ligand likely increases the energy cost required to reach the transition state responsible for the water exchange process, resulting in a rather low water exchange rate.^95^ A similar effect was observed previously upon rigidification of OCTAPA derivatives incorporating phosphonate groups.^96^ The parameters characterizing the relaxation of the electron spin are very similar to those obtained for OCTAPA^4–^, as would be expected from the similar relaxivities observed at low magnetic fields (<1 MHz). Complexes with DOTA^4–^ derivatives display slower electron spin relaxation, as a result of lower squared zero field splitting energies (Δ^2^, Table 3). Finally, the value obtained for the hyperfine coupling constant *A*/ℏ is in excellent agreement with that estimated with DFT (3.10 × 10^6^ rad s^–1^), which provides support to the reliability of the analysis.

### Dissociation Kinetics

The slow dissociation of Ln^3+^ complexes is a key property for their application as both MRI contrast agents and radiopharmaceuticals. In the case of MRI contrast agents, there is an increasing concern on potential toxicity issues related to the release of Gd^3+^ in vivo.^97^ Radiopharmaceuticals are injected in low doses, and thus chemical toxicity problems are likely not an important concern. However, complex dissociation may have negative effects by reducing the amount of radioisotope that reaches the desired target, thereby exposing to radiation healthy tissue.^98^ In a previous paper, we analyzed the dissociation kinetics of the Gd^3+^ complex, which was found to be remarkably inert.^38^ Herein, we present a detailed analysis of the dissociation kinetics of the Lu^3+^ analogue, given the potential of ^177^Lu for therapeutic applications. We have shown recently that the dissociation kinetics of Ln^3+^ complexes may vary by several orders of magnitude across the lanthanide series, and thus the remarkable inertness of the Gd^3+^ complex does not necessarily ensure that the Lu^3+^ analogue behaves in a similar way.^99^

**Figure 6:**
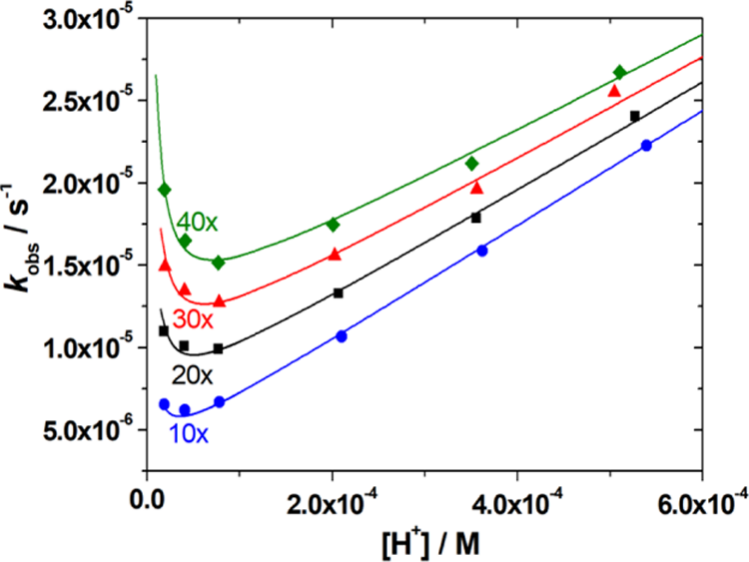
Plot of the pseudo-first-order rate constants measured for the [Lu(*CHX*OCTAPA)]^−^ as a function of H^+^ ion concentration (50 mM DMP, 25 °C, 0.15 M NaCl) using different metal ion excess [10× (5.53 mM), 20× (11.07 mM), 30× (16.60 mM), and 40× (22.14 mM) was applied with pH = 3.30, 3.50, 3.80, 4.17, and 4.49]. The solid lines represent the fits of the data to eq 7.

The dissociation of the Lu^3+^ complex with *CHX*OCTAPA^4–^ was investigated by following the rates of exchange reactions taking place with Cu^2+^ at different proton concentrations (pH 3.30–4.72). The reactions were monitored in the presence of at least 10-fold Cu^2+^ excess to ensure pseudo first-order conditions. The observed rate constants display a rather unusual behavior, as increasing *c*_H^+^_ provokes a slight initial decrease of the dissociation rates, which subsequently increase at higher *c*_H^+^_ values. Furthermore, Cu^2+^ is also affecting significantly the complex dissociation rates (Figure 6). This indicates that the Lu^3+^ complex experiences dissociation by following the proton-assisted and metal-assisted pathways, the latter involving formation of a hetero-dinuclear complex. The dinuclear complex appears to form a hydroxo complex at relatively low pH that is responsible for the increase in *k*_obs_ values in the low proton concentration side (Figure 6). Thus, the dissociation of the complex can be expressed as in eq 2, where *k*_0_ is the rate constant characterizing the spontaneous dissociation, *k*_H_ is the rate constant characterizing the proton-assisted dissociation, and *k*_Cu_ and *k*_Cu_^OH^ are associated with the metal-assisted dissociation pathways, the latter with the formation of a hydroxo dinuclear complex.
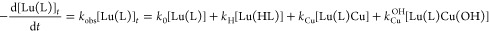
2

Considering that the total concentration of complexed Lu^3+^ is given by eq 3 and the equilibrium constants defined by eqs 4–6, the rate constants can be expressed as in eq 7, where *k*_1_ = *k*_H_ × *K*_H_, *k*_3_^Cu^ = *k*_Cu_*K*_Cu_, and *k*_6_^Cu^ = *k*_Cu_^OH^*K*_Cu(OH)_*K*_Cu_.
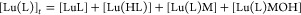
3
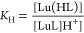
4
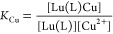
5
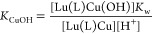
6
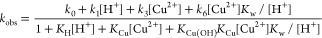
7

Attempts to fit the data to eq 7 including *k*_0_ as fitting parameter provided a small negative value, which indicates that spontaneous dissociation does not play any role under the conditions used for kinetic experiments. Furthermore, it is difficult to estimate the rate constant characterizing the spontaneous reaction pathway within the same pH range where the dissociation of the Lu(L)Cu(OH) complex takes place, as the latter acts as a competitive dissociation path to the spontaneous dissociation. A similar situation occurred for *K*_H_, revealing that the *K*_H_[H^+^] term in the denominator of eq 7 has a negligible contribution to *k*_obs_. This is expected considering the low protonation constants determined using potentiometry (log *K*_LnH_ in the range 1–2, Table 1) and the relatively low proton concentrations used for kinetic experiments (<10^–6^ M, Figure 6). The results of the fit are shown in Table 4, together with a comparison with the data reported previously for the Gd^3+^ complexes of *CHX*OCTAPA^4–^,^38^ OCTAPA^4–^,^31^ DTPA^5–^,^25^ and DO3A^3–^.^48^ It is worth mentioning that the dissociation pathway through formation of a hydroxo dinuclear species was not detected for the Gd^3+^ analogue, which was investigated in approximately the same pH range. The formation of hydroxo complexes is more likely to occur as the size of the lanthanide ion decreases across the series due to the lanthanide contraction, as indicated by the corresponding hydrolysis constants (log *K*_Ln(OH)_ = −7.83 and −7.27 for Gd^3+^ and Lu^3+^, respectively).^100^ Alternatively, the structural change occurring close to the center of the lanthanide series could be responsible for the different behavior of the Gd^3+^ and Lu^3+^ complexes.

**Table 4:** Rate and Equilibrium Constants Characterizing the Dissociation of the CHXOCTAPA^4–^ Complexes and Related Systems (25 °C)

	[LuCHXOCTAPA]^−^	[GdCHXOCTAPA]^−^a	[GdOCTAPA]^−^b	[GdDTPA]2^–^c	[GdDO3A]^d^
k_1_/M^–1^ s^–1^	3.74 ± 0.06 × 10^–2^	1.60 × 10^–2^	11.8	0.58	0.023
k_2_/M^–2^ s^–2^			2.5 × 10^4^	9.7 × 10^4^	
k_3_^Cu^/M^–1^ s^–1^	6.3 ± 0.3 × 10^–4^	6.8 × 10^–4^	22.5	0.93	
k_6_^Cu^/M^–2^ s^–1^	5.1 ± 0.3 × 10^5^		5.0 × 10^9^		
*K*_H_		737	2.6	100	
*K*_Cu_	12.1 ± 1.6	48		13	
t_1/2_/h^e^	876	1.49 × 10^5^	0.15	202	2.10 × 10^5^

a Data from ref (38).

b Data from ref (26).

c Data from ref (25).

d Data from ref (48).

e Half-lives determined at pH 7.4 and [Cu^2+^] = 1 μM.

The rate constants shown in Table 4 indicate that the Gd^3+^ and Lu^3+^ analogues present similar inertness with respect to their dissociation following the proton-assisted and metal-assisted pathways, as judged by the values of the *k*_1_ and *k*_3_^Cu^ rate constants. However, the metal-assisted pathway with the formation of a hydroxo complex, characterized by *k*_6_^Cu^, plays an increasingly important role in the dissociation of the complex as the concentration of OH^–^ increases. As a result, this pathway is mainly responsible for complex dissociation at pH 7.4, a situation that is clearly reflected in the half-lives of the complex calculated at pH 7.4 using [Cu^2+^] = 1 μM (Table 4). Nevertheless, the half-life estimated for [Lu(*CHX*OCTAPA)]^−^ remains three times longer than that of [Gd(DTPA)]^2–^, but clearly shorter than that of the macrocyclic complex [Gd(DO3A)]. The effect that the rigid cyclohexyl unit has in improving kinetic inertness is also obvious when comparing the half-lives of *CHX*OCTAPA^4–^ and OCTAPA^4–^ derivatives.

## Conclusions

The present contribution has shown that the octadentate *CHX*OCTAPA^4–^ ligand forms fairly stable complexes with the Ln^3+^ ions, with stability constants in the range log *K*_LnL_ ∼ 17.8–19.7. The presence of the rigid cyclohexyl ring causes a slight increase of the selectivity of the ligand for the Ln^3+^ ions over Cu^2+^ and Zn^2+^. The picolinate units are rather efficient in sensitizing the Eu^3+^ and particularly Tb^3+^ luminescence, with emission quantum yields comparable to those of the OCTAPA^4–^ analogues. The complexes are monohydrated (*q* = 1) in solution, as indicated by emission lifetime measurements. The exchange rate of the water molecule coordinated to Gd^3+^ (as confirmed by ^17^O NMR studies) is rather low when compared with the OCTAPA^4–^, DTPA^5–^, and DOTA^4–^ analogues, likely as a result of the rigid structure of the complex.

The coordination chemistry reported in this paper provided two unexpected results. First, the analysis of the structural information encoded by the pseudocontact shifts, induced by Yb^3+^, demonstrate that this complex presents an unusual cis structure in which the amine N atoms adopt *S*,*R* configurations. DFT calculations show that this conformation is stabilized across the lanthanide series over the *trans R*,*R* (or *S*,*S*) conformation. The presence of the cyclohexyl group causes a significant stabilization of the *S*,*R* conformation. A second unexpected effect was observed when investigating the dissociation of the Lu^3+^ complex in the presence of exchanging Cu^2+^ ions. Complex dissociation at physiological pH was found to occur mainly through the metal-assisted mechanism that involves the formation of a hydroxo complex, a pathway that was not observed previously for the Gd^3+^ analogue. We hypothesize that this pathway may be relevant for the dissociation of complexes of acidic cations relevant for radiopharmaceutical applications (i.e., Sc^3+^).

## Experimental and Computational Section

### Materials

The H_4_*CHX*OCTAPA and H_4_OCTAPA ligands were prepared as described in previous papers.^31,38^ All other chemicals and solvents were purchased from commercial sources and used without further purification. The complexes used for NMR and photophysical studies were prepared by mixing stoichiometric amounts of the ligand and the corresponding Ln(OTf)_3_ salts and subsequent adjustment of the pH with diluted NaOH/NaOD solutions.

### NMR Spectroscopy

^1^H NMR spectra were recorded at 25 °C in solutions of the complexes in D_2_O using Bruker Avance 300 or Bruker ARX400 spectrometers. Chemical shifts were referenced by using the residual solvent HDO proton signal (δ = 4.79 ppm).^101^

The ^1^H NMRD measurements were carried out by using a Stelar SMARTracer Fast Field Cycling relaxometer (0.01–10 MHz) and a Bruker WP80 NMR electromagnet adapted to variable field measurements (20–80 MHz) controlled by a SMARTracer PC-NMR console. The NMRD profiles of the [Gd(*CHX*OCTAPA)]^−^ complex (*c*_complex_ = 2.69 mM) were recorded in aqueous solution at three different temperatures (25, 37 and 50 °C) in the presence of 4-(2-hydroxyethyl)-1-piperazineethanesulfonic acid buffer (25 mM, pH = 7.27) to maintain the pH constant. The temperature of the samples was managed by a VTC91 temperature control unit (calibrated by a Pt resistance temperature probe) and maintained by gas flow.

Transverse and longitudinal ^17^O relaxation rates (1/*T*_2_, 1/*T*_1_) and chemical shifts were measured in aqueous solutions of [Gd(*CHX*OCTAPA)]^−^ (0.0199 mM, pH = 7.27) in the temperature range 274–354 K on a Bruker Avance 400 (9.4 T, 54.24 MHz) spectrometer. The temperature was calculated according to previous calibration with ethylene glycol and methanol.^102^ An acidified water solution (HClO_4_, pH 3.3) was used as an external reference. Longitudinal relaxation times (*T*_1_) were obtained by the inversion–recovery method, and transverse relaxation times (*T*_2_) were obtained by the Carr–Purcell–Meiboom–Gill spin-echo technique.^103^ The technique used for ^17^O NMR measurements on Gd^3+^ complexes has been described elsewhere.^104^ The samples were sealed in glass spheres fitted into 10 mm NMR tubes to avoid susceptibility corrections of the chemical shifts.^105^ To improve the sensitivity, ^17^O-enriched water (10% H_2_^17^O, CortecNet) was added to the solutions to reach around 2% enrichment. The ^17^O NMR data were treated according to the Solomon–Bloembergen–Morgan theory of paramagnetic relaxation. The least-squares fit of the ^17^O NMR and ^1^H NMRD data was performed using Micromath Scientist version 2.0 (Salt Lake City, UT, USA).

### Absorption and Emission Electronic Spectroscopy

The absorption spectra of the Eu^3+^ and Tb^3+^ complexes were recorded with a Jasco V-650 spectrometer using 0.2 cm quartz cells. Steady-state emission spectra were obtained with a Horiba FluoroMax Plus-P spectrofluorometer using a 150 W ozone-free xenon arc lamp as the excitation source, a R928P photon counting emission detector, and an integration time of 0.1 s. Luminescence lifetimes were measured using the time-correlated single photon counting technique and a pulsed xenon flash lamp as the excitation source. Quantum yields were determined using the Cs_3_[Ln(pic)_3_] complexes (pic = 2,6-dipicolinate, Ln = Eu or Tb) as standards (Φ_Eu_ = 24% in TRIS, pH 7.4, 7.5 × 10^–5^ M; Φ_Tb_ = 22% in TRIS, pH 7.4, 6.5 × 10^–5^ M).^53,54^

### Equilibrium Studies

The chemicals (MCl_2_ and LnCl_3_ salts) used in the studies were of the highest analytical grade obtained from commercial sources (Sigma-Aldrich and Strem Chemicals Inc.). The concentration of the stock solutions was determined by complexometric titration using a standardized Na_2_H_2_EDTA solution and appropriate indicators (Patton & Reeder (CaCl_2_), Eriochrome Black T (MgCl_2_), xylenol orange (ZnCl_2_ and LnCl_3_), and murexide (CuCl_2_)).

The pH potentiometric titrations were carried out with a Metrohm 888 Titrando titration workstation using a Metrohm-6.0233.100 combined electrode. The titrated solutions (6.00 mL) were thermostated at 25 °C, and samples were stirred and kept under an inert gas atmosphere (N_2_) to avoid the presence of CO_2_. The calibration of the electrode was performed by a two-point calibration [KH-phthalate (pH = 4.005) and borax (pH = 9.177) buffers] routine. The calculation of [H^+^] from the measured pH values was performed with the use of the method proposed by Irving et al.^106^ by titrating a 0.01 M HCl solution (*I* = 0.15 M NaCl) with a standardized NaOH solution. The differences between the measured (pH_read_) and calculated pH (−log [H^+^]) values were used to obtain the equilibrium H^+^ concentrations from the pH data obtained in the titrations. The ion product of water (p*K*_W_ = 13.847) was determined from the same experiment in the pH range 11.2–11.85.

The concentration of the *CHX*OCTAPA^4−^ chelator was determined by pH potentiometric titration, comparing the titration curves obtained in the presence and absence of high Ca^2+^ excess (the concentration of the ligand in the titration was 4.38 mM). The protonation constants of *CHX*OCTAPA^4−^, the stability and protonation constants of the complexes formed with Mg^2+^, Ca^2+^, Cu^2+^ and Zn^2+^, as well as those of La^3+^, Gd^3+^, and Yb^3+^ were also determined by pH potentiometric titration. The metal-to-ligand concentration ratios were 1:1 and 2:1 (the concentration of the ligand in these titrations was generally 2.50–3.00 mM). The pH potentiometric titration curves were measured in the pH range 1.70–11.85, while 122–356 mL NaOH-pH data pairs were recorded and fitted simultaneously.

Due to the high conditional stability of [Cu(*CHX*OCTAPA)]^2–^, the formation of the complex was complete (nearly 100%) even at pH = 1.75 (starting point of the pH potentiometric titrations). For this reason, 12 out-of-cell (batch) samples containing a slight excess of ligand and the Cu^2+^ ion were prepared ([L] = 3.110 mM, [Cu^2+^] = 3.065 mM, 25 °C, 3.0 M (Na^+^ + H^+^)Cl^–^). The samples, whose acidity was varied in the concentration range of 0.1005–3.007 M, were equilibrated for 1 day before recording the absorption spectra at 25 °C in Peltier thermostated semimicro 1 cm Hellma cells using a Jasco V-770 UV–vis–NIR spectrophotometer. The molar absorptivity of the [Cu(*CHX*OCTAPA)]^2–^ complex was determined at 25 wavelengths (600–840 nm range) by recording the spectra of 1.501 × 10^–3^, 3.002 × 10^–3^, and 4.503 × 10^–3^ M solutions of the complex, while for the Cu^2+^ ion, previously published molar absorptivity values (determined under identical conditions) were used for data fitting.^107^ The molar absorption coefficients of the protonated [CuH(*CHX*OCTAPA)]^−^ and [CuH_2_(*CHX*OCTAPA)] species were calculated during data refinement (UV–visible and pH potentiometric titration curves obtained at various metal to ligand concentrations were fitted simultaneously). The protonation (ligand and complexes) and stability constants (complexes) were calculated from the titration data with the PSEQUAD program.^108^

Stability constants of the [Zn(*CHX*OCTAPA)]^2–^, [La(*CHX*OCTAPA)]^−^, and [Yb(*CHX*OCTAPA)]^−^ complexes were also determined by following the competition reaction of these metal ions with Gd^3+^ for the ligand, in a similar manner as it was performed for the [M(OCTAPA)]^4–^ complexes.^31^ A total of 9–11 samples containing nearly 1 mM [Gd(*CHX*OCTAPA)]^−^ and 0.5–20.0 mM (La^3+^), 0.25–35.0 mM (Yb^3+^), or 0.5–20 mM (Zn^2+^) metal chlorides were prepared and equilibrated at constant pH (4.69 for La^3+^, 4.79 for Yb^3+^, and 4.81 for Zn^2+^). Longitudinal relaxation times of the samples were measured after 4 weeks (and repeated 4 weeks later to make sure that the equilibrium had been reached) and the formation constants determined by using the relaxivities of the Gd^3+^ aqua ion and [Gd(*CHX*OCTAPA)]^−^ (13.26 and 6.16 mM^–1^ s^–1^ at 25 °C and 0.49 T, respectively).^38^

### Kinetic Studies

The rates of the metal exchange reactions involving the [Lu(*CHX*OCTAPA)]^−^ complex and Cu^2+^ were studied by using UV–vis spectrophotometry following the formation of the [Cu_2_(*CHX*OCTAPA)] complex. The conventional UV–vis spectroscopic method was applied to follow the decomplexation reactions of [Lu(*CHX*OCTAPA)]^−^, as these reactions were very slow even at relatively low pH (in the pH range 3.27–4.39). The absorbance versus time kinetic curves were acquired by using a Jasco V-770 UV–vis–NIR spectrophotometer equipped with Peltier thermostatted multicell holder. The temperature was maintained at 25 °C, and the ionic strength of the solutions was kept constant by using 0.15 M NaCl. For keeping the pH constant, 50 mM DMP buffer was used (log *K*_2_^H^ = 4.19(5) as determined by using pH-potentiometry at 25 °C with the use of 0.15 M NaCl ionic strength). The exchange reactions were followed continuously at 300 nm for 4–5 days (80–95% conversion) and occasionally (one or two readouts per day) for another 5–7 days. The absorbance readings at equilibrium were determined 3–4 weeks after the start of the reaction depending on the pH of the samples (8–10 times longer than the half-life of the reaction). The concentration of the [Lu(*CHX*OCTAPA)]^−^ chelate was 0.52 mM, while the Cu^2+^ ion was applied at high excess (10.6–42.6 fold) in order to ensure pseudo-first order conditions. The pseudo-first-order rate constants (*k*_obs_) were calculated by fitting the absorbance–time data pairs to eq 8
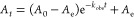
8where *A*_*t*_, *A*_0_, and *A*_e_ are the absorbance at time *t*, at the start, and at equilibrium, respectively. The pseudo-first-order rate constants were fitted with the computer program Micromath Scientist, version 2.0 (Salt Lake City, UT, USA) by using a standard least-squares procedure.

### Computational Studies

The geometries of the [Ln(OCTAPA)(H_2_O)]^−^·2H_2_O and [Ln(*CHX*OCTAPA)(H_2_O)]^−^·2H_2_O systems (Ln = La, Pr, Gd, Yb, or Lu) were optimized using DFT calculations with the M062X^109^ exchange correlation functional. Two explicit second-sphere water molecules were considered in these models for a more appropriate description of the interaction between the metal ion and the coordinated water molecule.^110^ Relativistic effects were considered with the pseudopotential approximation using either the large-core quasi-relativistic effective core potentials (ECP) developed by Dolg et al. ([Kr]4d^10^4f^n^ core)^111^ and the (14s6p5d)/[2s1p1d]-GTO valence basis sets (Ln = Pr, Gd, Yb and Lu) or the small-core quasi-relativistic ECP (1s–3d electrons in the core)^112^ and the associated (42s26p20d8f)/[3s2p2d1f] valence basis set (Ln = La). All other atoms were described using the standard 6-311G(d,p) basis set. Bulk solvent effects were incorporated using the integral equation formalism implementation of the polarized continuum model.^113^ Frequency calculations were performed to confirm that geometry optimizations provided local energy minima on the corresponding potential energy surfaces. All pseudopotential calculations were carried out with the Gaussian 16 program.^114^

Hyperfine and quadrupole coupling constants^115^ of the O atoms of water molecules coordinated to Gd^3+^ were estimated with DFT using the ORCA4 program package^116,117^ and a Gaussian finite model.^118^ In these calculations, we used the hybrid *meta*-GGA TPSSh functional,^119^ which was found to provide good estimates of hyperfine coupling constants in Gd^3+^^110,120^ and other metal complexes.^121^ Relativistic effects were introduced with the Douglas–Kroll–Hess (DKH2) method,^122,123^ using the SARC2-DKH-QZVP^124^ for Gd and the DKH-def2-TZVPP^125^ basis set for all other atoms. The resolution of identity and chain of spheres^126,127^ algorithm was used to speed up the calculation with the aid of auxiliary basis sets generated with the Autoaux^128^ procedure for Gd and the SARC/J auxiliary basis set for all other atoms (decontracted Def2/J).^129^ Bulk solvent effects were considered with the SMD solvation model developed by Truhlar.^130^

## Supporting Information Available

Absorption and emission spectra, NMR spectra, speciation diagrams, analysis of the Yb^3+^-induced paramagnetic shifts, bond distances, and optimized geometries obtained with DFT (PDF)
