# Supplementary material for: Rigidified Derivative of the Non-macrocyclic Ligand H4OCTAPA for Stable Lanthanide(III) Complexation
Source: Inorg Chem. 2022 Mar 11;61(12):5157–71. doi: 10.1021/acs.inorgchem.2c00501 (PMC8965877; doi:10.1021/acs.inorgchem.2c00501)
Supplement: Supplementary file 1 — ic2c00501_si_001.pdf [file ic2c00501_si_001.pdf]

Supporting Information

A Rigidified Derivative of the Non-Macrocyclic Ligand H4OCTAPA for Stable Lanthanide(III) Complexation

Fátima Lucio-Martínez,† Zoltán Garda,‡ Balázs Váradi,‡,≠ Ferenc Krisztián Kálmán,‡ David Esteban-Gómez,† Éva Tóth,§ Gyula Tircsó,*,‡ and Carlos Platas-Iglesias*,†

† Centro de Investigacións Científicas Avanzadas (CICA) and Departamento de Química, Facultade de Ciencias, Universidade da Coruña, 15071 A Coruña, Galicia, Spain

‡ Department of Physical Chemistry, ≠ Doctoral School of Chemistry, University of Debrecen, H-4010 Debrecen, Egyetem tér 1, Hungary

§ Centre de Biophysique Moléculaire, CNRS UPR 4301, Université d'Orléans, rue Charles Sadron, 45071 Orléans, Cedex 2, France

Email: carlos.platas.iglesias@udc.es (C. P.-I.); gyula.tircso@science.unideb.hu (Gy. T.)

Table S1. Comparison of the protonation constants of *CHX*OCTAPA^{4-} determined in this work with those reported in the literature (25 °C, 0.15 M NaCl)....5

Figure S1: Species distribution curves calculated for the Mg^{2+}:*CHX*OCTAPA^{4-} system (c_{Lig}=c_{Mg2+}=10^{-3} M). 6

Figure S2: Species distribution curves calculated for the Ca^{2+}:*CHX*OCTAPA^{4-} system (c_{Lig}=c_{Ca2+}=10^{-3} M). 6

Figure S3: Species distribution curves calculated for the Cu^{2+}:*CHX*OCTAPA^{4-} system (c_{Lig}=c_{Cu2+}=10^{-3} M). 7

Figure S4: Species distribution curves calculated for the Cu^{2+}:*CHX*OCTAPA^{4-} system (c_{Lig}=10^{-3} M, c_{Cu2+}=2 x 10^{-3} M). 7

Figure S5: Species distribution curves calculated for the Zn^{2+}:*CHX*OCTAPA^{4-} system (c_{Lig}=c_{Zn2+}=10^{-3} M). 8

Figure S6: Species distribution curves calculated for the Zn^{2+}:*CHX*OCTAPA^{4-} system (c_{Lig}=10^{-3} M, c_{Zn2+}=2 x 10^{-3} M). 8

Figure S7: Species distribution curves calculated for the La^{3+}:*CHX*OCTAPA^{4-} system (c_{Lig}=c_{La3+}=10^{-3} M). 9

Figure S8: Species distribution curves calculated for the Yb^{3+}:*CHX*OCTAPA^{4-} system (c_{Lig}=c_{Yb3+}=10^{-3} M). 9

Figure S9: Changes observed in the absorption spectrum of the Cu^{2+}:*CHX*OCTAPA^{4-} system (c_{Lig}= 3.110 mM, c_{Cu2+}= 3.065 mM, 25 °C, 3.0 M (Na^{+}+H^{+})Cl). 10

Figure S10: Emission spectra of (a) [Eu(*CHX*OCTAPA)]$^{-}$, (b) [Eu(OCTAPA)]$^{-}$). Concentration of 10^{-4} M in distilled water and pH *ca.* 7.1. λ_{exc} = 279 nm....10

Figure S11: Emission spectra of (a) [Tb(*CHX*OCTAPA)]$^{-}$, and (b) [Tb(OCTAPA)]$^{-}$). Concentration of 10^{-4} M in distilled water and pH *ca.* 7.1. λ_{exc} = 279 nm....11

Figure S12: Absorption spectra of (a) [Eu(*CHX*OCTAPA)]$^{-}$, (b) [Eu(OCTAPA)]$^{-}$, (c) [Tb(*CHX*OCTAPA)]$^{-}$, and (d) [Tb(OCTAPA)]$^{-}$. Concentration of 10^{-4} M in distilled water and pH *ca.* 7.1. 11

Figure S13: Lifetimes of the $^5D_0$(Eu) ((a) [Eu(*CHX*OCTAPA)]$^-$, (b) [Eu(OCTAPA)]$^-$) excited states measured in H_2O (blue, left) and D_2O (green, right). Concentration of 10^{-4} M and pH *ca.* 7.1. 12

Figure S14: Lifetimes of the $^5D_4$(Tb) ((a) [Tb(*CHX*OCTAPA)]$^-$, (b) [Tb(OCTAPA)]$^-$) excited states measured in H_2O (blue, left) and D_2O (green, right). Concentration of 10^{-4} M and pH *ca.* 7.1. 12

Figure S15: ^{1}H NMR spectrum of [La(*CHX*OCTAPA)]$^-$ (400 MHz, D2O, pH 7.27, 298 K) 13

Figure S16: ^{13}C NMR spectrum of [La(*CHX*OCTAPA)]$^-$ (400 MHz, D_2O, pH 7.27, 298 K). 14

Figure S17: ^{1}H NMR spectrum of [Yb(*CHX*OCTAPA)]$^-$ (300 MHz, D_2O, pH *ca* 7.0, 298 K) 15

Figure S18: COSY spectrum of [Yb(*CHX*OCTAPA)]$^-$ (300 MHz, D_2O, pH *ca* 7.0, 298 K) 16

Figure S19: ^{1}H NMR spectrum of [Lu(*CHX*OCTAPA)]$^-$ (500 MHz, D_2O, pH *ca* 7.0, 298 K). 17

Figure S20: ^{13}C NMR spectrum of [Lu(*CHX*OCTAPA)]$^-$ (500 MHz, D_2O, pH *ca* 7.0, 298 K). 18

Table S2: Experimental ^{1}H chemical shifts for [Lu(*CHX*OCTAPA)]$^-$ and [Yb(*CHX*OCTAPA)]$^-$ and paramagnetic lanthanide induced shifts (LIS) for the Yb^{3+} complex. 19

Figure S21: Absolute differences (ppm) between the experimental and calculated LIS obtained for the *S,S* and *S,R* isomers of [Yb(*CHX*OCTAPA)]$^-$. 20

Figure S22: Optimized structure for the [Yb(*CHX*OCTAPA)$_{S,R}$(H_2O)]$^-$·2H_2O system, showing the orientation of the magnetic axes. 20

Figure S23: Optimized structures for the (a) [La(*CHX*OCTAPA)$_{S,S}$(H_2O)]$^-$·2H_2O, (b) [La(*CHX*OCTAPA)$_{S,R}$(H_2O)]$^-$ ·2H_2O, (c) [La(OCTAPA)$_{S,S}$(H_2O)]$^-$·2H_2O, and (d) [La(OCTAPA)$_{S,R}$(H_2O)]$^-$·2H_2O systems obtained with DFT calculations (0 Imaginary Frequencies). 21

Figure S24: Optimized structures for the (a) [Pr(*CHX*OCTAPA)$_{S,S}$(H_2O)]$^-$·2H_2O, (b) [Pr(*CHX*OCTAPA)$_{S,R}$(H_2O)]$^-$ ·2H_2O, (c) [Pr(OCTAPA)$_{S,S}$(H_2O)]$^-$·2H_2O, and (d) [Pr(OCTAPA)$_{S,R}$(H_2O)]$^-$·2H_2O systems obtained with DFT calculations (0 Imaginary Frequencies). 22

Figure S25: Optimized structures for the (a) [Gd(*CHX*OCTAPA)$_{S,S}$(H_2O)]$^-$·2H_2O, (b) [Gd(*CHX*OCTAPA)$_{S,R}$(H_2O)]$^-$·2H_2O, (c) [Gd(OCTAPA)$_{S,S}$(H_2O)]$^-$·2H_2O, and (d) [Gd(OCTAPA)$_{S,R}$(H_2O)]$^-$·2H_2O systems obtained with DFT calculations (0 Imaginary Frequencies). 23

Figure S26: Optimized structures for the (a) [Yb(*CHX*OCTAPA)$_{S,S}$(H_2O)]$^-$·2H_2O, (b) [Yb(*CHX*OCTAPA)$_{S,R}$(H_2O)]$^-$ ·2H_2O, (c) [Yb(OCTAPA)$_{S,S}$(H_2O)]$^-$·2H_2O, and (d) [Yb(OCTAPA)$_{S,R}$(H_2O)]$^-$·2H_2O systems obtained with DFT calculations (0 Imaginary Frequencies). 24

Figure S27: Optimized structures for the (a) [Lu(*CHX*OCTAPA)$_{S,S}$(H_2O)]$^-$·2H_2O, (b) [Lu(*CHX*OCTAPA)$_{S,R}$(H_2O)]$^-$ ·2H_2O, (c) [Lu(OCTAPA)$_{S,S}$(H_2O)]$^-$·2H_2O, and (d) [Lu(OCTAPA)$_{S,R}$(H_2O)]$^-$·2H_2O systems obtained with DFT calculations (0 Imaginary Frequencies). 25

Figure S28: Evolution of the calculated distances between the metal and the donor atoms of the ligand. (a) [Ln(*CHX*OCTAPA$_{S,S}$)]$^-$, (b) [Ln(*CHX*OCTAPA$_{S,R}$)]$^-$, (c) [Ln(OCTAPA$_{S,S}$)]$^-$, (d) [Ln(OCTAPA$_{S,R}$)]$^-$ 26

Table S3: Distances / Å between the metal centre and the different donor atoms in the calculated structures. 27

Figure S29: Evolution of the calculated angles / ° (a) N1-M-N4 and (b) O2-M-O3 for the different calculated structures. 27

Figure S30: Potentiometric titration curves simulated for different conditions. 28

Table S4: Selected angles / ° in the calculated structures. 29

Table S5: Optimized Cartesian coordinates (Å) of the [La(*CHX*OCTAPA)$_{S,S}$(H_2O)]$^-$·2H_2O system obtained with DFT calculations (0 Imaginary Frequencies). 30

Table S6: Optimized Cartesian coordinates (Å) of the [La(*CHX*OCTAPA)$_{S,R}$(H_2O)]$^-$·2H_2O system obtained with DFT calculations (0 Imaginary Frequencies). 31

Table S7: Optimized Cartesian coordinates (Å) of the [La(OCTAPA)$_{S,S}$(H_2O)]$^-$·2H_2O system obtained with DFT calculations (0 Imaginary Frequencies). 33

Table S8: Optimized Cartesian coordinates (Å) of the [La(OCTAPA)$_{S,R}$(H_2O)]$^-$·2H_2O system obtained with DFT calculations (0 Imaginary Frequencies). 34

Table S9: Optimized Cartesian coordinates (Å) of the [Pr(*CHX*OCTAPA)$_{S,S}$(H_2O)]$^-$·2H_2O system obtained with DFT calculations (0 Imaginary Frequencies). 36

Table S10: Optimized Cartesian coordinates (Å) of the [Pr(*CHX*OCTAPA)$_{S,R}$(H_2O)]$^-$·2H_2O system obtained with DFT calculations (0 Imaginary Frequencies). 37

Table S11: Optimized Cartesian coordinates (Å) of the [Pr(OCTAPA)$_{S,S}$(H_2O)]$^-$·2H_2O system obtained with DFT calculations (0 Imaginary Frequencies). 39

Table S12: Optimized Cartesian coordinates (Å) of the [Pr(OCTAPA)$_{S,R}$(H_2O)]$^-$·2H_2O system obtained with DFT calculations (0 Imaginary Frequencies). 40

Table S13: Optimized Cartesian coordinates (Å) of the [Gd(*CHX*OCTAPA)$_{S,S}$(H_2O)]$^-$·2H_2O system obtained with DFT calculations (0 Imaginary Frequencies). 42

Table S14: Optimized Cartesian coordinates (Å) of the [Gd(*CHX*OCTAPA)$_{S,R}$(H_2O)]$^-$·2H_2O system obtained with DFT calculations (0 Imaginary Frequencies). 43

Table S15: Optimized Cartesian coordinates (Å) of the [Gd(OCTAPA)$_{S,S}$(H_2O)]$^-$·2H_2O system obtained with DFT calculations (0 Imaginary Frequencies). 45

Table S16: Optimized Cartesian coordinates (Å) of the [Gd(OCTAPA)$_{S,R}$(H_2O)]$^-$·2H_2O system obtained with DFT calculations (0 Imaginary Frequencies). 47

Table S17: Optimized Cartesian coordinates (Å) of the [Yb(*CHX*OCTAPA)$_{S,S}$(H_2O)]$^-$·2H_2O system obtained with DFT calculations (0 Imaginary Frequencies). 48

Table S18: Optimized Cartesian coordinates (Å) of the [Yb(*CHX*OCTAPA)$_{S,R}$(H_2O)]$^-$·2H_2O system obtained with DFT calculations (0 Imaginary Frequencies). 50

Table S19: Optimized Cartesian coordinates (Å) of the [Yb(OCTAPA)$_{S,S}$(H_2O)]$^-$·2H_2O system obtained with DFT calculations (0 Imaginary Frequencies). 51

Table S20: Optimized Cartesian coordinates (Å) of the [Yb(OCTAPA)$_{S,R}$(H_2O)]$^-$·2H_2O system obtained with DFT calculations (0 Imaginary Frequencies). 53

Table S21: Optimized Cartesian coordinates (Å) of the [Lu(*CHX*OCTAPA)$_{S,S}$(H_2O)]$^-$·2H_2O system obtained with DFT calculations (0 Imaginary Frequencies). 54

Table S22: Optimized Cartesian coordinates (Å) of the [Lu(*CHX*OCTAPA)$_{S,R}$(H_2O)]$^-$·2H_2O system obtained with DFT calculations (0 Imaginary Frequencies). 56

Table S23: Optimized Cartesian coordinates (Å) of the [Lu(OCTAPA)$_{S,S}$(H_2O)]$^-$·2H_2O system obtained with DFT calculations (0 Imaginary Frequencies). 57

Table S24: Optimized Cartesian coordinates (Å) of the [Lu(OCTAPA)$_{S,R}$(H_2O)]$^-$·2H_2O system obtained with DFT calculations (0 Imaginary Frequencies). 59

References....60

Table S1. Comparison of the protonation constants of *CHX*OCTAPA^{4-} determined in this work with those reported in the literature (25 °C, 0.15 M NaCl).

	*CHX*OCTAPA$^{4-\,a}$	*CHX*OCTAPA$^{4-\,b}$	*CHX*OCTAPA$^{4-\,c}$
**logK_1^H**	**9.52(1)**	9.35	9.23
**logK_2^H**	**5.51(1)**	5.66	5.40
**logK_3^H**	**3.99(1)**	4.20	3.94
**logK_4^H**	**3.43(1)**	3.72	2.24
**logK_5^H**	**1.59(1)**	2.62	1.82
**logK_6^H**	**0.61(4)**		1.91
**log β_{014}**	**22.44**	**22.93[a]**	**20.81[b]**
**log β_{015}**	**24.03**	**25.56[a]**	**22.63[b]**

a This work. b Data from Ref. [1]; *c* Data from Ref. [2];

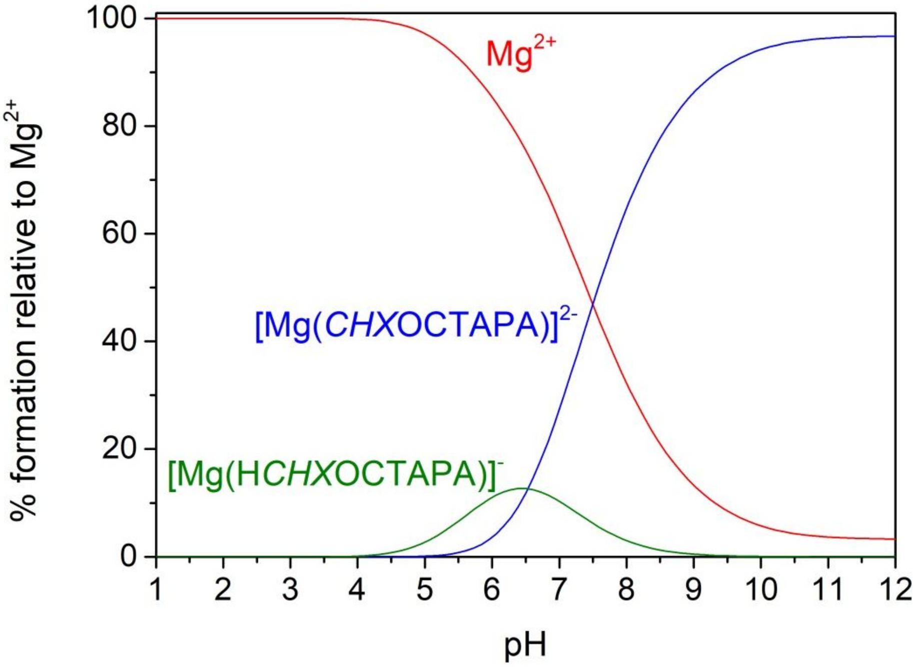

Figure S1: Species distribution curves calculated for the Mg^{2+}:*CHX*OCTAPA^{4-} system (c_{Lig}=c_{Mg2+}=10^{-3} M).

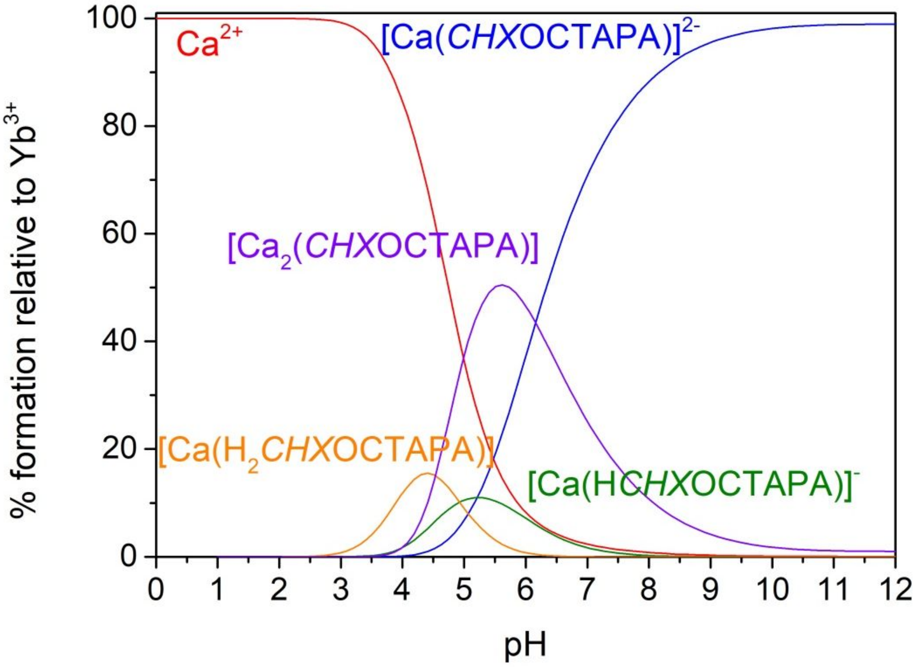

Figure S2: Species distribution curves calculated for the Ca^{2+}:*CHX*OCTAPA^{4-} system (c_{Lig}=c_{Ca2+}=10^{-3} M).

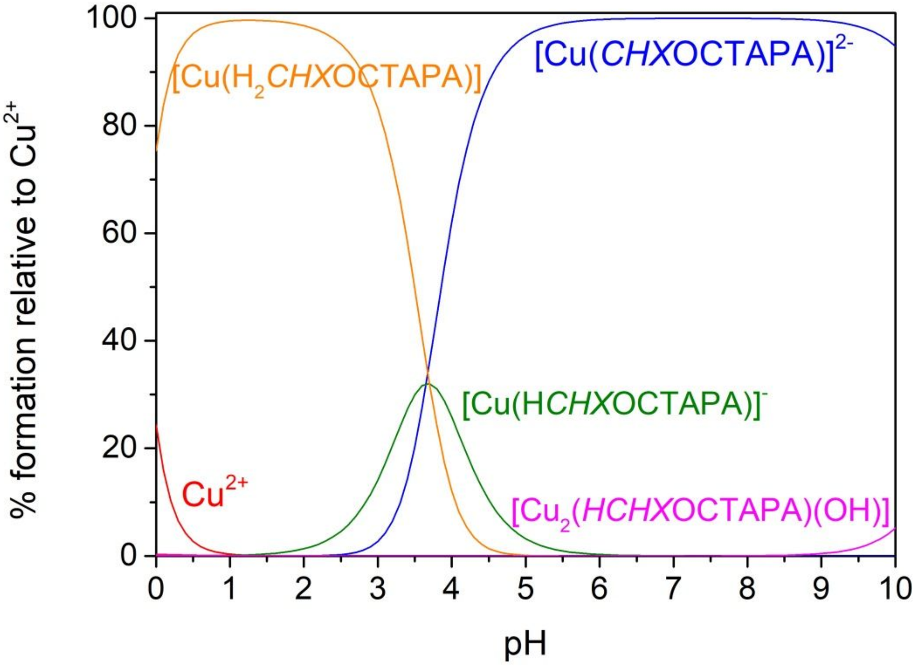

Figure S3: Species distribution curves calculated for the Cu^{2+}:*CHX*OCTAPA^{4-} system (c_{Lig}=c_{Cu2+}=10^{-3} M).

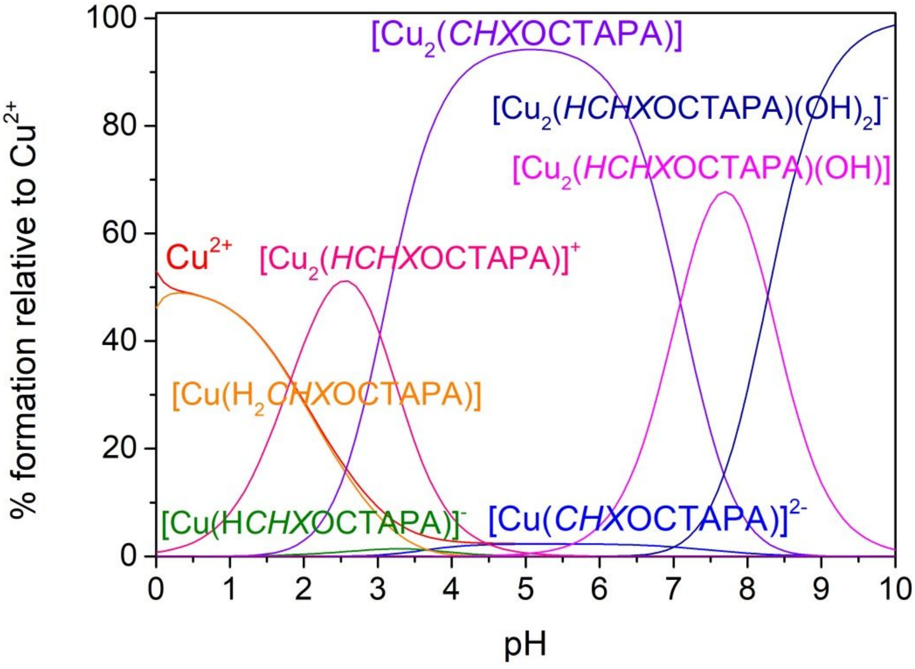

Figure S4: Species distribution curves calculated for the Cu^{2+}:*CHX*OCTAPA^{4-} system (c_{Lig}=10^{-3} M, c_{Cu2+}=2 x 10^{-3} M).

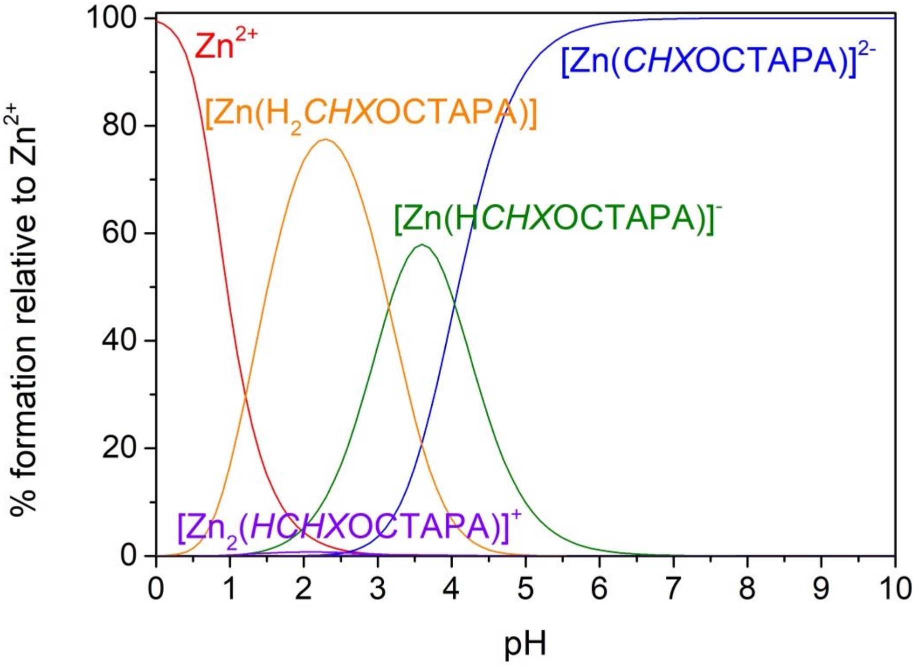

Figure S5: Species distribution curves calculated for the Zn^{2+}:*CHX*OCTAPA^{4-} system (c_{Lig}=c_{Zn2+}=10^{-3} M).

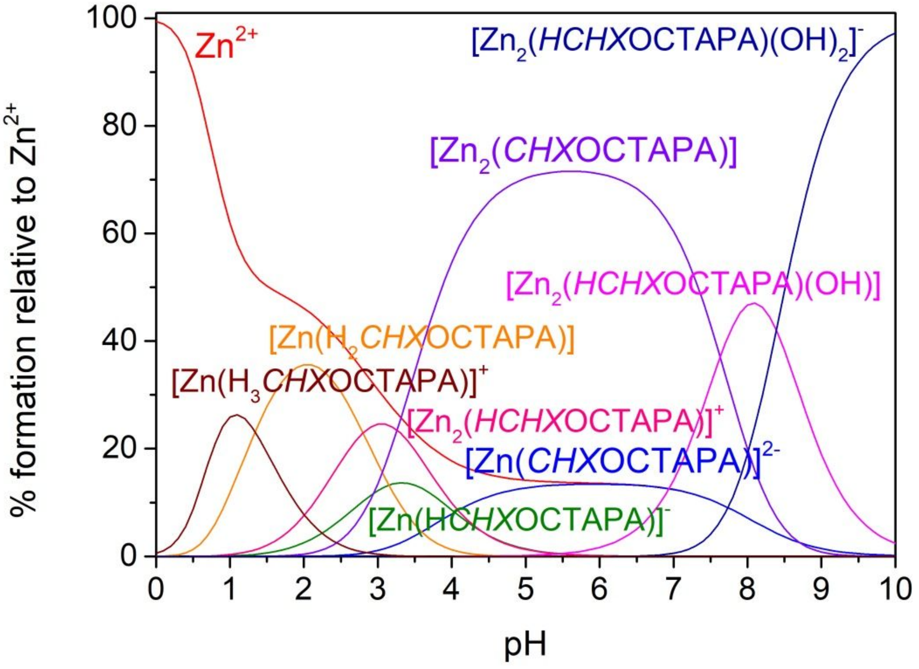

Figure S6: Species distribution curves calculated for the Zn^{2+}:*CHX*OCTAPA^{4-} system (c_{Lig}=10^{-3} M, c_{Zn2+}=2 x 10^{-3} M).

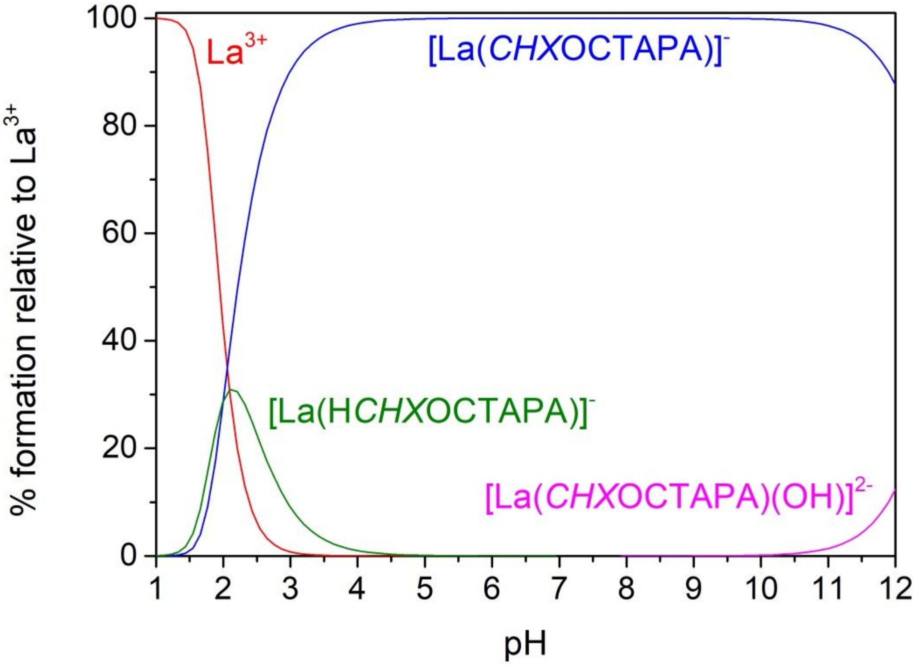

Figure S7: Species distribution curves calculated for the La^{3+}:*CHX*OCTAPA^{4-} system (c_{Lig}=c_{La3+}=10^{-3} M).

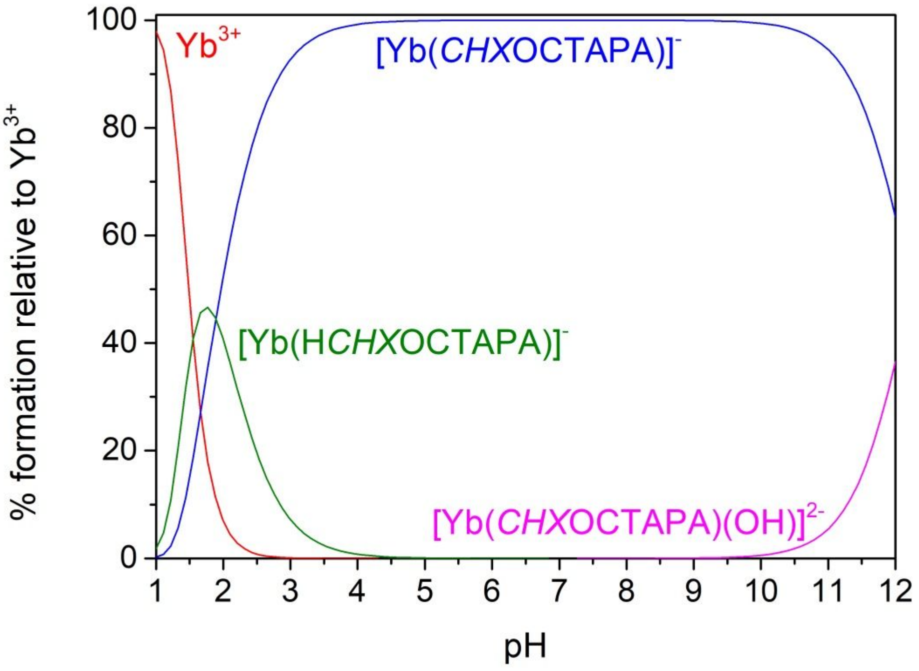

Figure S8: Species distribution curves calculated for the Yb^{3+}:*CHX*OCTAPA^{4-} system (c_{Lig}=c_{Yb3+}=10^{-3} M).

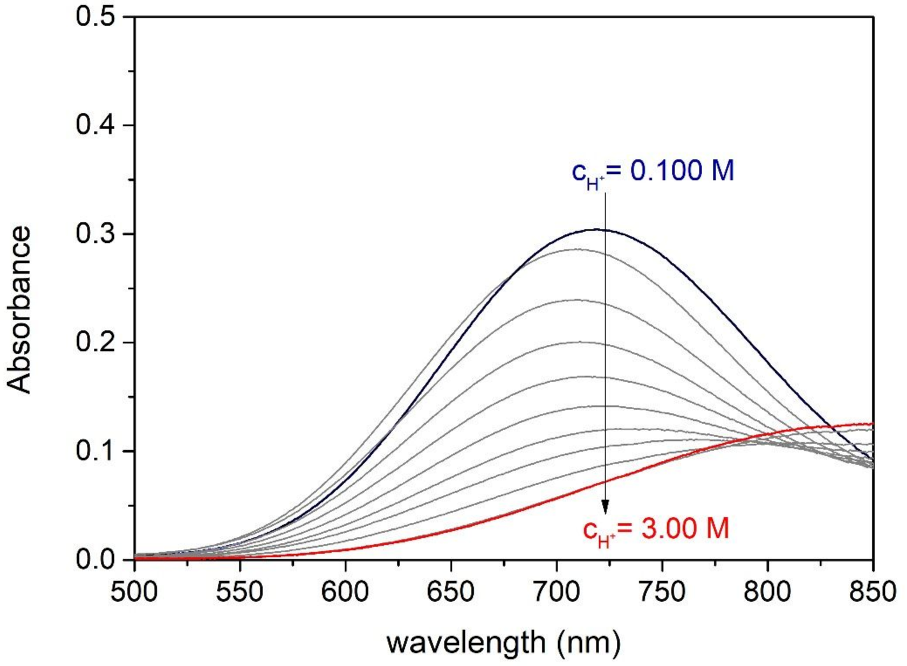

Figure S9: Changes observed in the absorption spectrum of the Cu^{2+}:*CHX*OCTAPA^{4-} system (c_{Lig}= 3.110 mM, c_{Cu2+}= 3.065 mM, 25 ºC, 3.0 M (Na^++H^+)Cl).

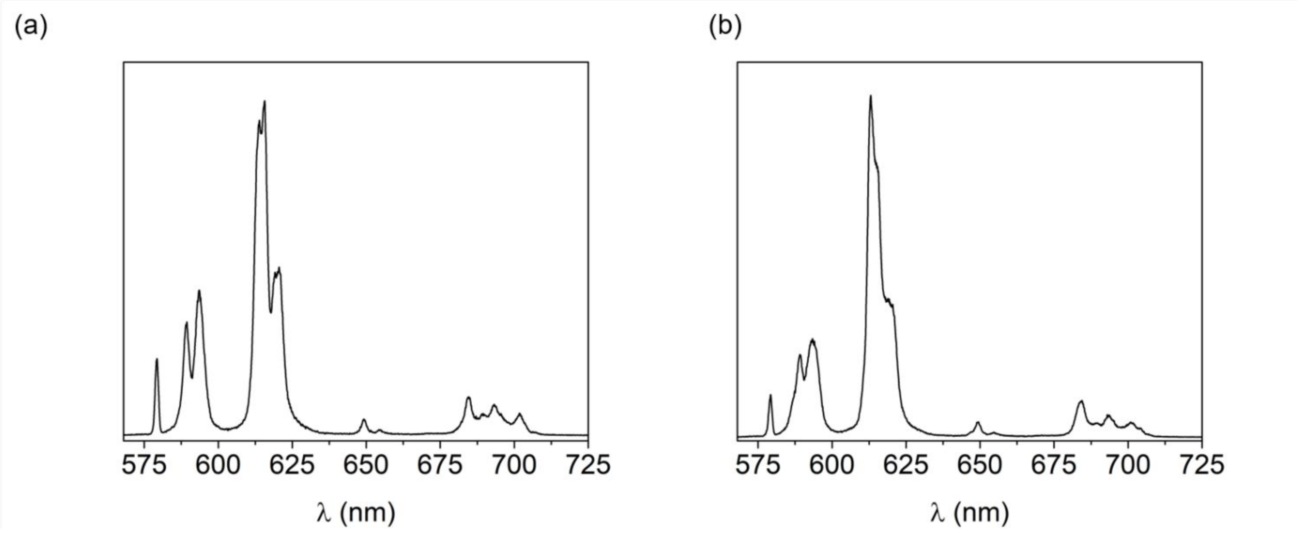

Figure S10: Emission spectra of (a) [Eu(*CHX*OCTAPA)]$^-$, (b) [Eu(OCTAPA)]$^-$). Concentration of 10^{-4} M in distilled water and pH *ca.* 7.1. λ_{exc} = 279 nm.

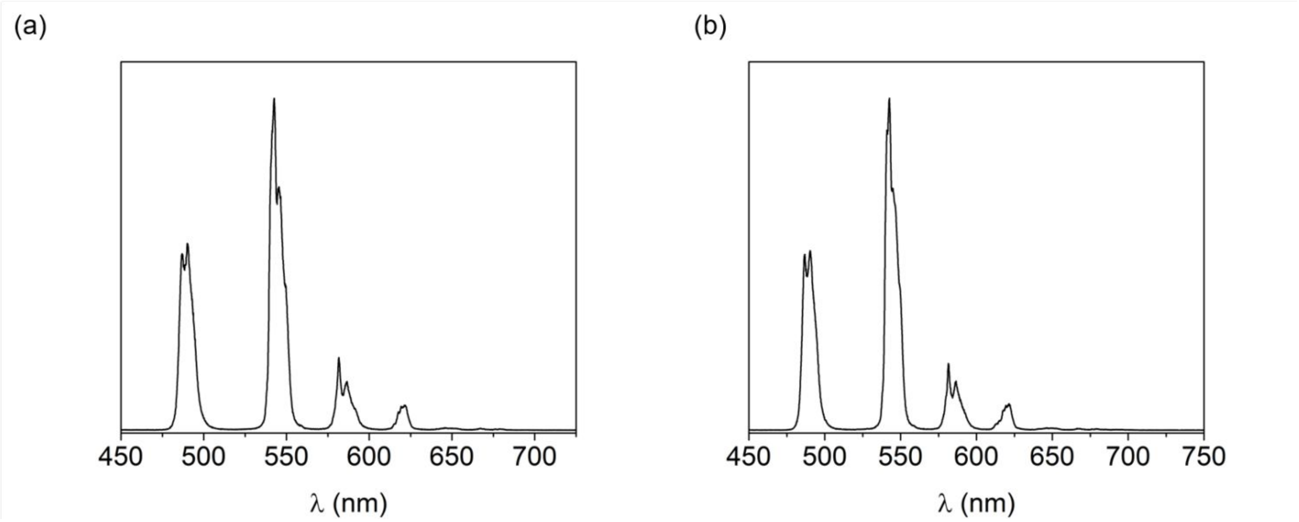

Figure S11: Emission spectra of (a) [Tb(*CHX*OCTAPA)]$^{-}$, and (b) [Tb(OCTAPA)]$^{-}$). Concentration of 10^{-4} M in distilled water and pH *ca.* 7.1. λ_{exc} = 279 nm.

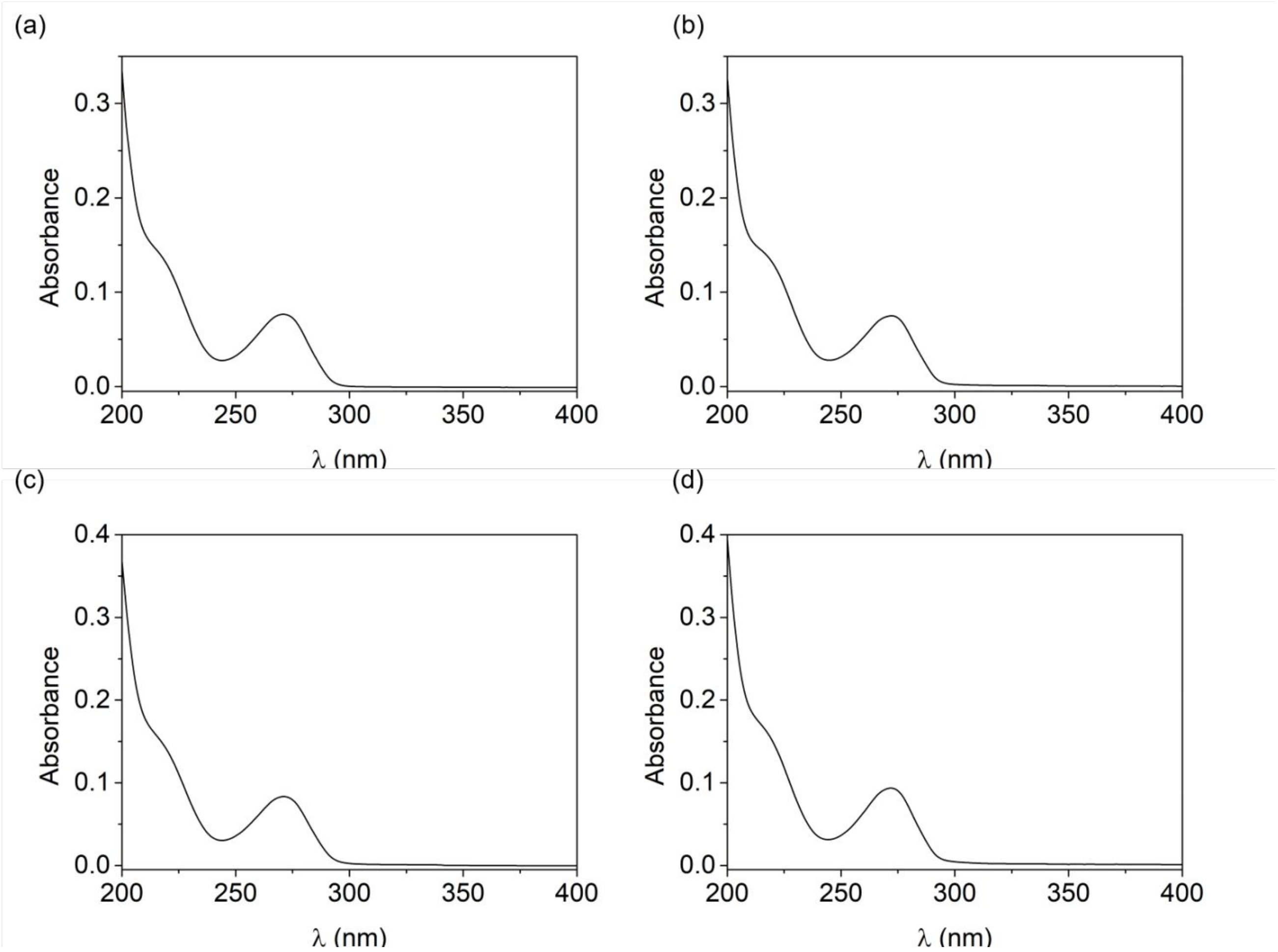

Figure S12: Absorption spectra of (a) [Eu(*CHX*OCTAPA)]$^{-}$, (b) [Eu(OCTAPA)]$^{-}$, (c) [Tb(*CHX*OCTAPA)]$^{-}$, and (d) [Tb(OCTAPA)]$^{-}$. Concentration of 10^{-4} M in distilled water and pH *ca.* 7.1.

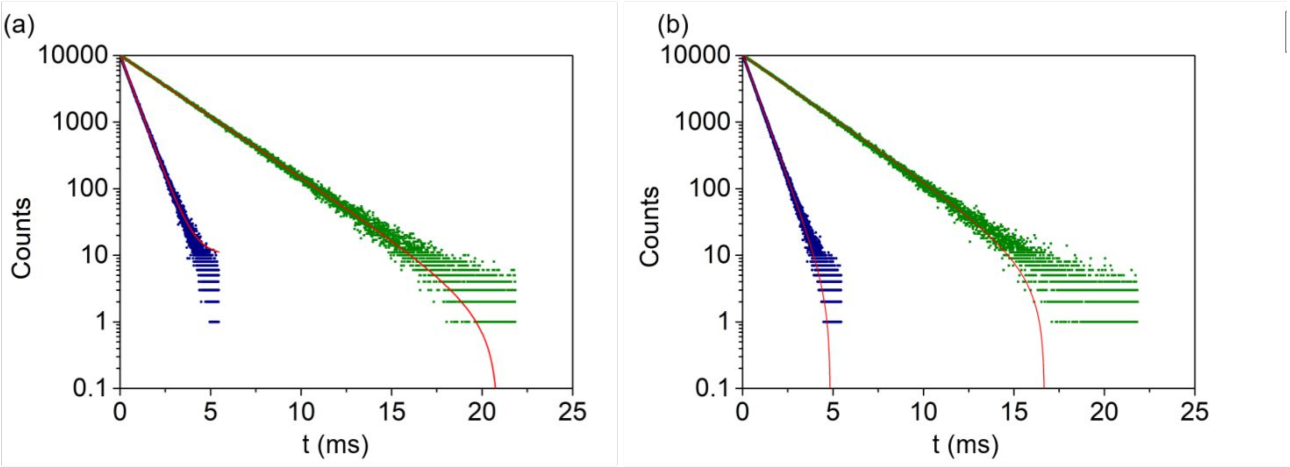

Figure S13: Lifetimes of the $^5D_0$(Eu) ((a) [Eu(*CHX*OCTAPA)]$^-$, (b) [Eu(OCTAPA)]$^-$) excited states measured in H_2O (blue, left) and D_2O (green, right). Concentration of 10^{-4} M and pH *ca.* 7.1.

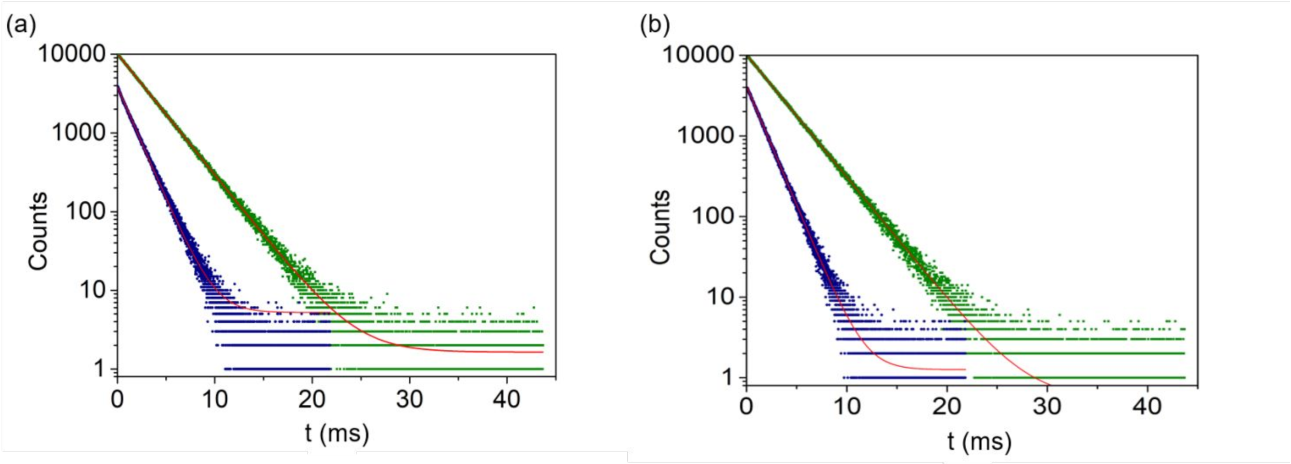

Figure S14: Lifetimes of the $^5D_4$(Tb) ((a) [Tb(*CHX*OCTAPA)]$^-$, (b) [Tb(OCTAPA)]$^-$) excited states measured in H_2O (blue, left) and D_2O (green, right). Concentration of 10^{-4} M and pH *ca.* 7.1.

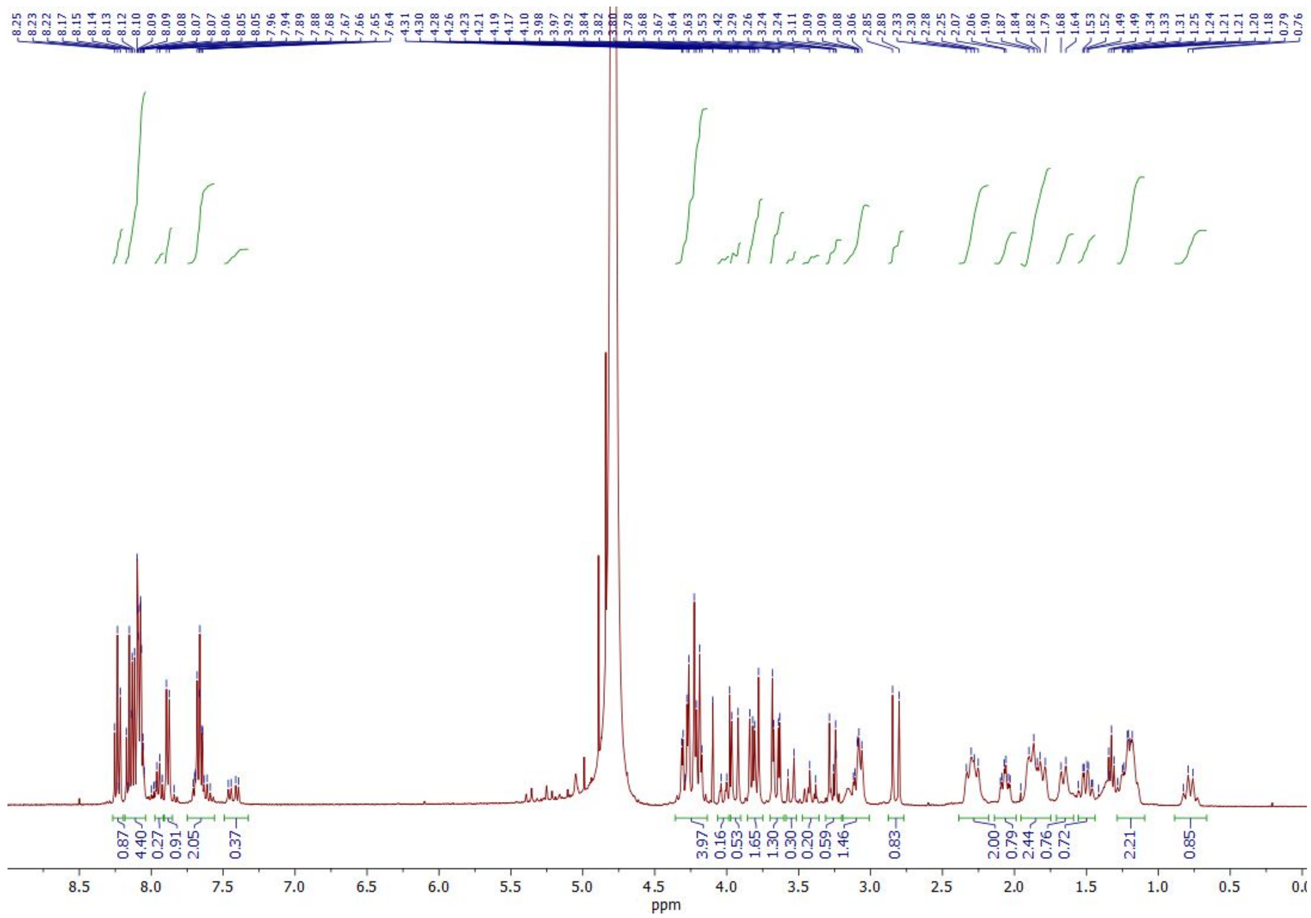

Figure S15: ^{1}H NMR spectrum of [La(*CHX*OCTAPA)]$^{-}$ (400 MHz, D2O, pH 7.27, 298 K)

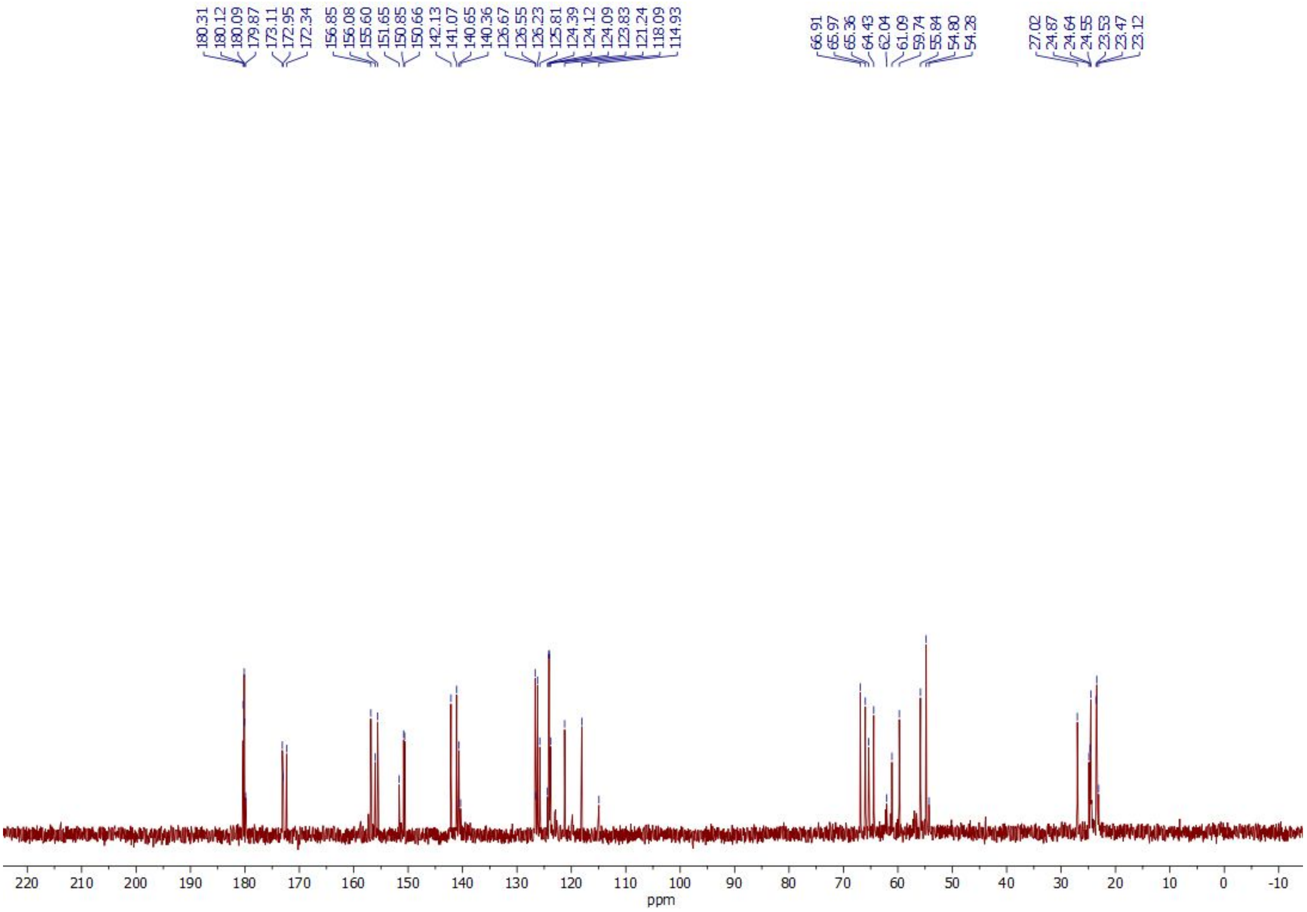

Figure S16: ^{13}C NMR spectrum of [La(*CHX*OCTAPA)]$^{-}$ (400 MHz, D_2O, pH 7.27, 298 K).

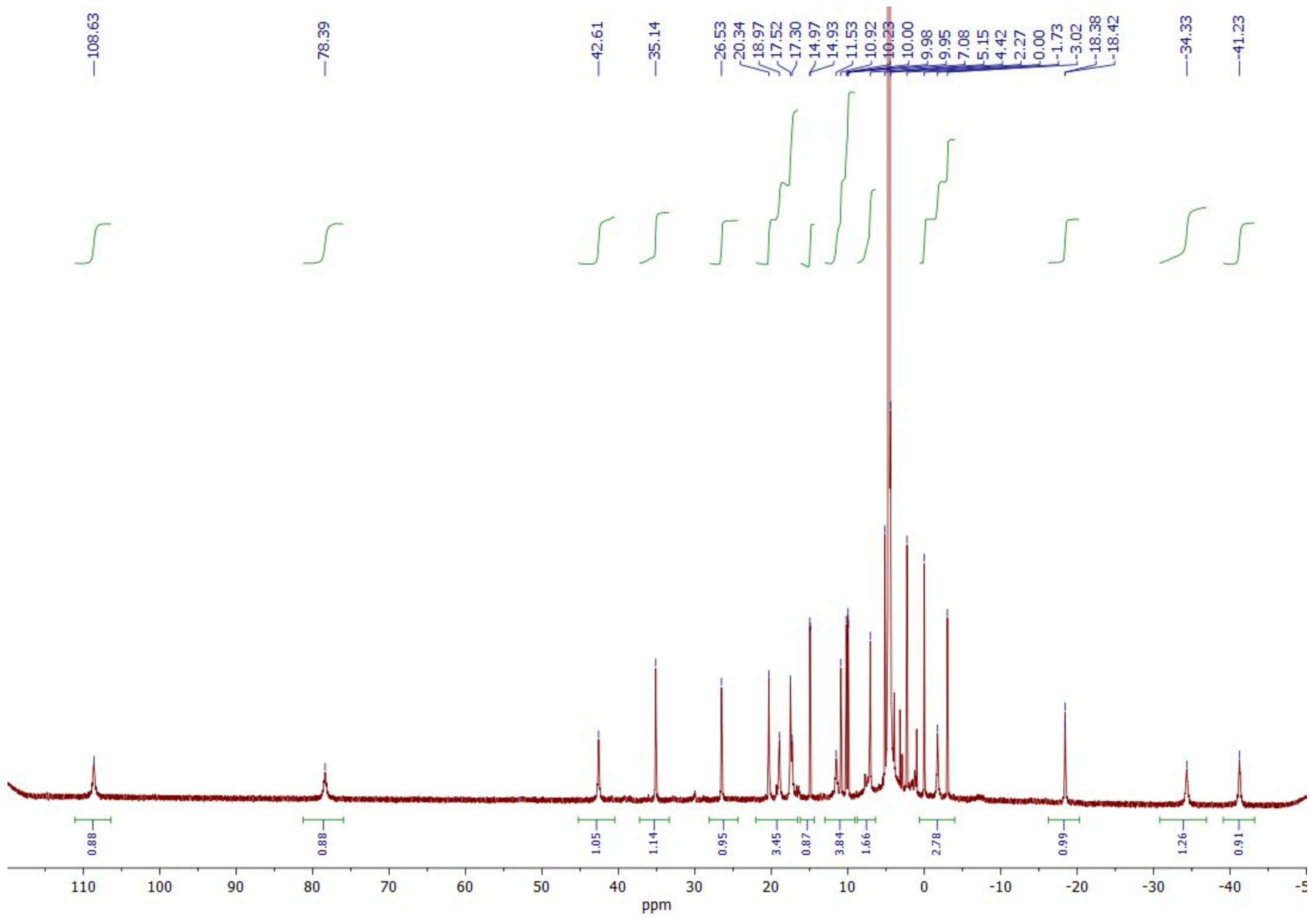

Figure S17: ^{1}H NMR spectrum of $[Yb(\textit{CHX}OCTAPA)]^{-}$ (300 MHz, D_2O, pH *ca* 7.0, 298 K)

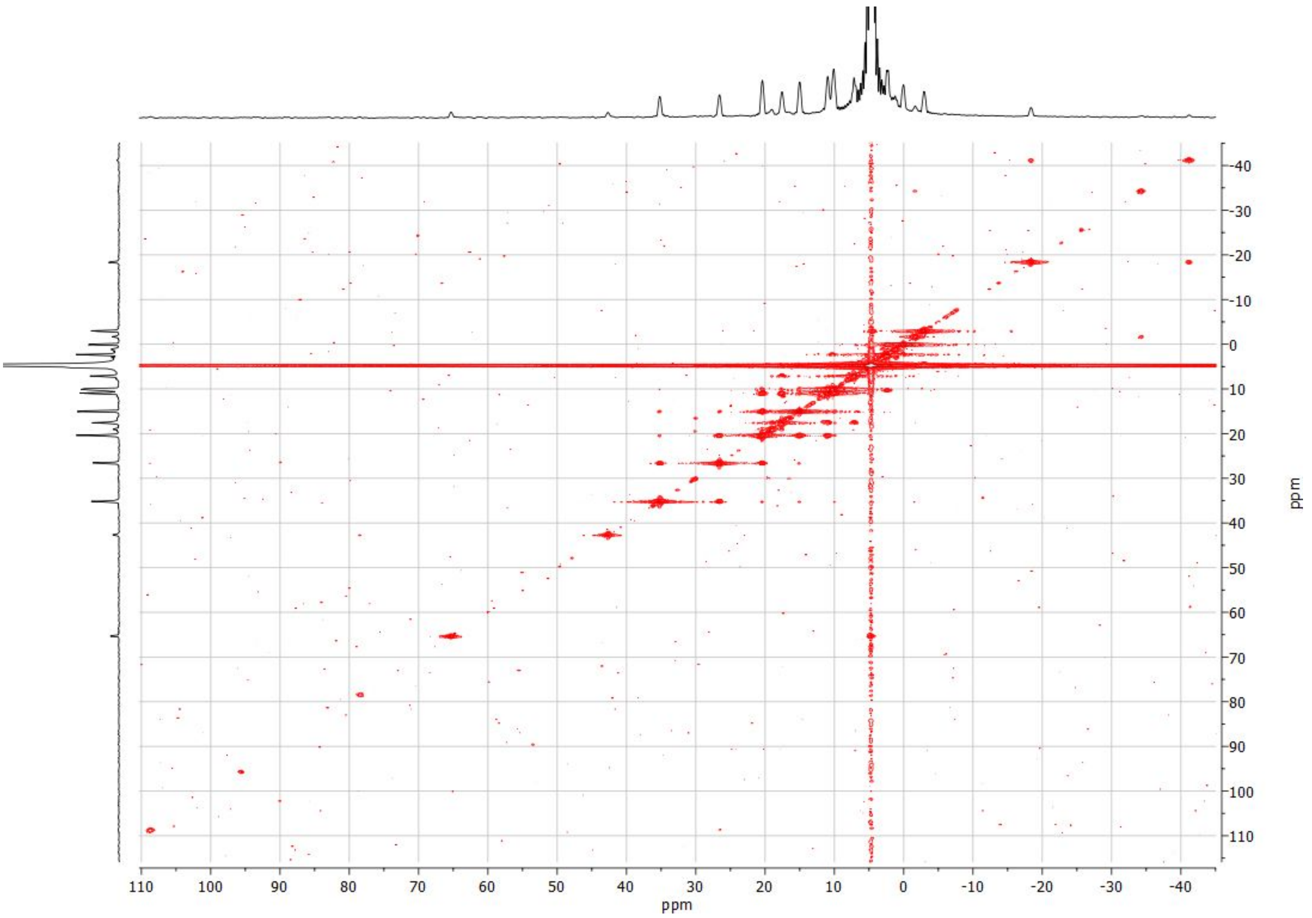

Figure S18: COSY spectrum of [Yb(*CHX*OCTAPA)]$^{-}$ (300 MHz, D_2O, pH *ca* 7.0, 298 K)

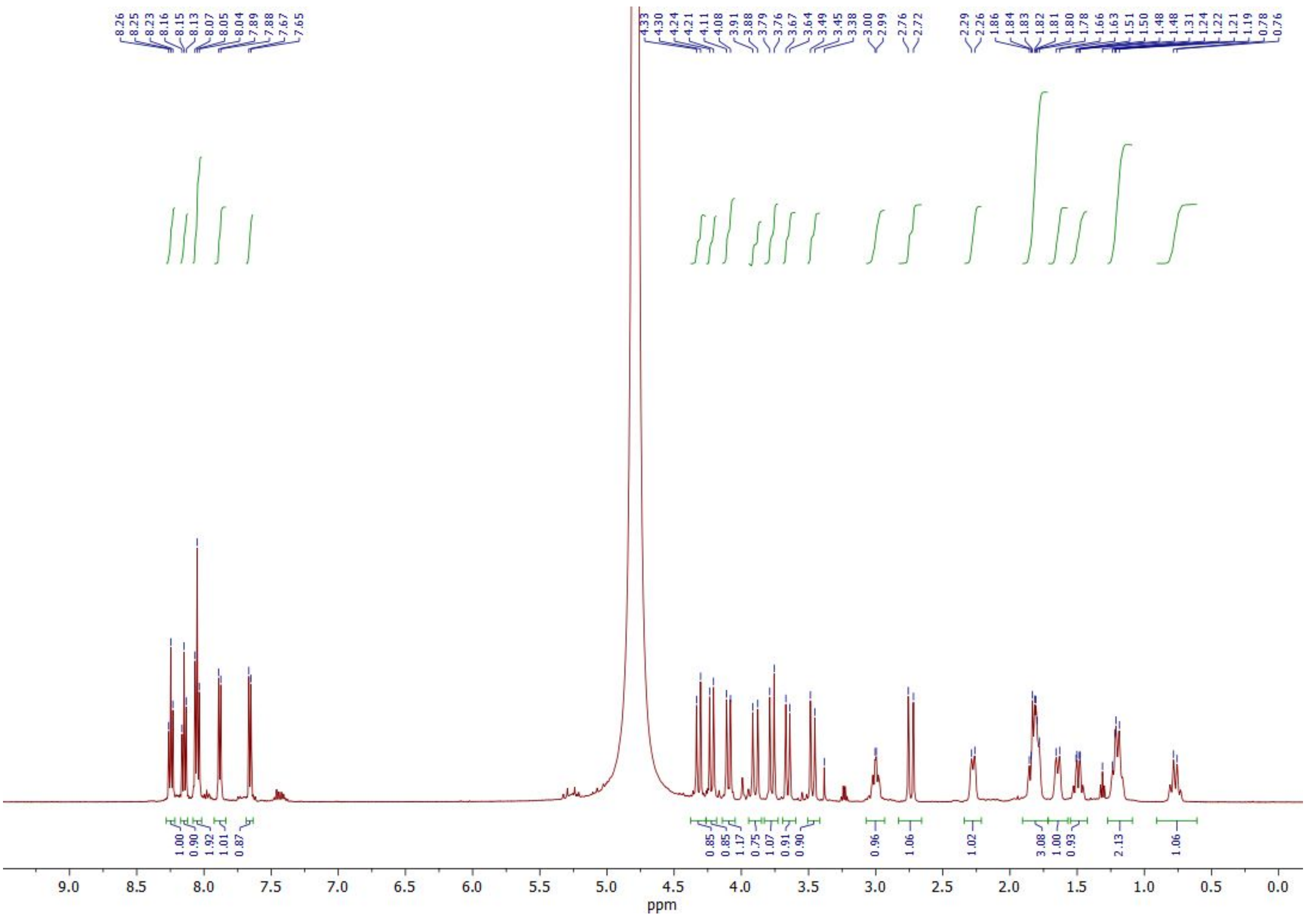

Figure S19: ^{1}H NMR spectrum of $[Lu(\mathit{CHX}OCTAPA)]^{-}$ (500 MHz, D_2O, pH *ca* 7.0, 298 K).

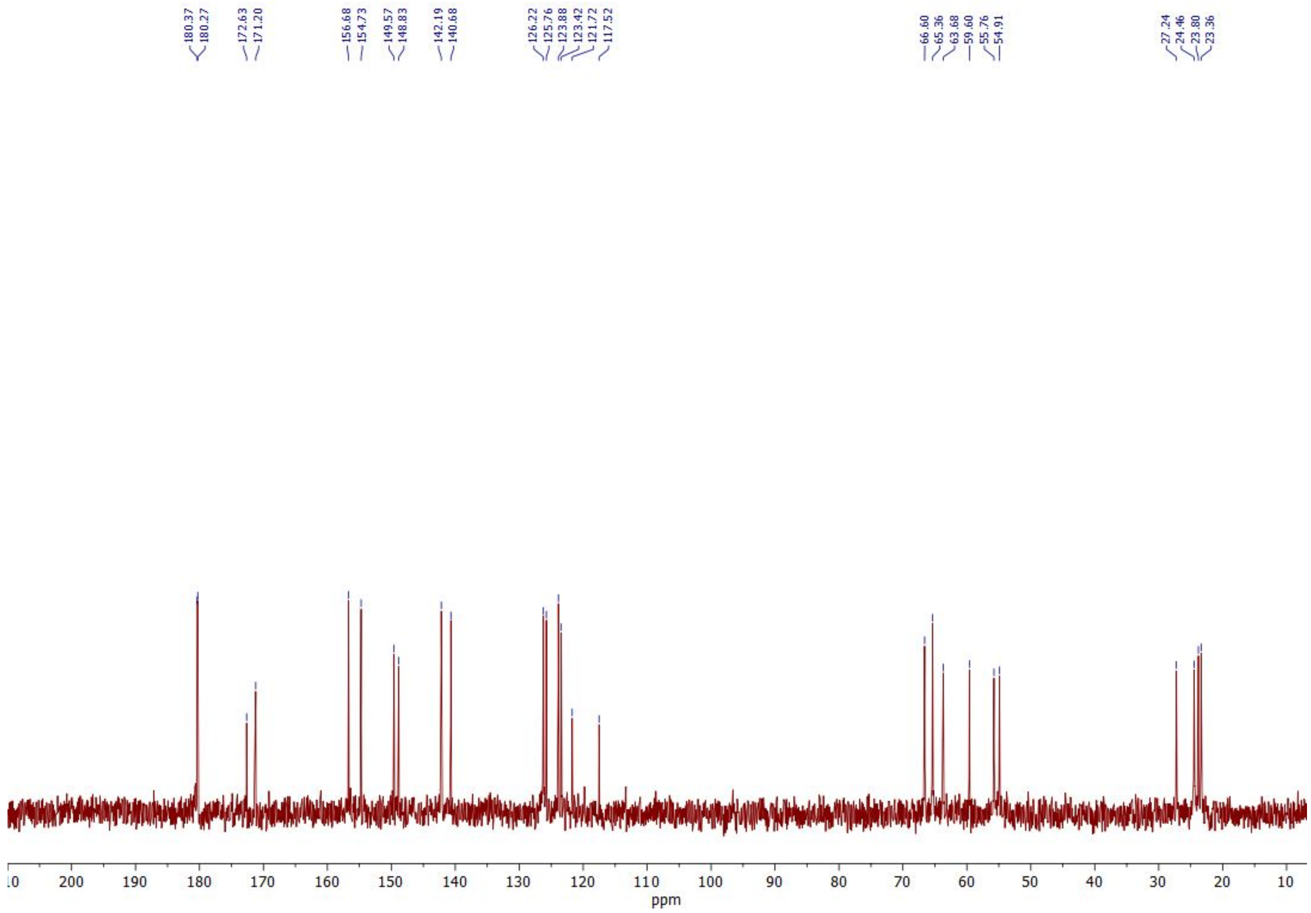

Figure S20: ^{13}C NMR spectrum of [Lu(*CHX*OCTAPA)]$^{-}$ (500 MHz, D_2O, pH *ca* 7.0, 298 K).

Table S2: Experimental ^{1}H chemical shifts for $[Lu(\textit{CHX}OCTAPA)]^{-}$ and $[Yb(\textit{CHX}OCTAPA)]^{-}$ and paramagnetic lanthanide induced shifts (LIS) for the Yb^{3+} complex.

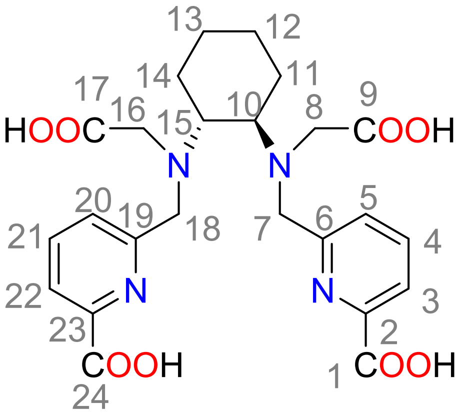

H_4*CHX*OCTAPA

	Lu / ppm	Yb / ppm	LIS /Yb	S,R-ISOMER		S,S-ISOMER	
				LIScalc/Yb	Abs_Diff	LIScalc/Yb	Abs_Diff
**H3**	7.95	-3.02	10.97	12.795	1.825	12.51	1.54
**H4**	8.13	4.42	3.71	4.9803	1.2703	13.11	9.4
**H5**	7.77	5.15	2.62	4.0876	1.4676	19.14	16.52
**H7AX**	3.99	-34.33	38.32	37.705	0.615	4.08	34.24
**H7EQ**	4.21	-1.73	5.94	7.9979	2.0579	18.94	13
**H8AX**	3.36	-41.23	44.59	45.723	1.133	37.76	6.83
**H8EQ**	3.66	-18.42	22.08	21.452	0.628	19.84	2.24
**H10**	2.89	18.97	-16.08	-14.357	1.723	-16	0.08
**H11AX**	1.38	17.52	-16.14	-14.113	2.027	-9.09	7.05
**H11EQ**	2.16	7.08	-4.92	-3.7565	1.1635	-0.18	4.74
**H12AX**	1.05	10.92	-9.87	-9.7558	0.1142	-8.25	1.62
**H12EQ**	1.68	9.96	-8.28	-7.8256	0.4544	-6.22	2.06
**H13AX**	1.09	20.34	-19.25	-18.807	0.443	-17.28	1.97
**H13EQ**	1.68	14.95	-13.27	-13.282	0.012	-12.83	0.44
**H14AX**	0.66	35.14	-34.48	-34.131	0.349	-35.06	0.58
**H14EQ**	1.53	26.53	-25	-26.075	1.075	-27.18	2.18
**H15**	1.73	108.63	-106.9	-107.66	0.76	-84.24	22.66
**H16AX**	2.63	11.53	-8.9	-4.7305	4.1695	-12.33	3.43
**H16EQ**	3.79	17.3	-13.51	-17.316	3.806	-31.58	18.07
**H18AX**	3.55	78.39	-74.84	-72.592	2.248	-66.89	7.95
**18EQ**	4.12	42.61	-38.49	-39.084	0.594	-60.43	21.94
**H20**	7.55	10.23	-2.68	-3.869	1.189	2.99	5.67
**H21**	8.04	2.27	5.77	4.6962	1.0738	10.96	5.19
**H22**	7.94	0	7.94	7.8314	0.1086	19.24	11.3

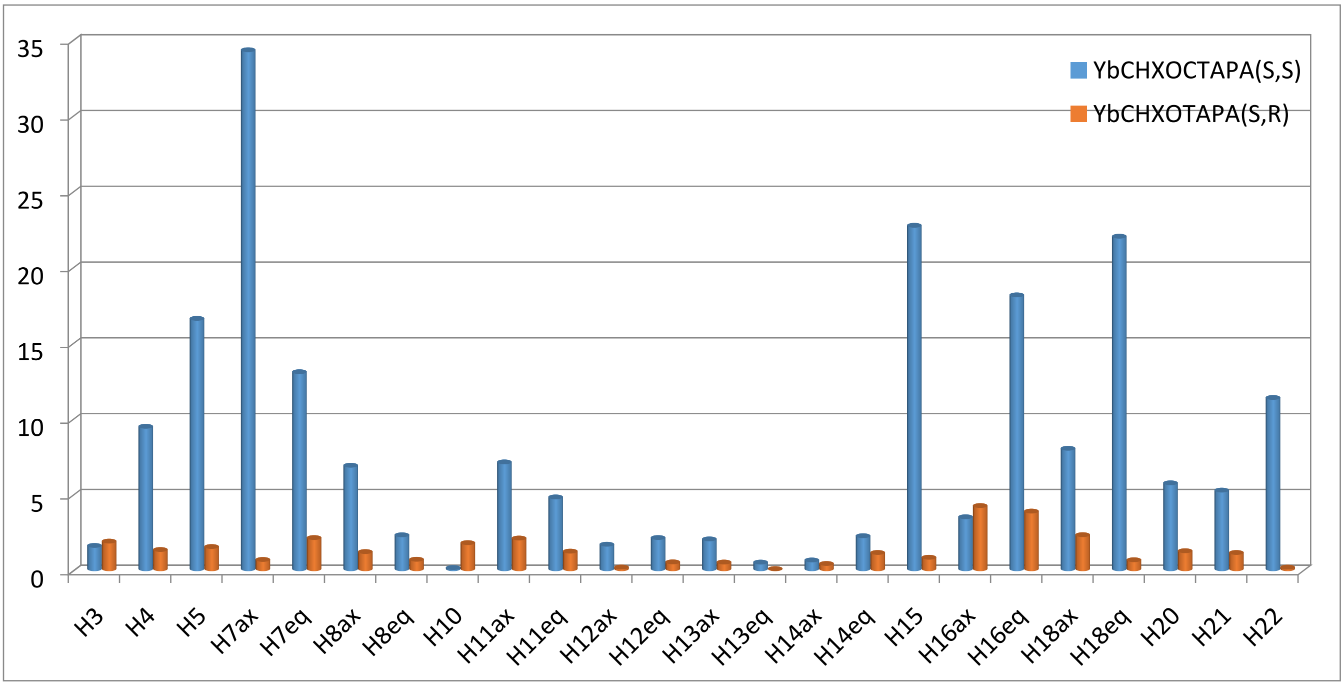

Figure S21: Absolute differences (ppm) between the experimental and calculated LIS obtained for the *S,S* and *S,R* isomers of [Yb(*CHX*OCTAPA)]$^-$.

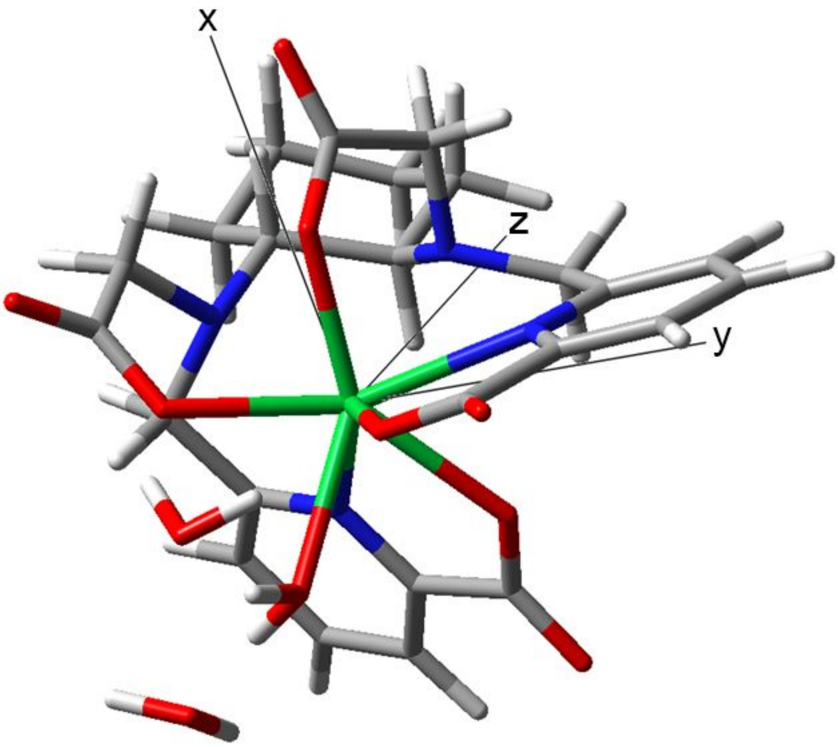

Figure S22: Optimized structure for the [Yb(*CHX*OCTAPA)$_{S,R}$(H_2O)]$^-$·2H_2O system, showing the orientation of the magnetic axes.

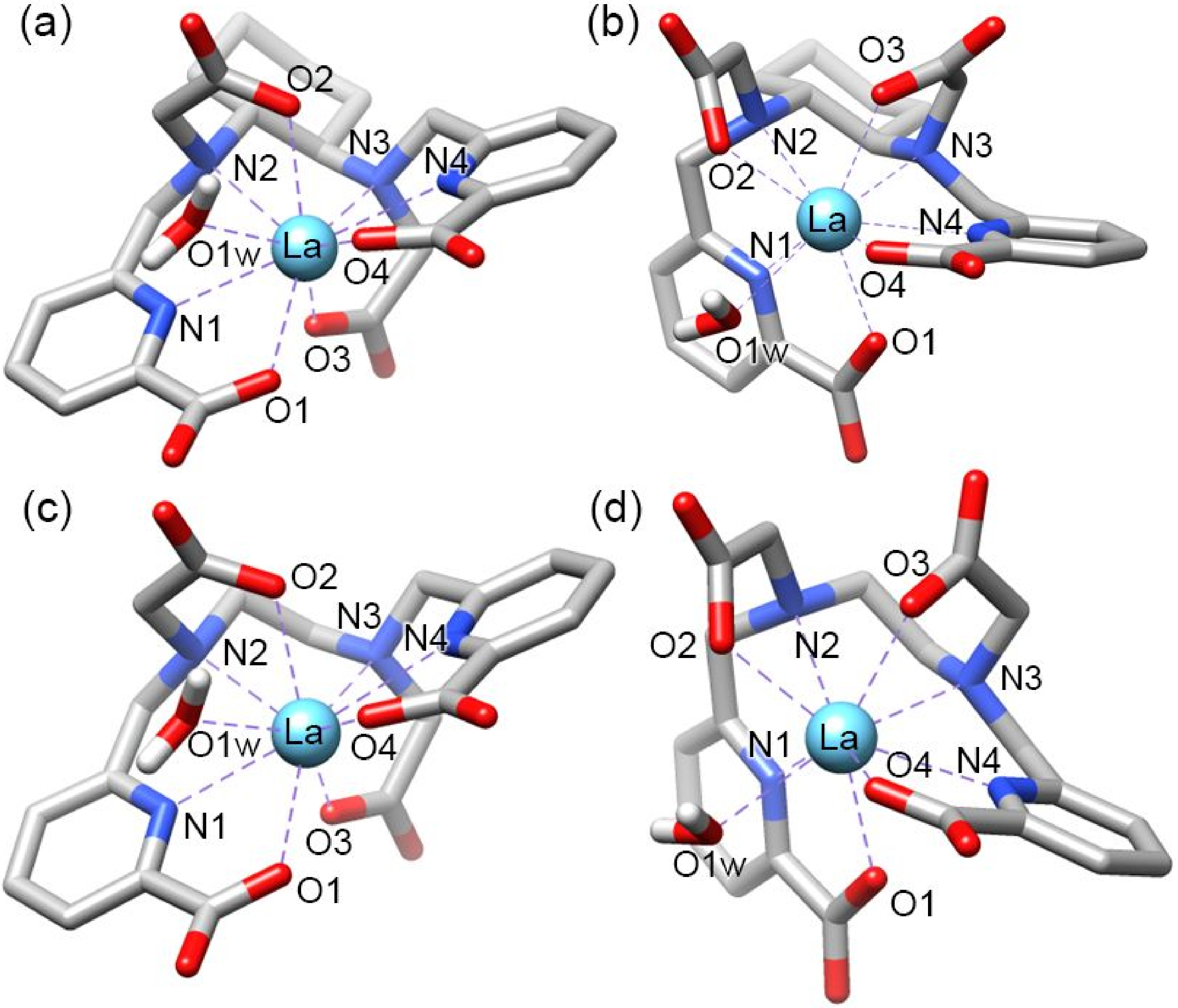

Figure S23: Optimized structures for the (a) [La(*CHX*OCTAPA)$_{S,S}$(H_2O)]$^-$·2H_2O, (b) [La(*CHX*OCTAPA)$_{S,R}$(H_2O)]$^-$·2H_2O, (c) [La(OCTAPA)$_{S,S}$(H_2O)]$^-$·2H_2O, and (d) [La(OCTAPA)$_{S,R}$(H_2O)]$^-$·2H_2O systems obtained with DFT calculations (0 Imaginary Frequencies).

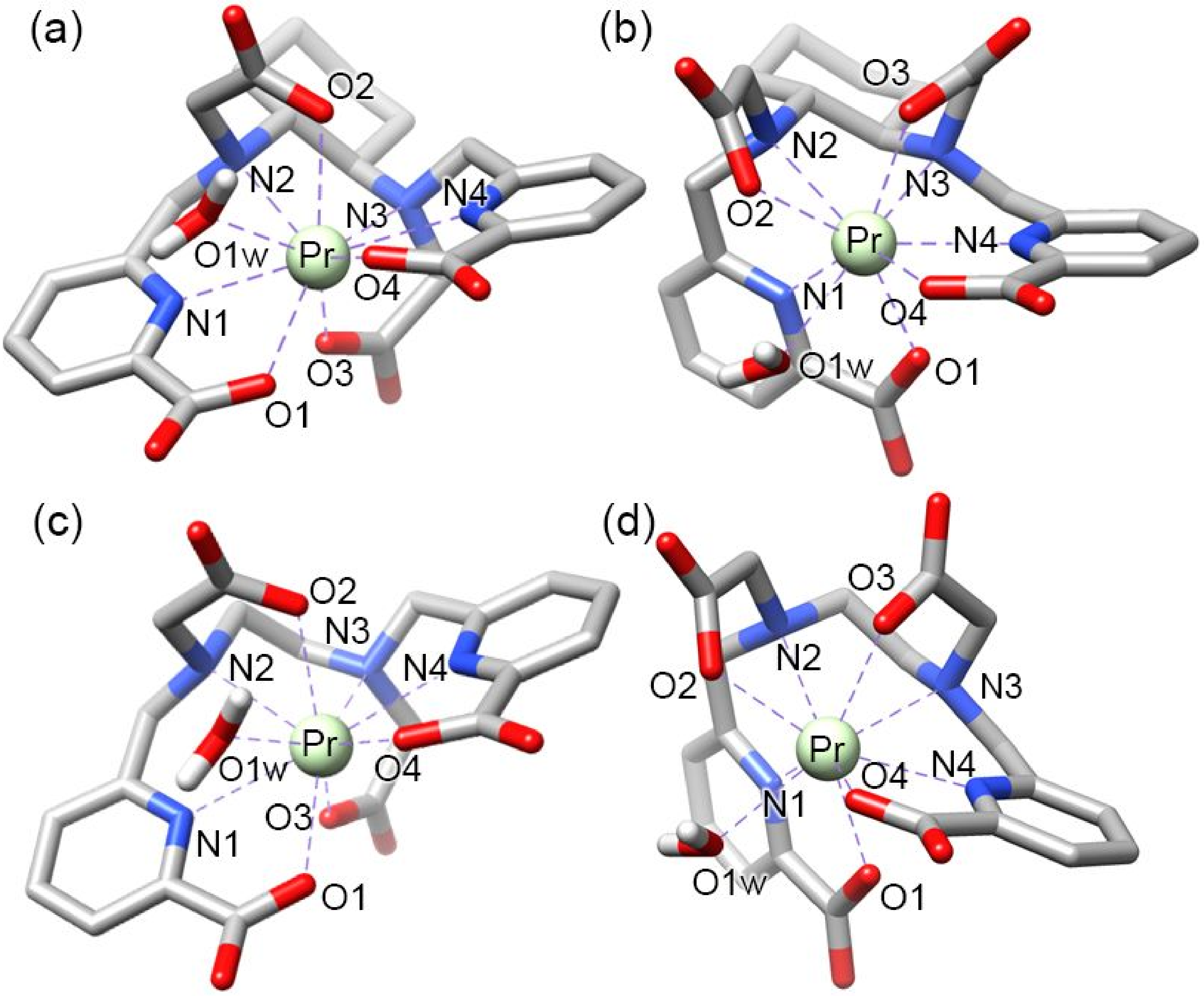

Figure S24: Optimized structures for the (a) $[Pr(\textit{CHX}OCTAPA)_{S,S}(H_2O)]^{-}\cdot 2H_2O$, (b) $[Pr(\textit{CHX}OCTAPA)_{S,R}(H_2O)]^{-}\cdot 2H_2O$, (c) $[Pr(OCTAPA)_{S,S}(H_2O)]^{-}\cdot 2H_2O$, and (d) $[Pr(OCTAPA)_{S,R}(H_2O)]^{-}\cdot 2H_2O$ systems obtained with DFT calculations (0 Imaginary Frequencies).

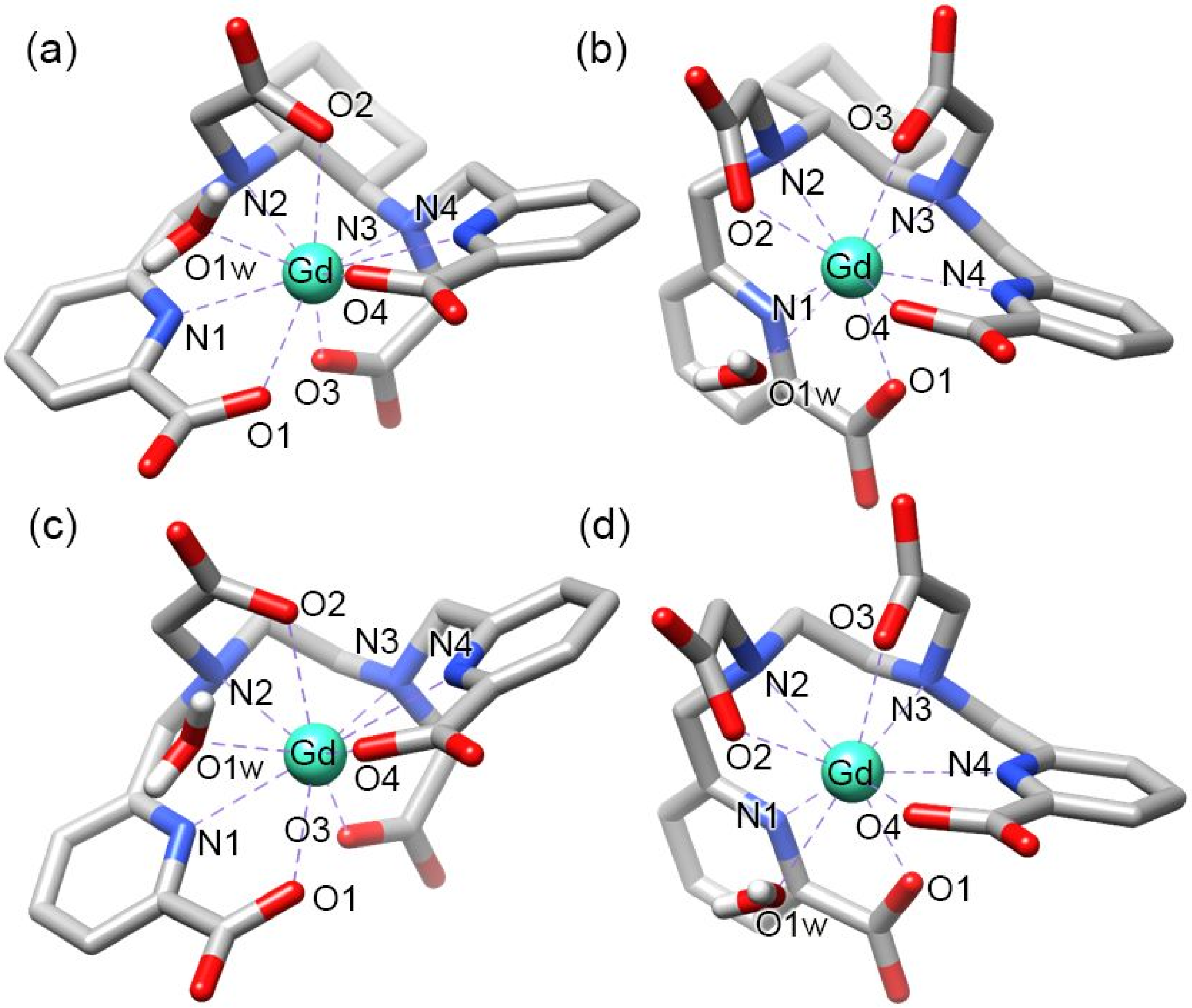

Figure S25: Optimized structures for the (a) [Gd(*CHX*OCTAPA)$_{S,S}$(H_2O)]$^-$·2H_2O, (b) [Gd(*CHX*OCTAPA)$_{S,R}$(H_2O)]$^-$·2H_2O, (c) [Gd(OCTAPA)$_{S,S}$(H_2O)]$^-$·2H_2O, and (d) [Gd(OCTAPA)$_{S,R}$(H_2O)]$^-$·2H_2O systems obtained with DFT calculations (0 Imaginary Frequencies).

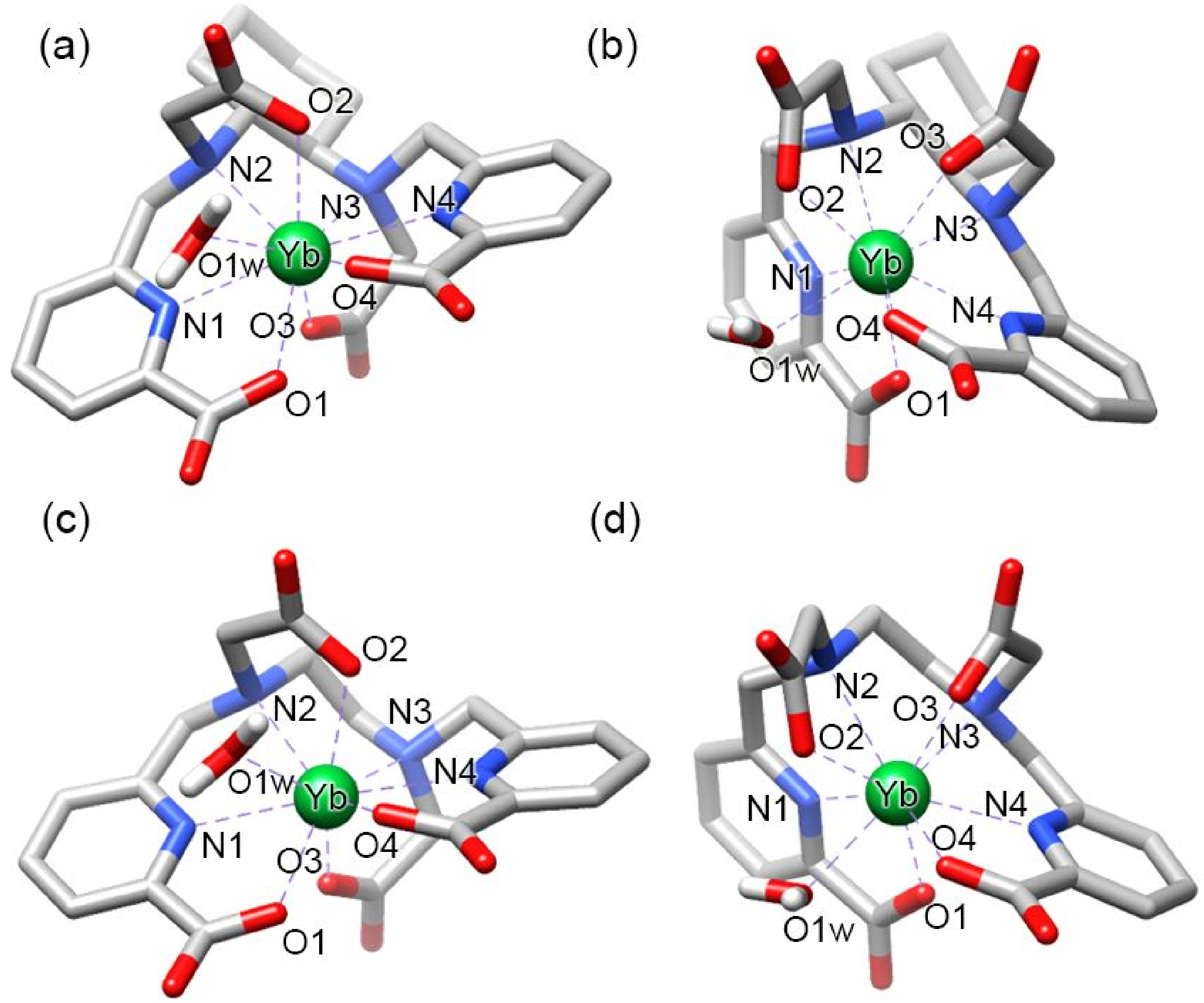

Figure S26: Optimized structures for the (a) $[Yb(\textit{CHX}OCTAPA)_{S,S}(H_2O)]^-\cdot 2H_2O$, (b) $[Yb(\textit{CHX}OCTAPA)_{S,R}(H_2O)]^-\cdot 2H_2O$, (c) $[Yb(OCTAPA)_{S,S}(H_2O)]^-\cdot 2H_2O$, and (d) $[Yb(OCTAPA)_{S,R}(H_2O)]^-\cdot 2H_2O$ systems obtained with DFT calculations (0 Imaginary Frequencies).

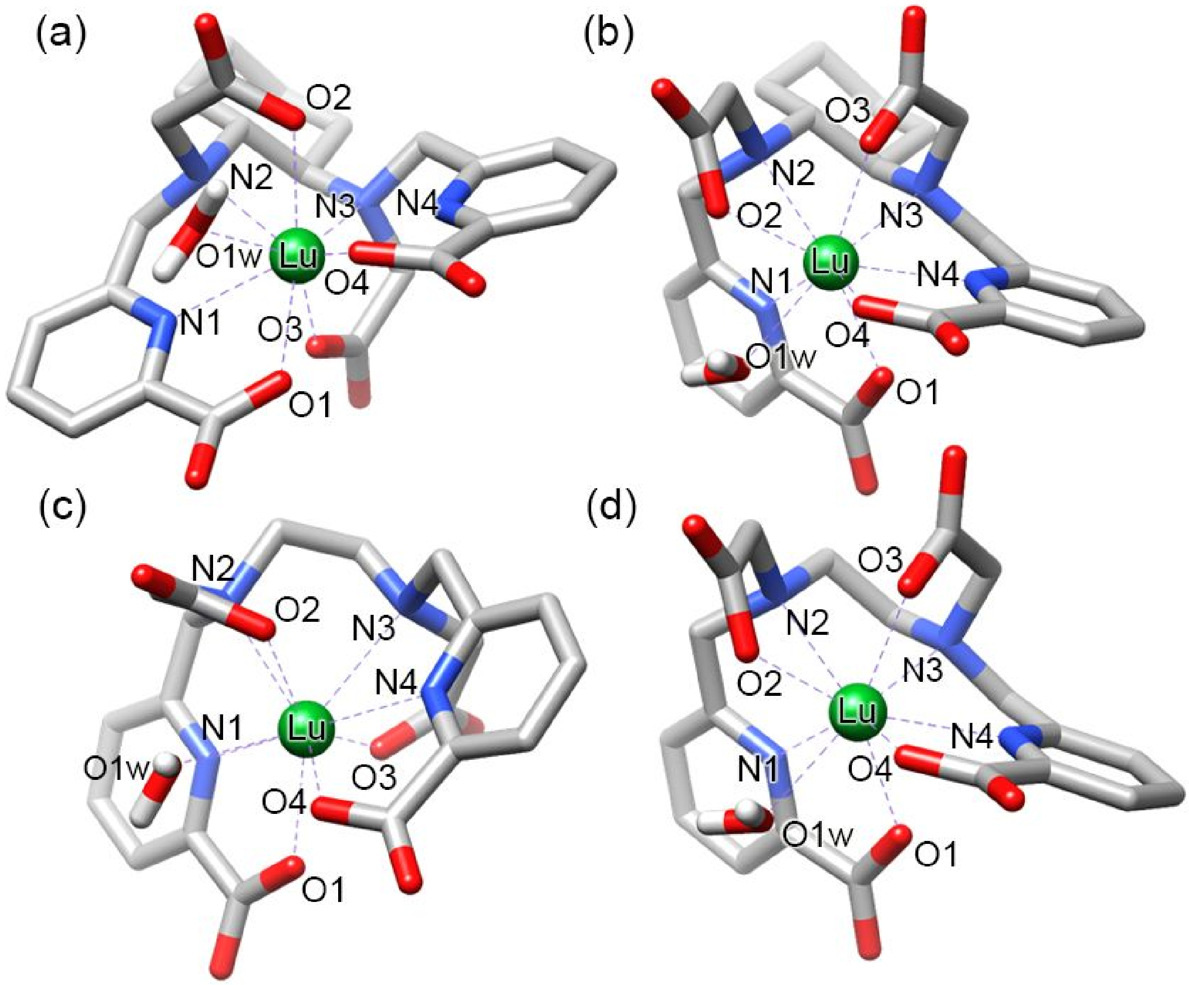

Figure S27: Optimized structures for the (a) $[Lu(\textit{CHX}OCTAPA)_{S,S}(H_2O)]^{-}\cdot 2H_2O$, (b) $[Lu(\textit{CHX}OCTAPA)_{S,R}(H_2O)]^{-}\cdot 2H_2O$, (c) $[Lu(OCTAPA)_{S,S}(H_2O)]^{-}\cdot 2H_2O$, and (d) $[Lu(OCTAPA)_{S,R}(H_2O)]^{-}\cdot 2H_2O$ systems obtained with DFT calculations (0 Imaginary Frequencies).

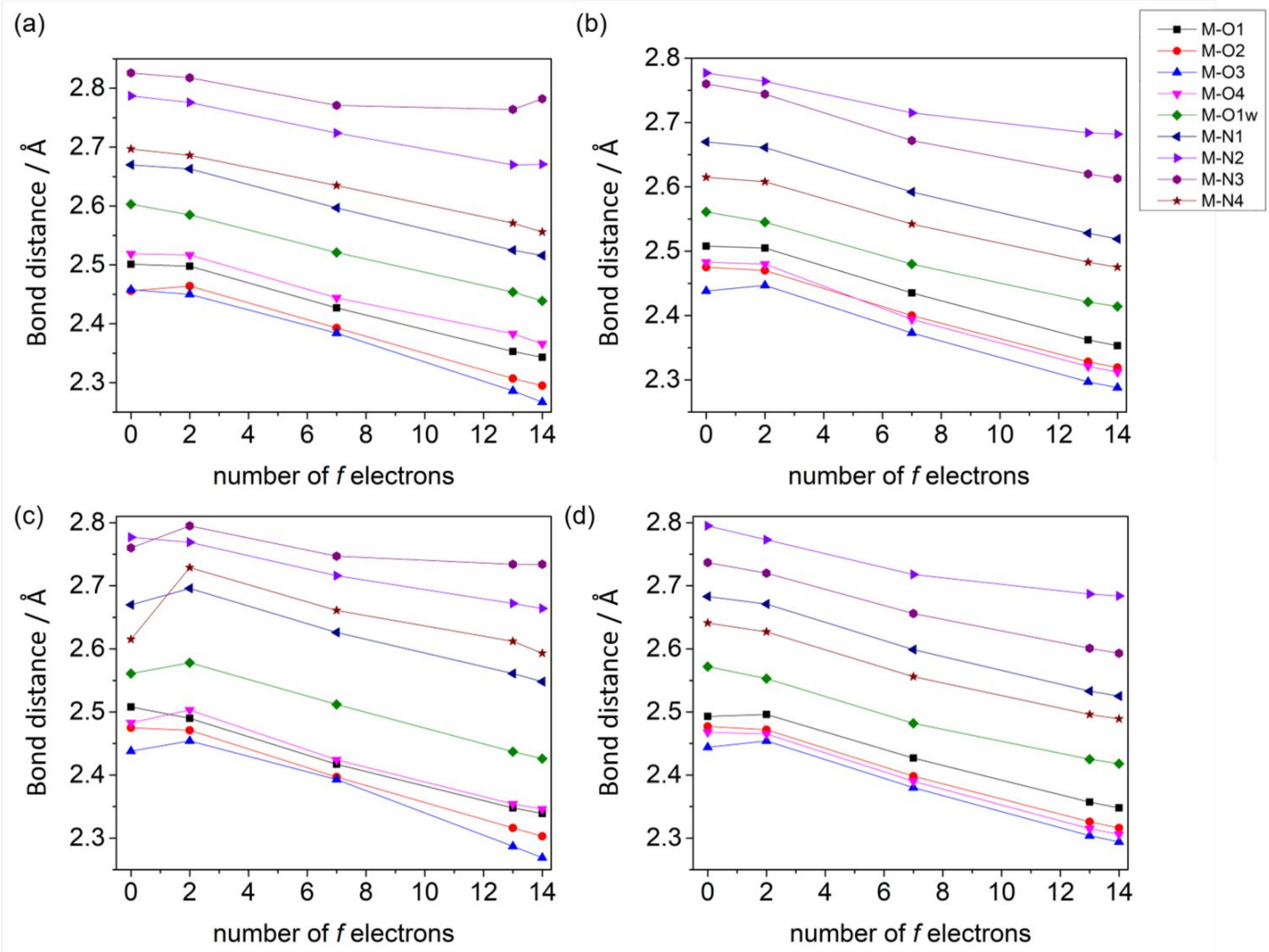

Figure S28: Evolution of the calculated distances between the metal and the donor atoms of the ligand. (a) $[Ln(\mathit{CHX}OCTAPA_{S,S})]^-$, (b) $[Ln(\mathit{CHX}OCTAPA_{S,R})]^-$, (c) $[Ln(OCTAPA_{S,S})]^-$, (d) $[Ln(OCTAPA_{S,R})]^-$

Table S3: Distances / Å between the metal centre and the different donor atoms in the calculated structures.

Compound	Distances / Å								
	M-O1	M-O2	M-O3	M-O4	M-O1$_w$	M-N1	M-N2	M-N3	M-N4
[La(*CHX*OCTAPA)$_{S,S}$(H$_2$O)]$^-$	2.501	2.456	2.458	2.519	2.603	2.670	2.787	2.826	2.697
[La(*CHX*OCTAPA)$_{S,R}$(H$_2$O)]$^-$	2.508	2.475	2.438	2.483	2.561	2.670	2.777	2.760	2.615
[La(OCTAPA)$_{S,S}$(H$_2$O)]$^-$	2.493	2.466	2.453	2.513	2.595	2.702	2.774	2.805	2.742
[La(OCTAPA)$_{cis}$(H$_2$O)]$^-$	2.493	2.477	2.444	2.468	2.572	2.683	2.795	2.737	2.641
[Pr(*CHX*OCTAPA)$_{S,S}$(H$_2$O)]$^-$	2.498	2.464	2450	2.517	2.585	2.663	2.776	2.818	2.686
[Pr(*CHX*OCTAPA)$_{S,R}$(H$_2$O)]$^-$	2.505	2.470	2.447	2.480	2.545	2.661	2.764	2.744	2.608
[Pr(OCTAPA)$_{S,S}$(H$_2$O)]$^-$	2.490	2.471	2.454	2.503	2.578	2.696	2.769	2.795	2.729
[Pr(OCTAPA)$_{S,R}$(H$_2$O)]$^-$	2.496	2.472	2.454	2.465	2.553	2.671	2.773	2.720	2.627
[Gd(*CHX*OCTAPA)$_{S,S}$(H$_2$O)]$^-$	2.427	2.393	2.384	2.444	2.521	2.597	2.724	2.771	2.635
[Gd(*CHX*OCTAPA)$_{S,R}$(H$_2$O)]$^-$	2.435	2.400	2.373	2.394	2.480	2.592	2.715	2.672	2.542
[Gd(OCTAPA)$_{S,S}$(H$_2$O)]$^-$	2.417	2.397	2.393	2.424	2.512	2.626	2.716	2.747	2.661
[Gd(OCTAPA)$_{S,R}$(H$_2$O)]$^-$	2.427	2.398	2.380	2.390	2.482	2.599	2.718	2.656	2.556
[Yb(*CHX*OCTAPA)$_{S,S}$(H$_2$O)]$^-$	2.353	2.307	2.286	2.383	2.454	2.525	2.670	2.764	2.571
[Yb(*CHX*OCTAPA)$_{S,R}$(H$_2$O)]$^-$	2.362	2.328	2.297	2.321	2.421	2.528	2.684	2.620	2.483
[Yb(OCTAPA)$_{S,S}$(H$_2$O)]$^-$	2.348	2.316	2.287	2.354	2.437	2.561	2.672	2.734	2.612
[Yb(OCTAPA)$_{S,R}$(H$_2$O)]$^-$	2.357	2.326	2.304	2.315	2.425	2.533	2.687	2.601	2.496
[Lu(*CHX*OCTAPA)$_{S,S}$(H$_2$O)]$^-$	2.343	2.295	2.267	2.366	2.439	2.516	2.671	2.782	2.556
[Lu(*CHX*OCTAPA)$_{S,R}$(H$_2$O)]$^-$	2.353	2.319	2.288	2.312	2.414	2.519	2.682	2.613	2.475
[Lu(OCTAPA)$_{S,S}$(H$_2$O)]$^-$	2.339	2.303	2.269	2.346	2.426	2.548	2.664	2.734	2.593
[Lu(OCTAPA)$_{S,R}$(H$_2$O)]$^-$	2.348	2.316	2.294	2.306	2.418	2.525	2.684	2.593	2.489

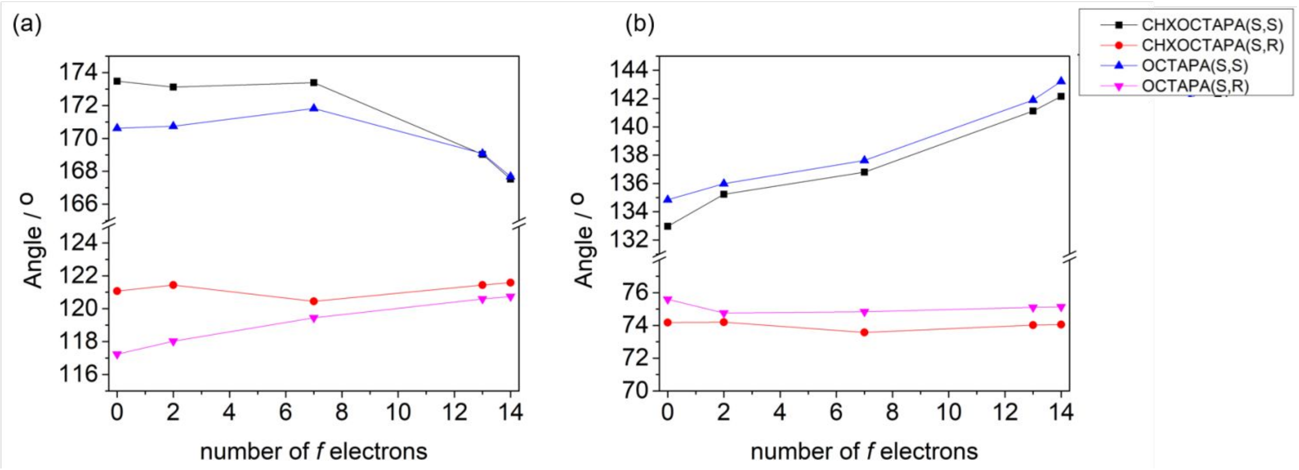

Figure S29: Evolution of the calculated angles / ^{0} (a) N1-M-N4 and (b) O2-M-O3 for the different calculated structures.

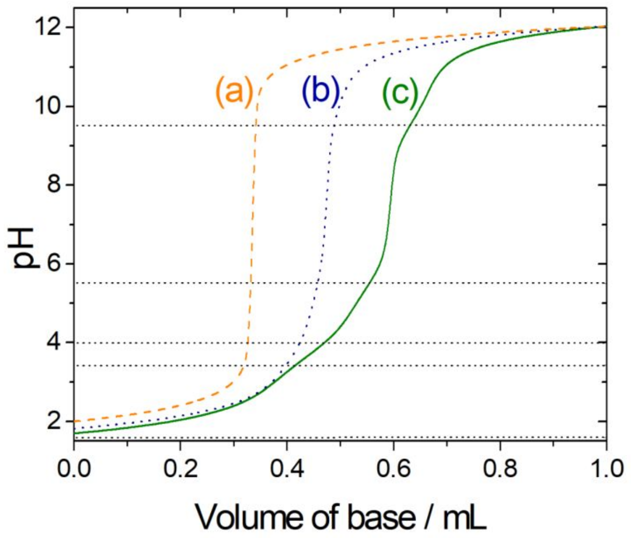

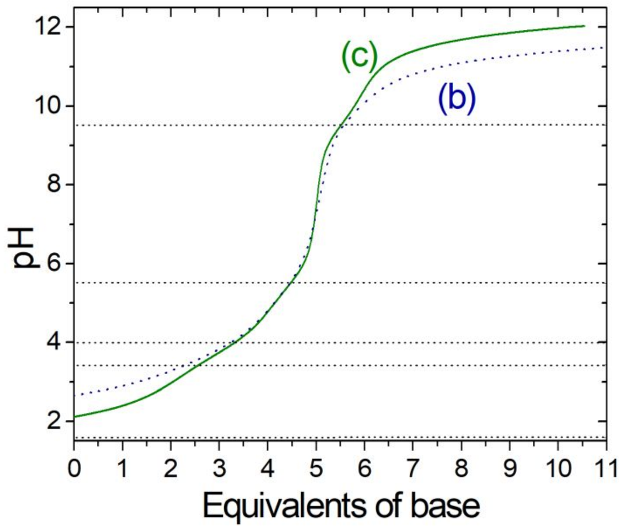

Figure S30: Top: Potentiometric titration curves simulated for three different conditions: (a) Acid concentration in the cell (10 mL) 0.1 M, base concentration in the burette 0.3 M, ligand concentration 0 M. (b) Acid concentration in the cell (10 mL) 0.2 M, base concentration in the burette 0.4 M, ligand concentration 1.1×10^{-3} M. (c) Acid concentration in the cell (10 mL) 0.4 M, base concentration in the burette 0.6 M, ligand concentration 4.4×10^{-3} M. The dashed horizontal lines correspond to the pK_a values of CHXOCTAPA determined in this work. Increasing ligand concentration causes more drastic changes in the shape of the potentiometric curve, allowing for a more accurate determination of protonation constants. Bottom: Titration curves (b) and (c) represented vs equivalents of added base.

Table S4: Selected angles / ^{0} in the calculated structures.

Compound	Angles / ^{0}	
	N1-M-N1	O2-M-O3
[La(*CHX*OCTAPA)$_{S,S}$(H$_2$O)]$^-$	173.477	132.964
[La(*CHX*OCTAPA)$_{S,R}$(H$_2$O)]$^-$	121.076	74.175
[La(OCTAPA)$_{S,S}$(H$_2$O)]$^-$	170.614	134.846
[La(OCTAPA)$_{S,R}$(H$_2$O)]$^-$	117.231	75.601
[Pr(*CHX*OCTAPA)$_{S,S}$(H$_2$O)]$^-$	173.123	135.232
[Pr(*CHX*OCTAPA)$_{S,R}$(H$_2$O)]$^-$	121.443	74.194
[Pr(OCTAPA)$_{S,S}$(H$_2$O)]$^-$	170.744	135.983
[Pr(OCTAPA)$_{S,R}$(H$_2$O)]$^-$	118.024	74.759
[Gd(*CHX*OCTAPA)$_{S,S}$(H$_2$O)]$^-$	173.393	136.801
[Gd(*CHX*OCTAPA)$_{S,R}$(H$_2$O)]$^-$	120.453	73.570
[Gd(OCTAPA)$_{S,S}$(H$_2$O)]$^-$	171.825	137.623
[Gd(OCTAPA)$_{S,R}$(H$_2$O)]$^-$	119.452	74.832
[Yb(*CHX*OCTAPA)$_{S,S}$(H$_2$O)]$^-$	169.029	141.121
[Yb(*CHX*OCTAPA)$_{S,R}$(H$_2$O)]$^-$	121.441	74.020
[Yb(OCTAPA)$_{S,S}$(H$_2$O)]$^-$	169.086	141.894
[Yb(OCTAPA)$_{S,R}$(H$_2$O)]$^-$	120.588	75.108
[Lu(*CHX*OCTAPA)$_{S,S}$(H$_2$O)]$^-$	167.532	142.151
[Lu(*CHX*OCTAPA)$_{S,R}$(H$_2$O)]$^-$	121.578	74.057
[Lu(OCTAPA)$_{S,S}$(H$_2$O)]$^-$	167.682	143.211
[Lu(OCTAPA)$_{S,R}$(H$_2$O)]$^-$	120.737	75.141

Table S5: Optimized Cartesian coordinates (Å) of the [La(*CHX*OCTAPA)$_{S,S}$(H$_2$O)]$^{-}$·2H$_2$O system obtained with DFT calculations (0 Imaginary Frequencies).

Center Number	Atomic Number	Coordinates (Angstroms) X	Y	Z
1	7	-2.174504	1.855909	-0.154235
2	7	2.436380	-0.838842	-0.577532
3	7	0.254141	-1.886761	0.885218
4	7	-2.114285	-0.919121	-0.714067
5	8	-0.648292	0.404040	1.966706
6	8	0.066367	-0.802269	-2.315946
7	6	-4.558772	3.239142	-0.349949
8	6	3.719255	-2.796061	-0.158788
9	6	2.509962	-2.101775	-0.165108
10	6	1.219343	-2.781257	0.221511
11	6	-1.108865	-2.508170	0.977925
12	6	0.725042	-1.535782	2.237026
13	6	4.774775	-0.837266	-1.047622
14	6	3.533380	-0.217974	-1.015202
15	6	3.339436	1.228959	-1.469890
16	6	4.864312	-2.153457	-0.609664
17	6	-3.354977	3.927801	-0.263735
18	6	-2.183976	3.191850	-0.155941
19	6	-0.827142	3.863465	0.046663
20	6	-4.549269	1.851086	-0.302581
21	6	-3.327524	1.189182	-0.194892
22	6	-3.259297	-0.307333	-0.021101
23	6	-1.918694	-2.353035	-0.321246
24	6	-2.306922	-0.839528	-2.172253
25	1	-5.495461	3.775690	-0.440589
26	1	3.754587	-3.819379	0.193078
27	1	0.763714	-3.146200	-0.703888
28	1	1.463098	-3.651260	0.840646
29	1	-1.631832	-1.949787	1.760878
30	1	1.782973	-1.267404	2.183409
31	1	0.627403	-2.362216	2.949286
32	1	5.632714	-0.286622	-1.409415
33	1	5.815204	-2.672220	-0.615625
34	1	-3.300800	5.007738	-0.268918
35	1	-5.470278	1.283596	-0.345547
36	1	-3.150252	-0.497225	1.051467
37	1	-4.213720	-0.734677	-0.346887
38	1	-1.333920	-2.803497	-1.131011
39	1	-2.607962	0.181512	-2.429840
40	1	-3.094112	-1.510392	-2.531914
41	8	2.141449	1.599461	-1.590780
42	8	4.359536	1.908425	-1.652476
43	8	0.115238	3.053624	0.342207
44	8	-0.761110	5.082811	-0.048087
45	8	2.074919	1.140376	1.430648
46	6	-3.257697	-3.113488	-0.229180
47	1	-3.884598	-2.649703	0.537669
48	1	-3.792309	-3.022977	-1.177691

49	6	-1.036607	-3.987881	1.407607
50	1	-0.467617	-4.553373	0.664316
51	1	-0.494486	-4.066750	2.352820
52	6	-2.404359	-4.654052	1.516084
53	6	-3.096590	-4.579189	0.159951
54	1	-2.280008	-5.690129	1.838896
55	1	-3.018047	-4.149437	2.270710
56	1	-2.498988	-5.112138	-0.588129
57	1	-4.078167	-5.057370	0.191495
58	1	1.767347	1.768473	2.112604
59	1	3.034108	1.285342	1.322444
60	6	-1.002789	-1.127665	-2.933555
61	6	0.019156	-0.293949	2.796144
62	8	-1.081265	-1.608052	-4.059978
63	8	0.194208	-0.014517	3.985294
64	8	0.629743	2.719671	3.177723
65	1	0.151638	2.819491	2.339915
66	1	0.259223	1.913829	3.575523
67	8	4.796690	1.672219	1.118379
68	1	4.914555	1.858607	0.171276
69	1	5.365839	0.918321	1.298682
70	57	0.219317	0.625330	-0.320774

```
---------------------------------------------------------------------
E(RM062X) = -2414.907665 (Hartree)
Zero-point correction=                           0.552433 (Hartree/Particle)
Thermal correction to Energy=                    0.591358
Thermal correction to Enthalpy=                  0.592302
Thermal correction to Gibbs Free Energy=         0.483460
Sum of electronic and zero-point Energies=          -2414.355233
Sum of electronic and thermal Energies=             -2414.316308
Sum of electronic and thermal Enthalpies=           -2414.315363
Sum of electronic and thermal Free Energies=        -2414.424205
```

Table S6: Optimized Cartesian coordinates (Å) of the [La(*CHX*OCTAPA)$_{S,R}$(H_2O)]$^-$·2H_2O system obtained with DFT calculations (0 Imaginary Frequencies).

Center Number	Atomic Number	Coordinates (Angstroms) X	Y	Z
1	6	-0.589786	-1.259568	-2.994635
2	6	-1.937729	-1.205468	-2.270968
3	6	-3.143859	-1.462375	-2.909514
4	1	-3.141902	-1.720834	-3.959557
5	6	-4.316584	-1.365253	-2.165901
6	1	-5.277544	-1.547958	-2.631217
7	6	-4.246761	-1.026668	-0.818455
8	1	-5.141684	-0.937636	-0.215238
9	6	-2.994377	-0.801212	-0.252540
10	6	-2.786086	-0.520932	1.213149
11	1	-3.726787	-0.225885	1.687576
12	1	-2.478442	-1.467137	1.669672
13	6	-1.985549	1.853025	1.034653
14	1	-1.564419	2.488586	1.816983

15	6	-3.484600	2.189563	1.000183
16	1	-3.992021	1.586014	0.240219
17	1	-3.925292	1.937466	1.968410
18	6	-3.728507	3.662766	0.681162
19	1	-4.802433	3.861537	0.672075
20	1	-3.289785	4.289974	1.465535
21	6	-3.099970	4.012218	-0.662847
22	1	-3.265366	5.063425	-0.908926
23	1	-3.576276	3.417994	-1.451350
24	6	-1.600913	3.720879	-0.636475
25	1	-1.125804	4.377550	0.101328
26	1	-1.165090	3.962498	-1.607608
27	6	-1.288221	2.254734	-0.289192
28	1	-1.681807	1.623377	-1.093352
29	6	0.821499	2.178534	-1.554748
30	1	0.799671	3.227977	-1.866917
31	1	0.280188	1.581420	-2.294546
32	6	2.262382	1.713770	-1.522712
33	6	3.305406	2.428267	-2.104638
34	1	3.109796	3.369573	-2.602510
35	6	4.595505	1.914290	-2.020621
36	1	5.424145	2.453952	-2.462739
37	6	4.814230	0.713958	-1.355166
38	1	5.798722	0.278769	-1.251600
39	6	3.720182	0.063605	-0.800056
40	6	3.858909	-1.256221	-0.036994
41	6	-1.370353	0.390930	2.905938
42	1	-2.260092	0.536978	3.531540
43	1	-0.659925	1.187555	3.129414
44	6	-0.666207	-0.902973	3.355679
45	6	0.904408	2.782245	0.776333
46	1	0.202398	3.248731	1.469134
47	1	1.462484	3.598863	0.309870
48	6	1.864245	1.961097	1.656655
49	7	-1.879224	-0.888698	-0.976464
50	7	-1.692873	0.442026	1.466888
51	7	0.179265	1.981468	-0.236386
52	7	2.487998	0.560362	-0.893880
53	8	-0.162345	-1.632306	2.427053
54	8	-0.608906	-1.124895	4.557434
55	8	2.752171	-1.757422	0.366292
56	8	4.979539	-1.718685	0.122771
57	8	0.399537	-0.913159	-2.275697
58	8	-0.584573	-1.611471	-4.169441
59	8	1.502250	0.758081	1.882785
60	8	2.852096	2.528969	2.111970
61	8	0.038225	-3.214732	-0.131309
62	1	0.484706	-3.699941	0.588472
63	1	-0.874164	-3.546839	-0.214222
64	8	-2.589875	-3.990373	-0.514155
65	8	1.630177	-3.701343	2.041130
66	1	1.053293	-3.053751	2.485072
67	1	2.289280	-3.151268	1.583779
68	1	-3.247554	-3.903047	0.182167

69	1	-3.007115	-3.622900	-1.300921
70	57	0.500399	-0.721012	0.223122

```
E(RM062X) = -2414.900587 (Hartree)
Zero-point correction=                          0.551305 (Hartree/Particle)
Thermal correction to Energy=                   0.590749
Thermal correction to Enthalpy=                 0.591693
Thermal correction to Gibbs Free Energy=        0.480989
Sum of electronic and zero-point Energies=         -2414.349282
Sum of electronic and thermal Energies=            -2414.309839
Sum of electronic and thermal Enthalpies=          -2414.308894
Sum of electronic and thermal Free Energies=       -2414.419598
```

Table S7: Optimized Cartesian coordinates (Å) of the $[La(OCTAPA)_{S,S}(H_2O)]^{-}\cdot 2H_2O$ system obtained with DFT calculations (0 Imaginary Frequencies).

Center Number	Atomic Number	Coordinates (Angstroms) X	Y	Z
1	7	2.823288	-0.314911	-0.198662
2	7	-2.540817	0.489616	-0.336428
3	7	-0.898415	1.578224	1.622553
4	7	1.643098	2.182064	0.064305
5	8	0.821607	-0.423468	2.001765
6	8	-0.257316	1.814726	-1.799360
7	6	5.567696	-0.562432	-0.408671
8	6	-4.463545	1.555818	0.578196
9	6	-3.083407	1.396748	0.476121
10	6	-2.122274	2.286428	1.225567
11	6	0.142043	2.548216	2.024565
12	6	-1.171218	0.667176	2.744815
13	6	-4.716245	-0.183665	-1.049499
14	6	-3.331665	-0.280656	-1.085738
15	6	-2.622279	-1.301621	-1.977247
16	6	-5.289207	0.754981	-0.200161
17	6	4.741183	-1.631988	-0.729904
18	6	3.369271	-1.465603	-0.598098
19	6	2.395217	-2.619315	-0.835304
20	6	4.999244	0.619825	0.049656
21	6	3.612819	0.702720	0.148418
22	6	2.919095	1.907565	0.733665
23	6	0.857819	3.165983	0.835442
24	6	1.883789	2.714244	-1.284398
25	1	6.643323	-0.649951	-0.502477
26	1	-4.875361	2.292815	1.256069
27	1	-1.845466	3.100402	0.550452
28	1	-2.620705	2.735348	2.095161
29	1	0.858836	2.022624	2.655184
30	1	-2.071406	0.088530	2.524459
31	1	-1.336243	1.214047	3.682238
32	1	-5.308731	-0.835887	-1.676743
33	1	-6.365698	0.858950	-0.139941
34	1	5.127104	-2.583613	-1.068535

35	1	5.613741	1.466617	0.328283
36	1	2.715822	1.676332	1.782768
37	1	3.582108	2.782951	0.703264
38	1	0.130705	3.614504	0.156372
39	1	2.619988	2.080985	-1.789763
40	1	2.289600	3.733264	-1.251415
41	8	-1.370591	-1.176961	-2.054175
42	8	-3.324669	-2.168611	-2.515448
43	8	1.192763	-2.375565	-0.475093
44	8	2.834110	-3.663969	-1.299383
45	8	-1.410121	-1.835320	0.905832
46	1	-0.886696	-2.506761	1.385973
47	1	-2.245242	-2.262668	0.635880
48	6	0.605221	2.693588	-2.138318
49	6	-0.047711	-0.361497	2.929179
50	8	0.521444	3.480445	-3.075357
51	8	-0.090215	-1.095699	3.920073
52	8	0.530255	-3.309951	2.195983
53	1	1.013506	-2.922502	1.449610
54	1	0.569367	-2.631023	2.890939
55	8	-3.740789	-3.140314	0.099444
56	1	-3.807941	-3.003615	-0.860600
57	1	-4.545923	-2.760801	0.463682
58	57	0.091627	-0.120396	-0.333685
59	1	-0.301791	3.345467	2.636392
60	1	1.507394	3.979994	1.186435

```
E(RM062X) = -2258.891168 (Hartree)
Zero-point correction=                      0.457909 (Hartree/Particle)
Thermal correction to Energy=               0.493374
Thermal correction to Enthalpy=             0.494319
Thermal correction to Gibbs Free Energy=    0.392775
Sum of electronic and zero-point Energies=     -2258.433259
Sum of electronic and thermal Energies=        -2258.397794
Sum of electronic and thermal Enthalpies=      -2258.396849
Sum of electronic and thermal Free Energies=   -2258.498393
```

Table S8: Optimized Cartesian coordinates (Å) of the $[La(OCTAPA)_{S,R}(H_2O)]^{-}\cdot 2H_2O$ system obtained with DFT calculations (0 Imaginary Frequencies).

Center Number	Atomic Number	Coordinates (Angstroms) X	Y	Z
1	6	-0.712829	-3.088933	0.295012
2	6	-2.029771	-2.467883	-0.176712
3	6	-3.155725	-3.236195	-0.441580
4	1	-3.110638	-4.305964	-0.290669
5	6	-4.297710	-2.593818	-0.909159
6	1	-5.190697	-3.160892	-1.142234
7	6	-4.280891	-1.214007	-1.080709
8	1	-5.150916	-0.683892	-1.446605
9	6	-3.119622	-0.514969	-0.761218
10	6	-3.052554	0.989192	-0.794481

11	1	-3.820413	1.389637	-1.469149
12	1	-3.289886	1.332520	0.217915
13	6	-1.402461	1.343701	-2.572044
14	1	-0.855564	2.227005	-2.907748
15	6	-0.567186	0.099962	-2.889008
16	1	-1.106611	-0.810622	-2.626683
17	6	1.452582	-1.164294	-2.386312
18	1	1.775955	-1.269237	-3.430284
19	1	0.781526	-1.995132	-2.149823
20	6	2.648749	-1.244337	-1.459373
21	6	3.838453	-1.873442	-1.813238
22	1	3.949585	-2.315348	-2.795465
23	6	4.874355	-1.913612	-0.885833
24	1	5.811117	-2.395013	-1.139076
25	6	4.699973	-1.327389	0.362039
26	1	5.474058	-1.328859	1.117097
27	6	3.480943	-0.721745	0.636642
28	6	3.184193	-0.081128	1.995036
29	6	-1.697556	2.940462	-0.731494
30	1	-2.615741	3.461166	-1.031545
31	1	-0.858531	3.443539	-1.213177
32	6	-1.491859	3.144513	0.779339
33	6	1.550331	1.248108	-2.576319
34	1	1.357920	1.522768	-3.618696
35	1	2.604901	0.972346	-2.501480
36	6	1.370876	2.474843	-1.669796
37	7	-2.024672	-1.140634	-0.327394
38	7	-1.722320	1.521263	-1.133563
39	7	0.720962	0.095208	-2.168668
40	7	2.496560	-0.680733	-0.259902
41	8	-1.157030	2.106439	1.457614
42	8	-1.636967	4.276652	1.221141
43	8	1.974820	0.315835	2.143262
44	8	4.088183	-0.008858	2.814204
45	8	0.252744	-2.266571	0.367737
46	8	-0.701644	-4.290548	0.540155
47	8	1.188582	2.215270	-0.435758
48	8	1.426882	3.592797	-2.179675
49	8	-1.037215	-0.624163	2.630443
50	1	-0.833680	0.030874	3.324062
51	1	-2.003970	-0.741541	2.596271
52	8	-3.756182	-1.087223	2.347266
53	8	0.099203	1.544372	3.864576
54	1	-0.339277	2.041861	3.150767
55	1	0.948300	1.264169	3.483589
56	1	-4.401494	-0.385864	2.219088
57	1	-4.006763	-1.779199	1.725723
58	57	0.133697	0.220185	0.501867
59	1	-0.396757	0.081402	-3.975398
60	1	-2.321591	1.301772	-3.168700

E(RM062X) = -2258.882402 (Hartree)
Zero-point correction= 0.457216 (Hartree/Particle)
Thermal correction to Energy= 0.493321

```
Thermal correction to Enthalpy=                  0.494266
Thermal correction to Gibbs Free Energy=         0.390253
Sum of electronic and Zero-point Energies=          -2258.425186
Sum of electronic and Thermal Energies=             -2258.389080
Sum of electronic and Thermal Enthalpies=           -2258.388136
Sum of electronic and Thermal Free Energies=        -2258.492149
```

Table S9: Optimized Cartesian coordinates (Å) of the [Pr(*CHX*OCTAPA)$_{S,S}$(H_2O)]$^-$·2H_2O system obtained with DFT calculations (0 Imaginary Frequencies).

Center Number	Atomic Number	Coordinates (Angstroms) X	Y	Z
1	7	-2.152400	1.861841	-0.161557
2	7	2.425121	-0.856630	-0.575156
3	7	0.242511	-1.898839	0.890494
4	7	-2.109202	-0.907624	-0.727263
5	8	-0.669265	0.384919	1.995630
6	8	0.060395	-0.689288	-2.358206
7	6	-4.526496	3.262364	-0.348815
8	6	3.707596	-2.815135	-0.163352
9	6	2.499727	-2.118820	-0.161486
10	6	1.209727	-2.796093	0.232243
11	6	-1.125022	-2.513859	0.963531
12	6	0.701601	-1.561139	2.250926
13	6	4.758286	-0.859382	-1.065481
14	6	3.518814	-0.236756	-1.022280
15	6	3.327794	1.213525	-1.471272
16	6	4.849412	-2.174905	-0.625783
17	6	-3.317600	3.942138	-0.262654
18	6	-2.151738	3.197835	-0.157987
19	6	-0.791144	3.859346	0.054622
20	6	-4.527223	1.874239	-0.303336
21	6	-3.310176	1.203458	-0.200066
22	6	-3.251281	-0.293873	-0.030200
23	6	-1.921124	-2.345544	-0.343268
24	6	-2.306696	-0.817356	-2.185391
25	1	-5.459354	3.805926	-0.436961
26	1	3.744126	-3.837835	0.190163
27	1	0.754318	-3.170432	-0.689518
28	1	1.453155	-3.661707	0.857477
29	1	-1.653417	-1.958121	1.744510
30	1	1.761194	-1.297494	2.208738
31	1	0.593772	-2.394770	2.952912
32	1	5.613930	-0.310669	-1.435290
33	1	5.799461	-2.695057	-0.639040
34	1	-3.255602	5.021642	-0.264432
35	1	-5.452332	1.313354	-0.344435
36	1	-3.143072	-0.488183	1.041596
37	1	-4.207556	-0.714607	-0.358339
38	1	-1.331691	-2.791164	-1.152935
39	1	-2.628318	0.200001	-2.430945
40	1	-3.081292	-1.500402	-2.547915

41	8	2.133144	1.600911	-1.559824
42	8	4.351970	1.880217	-1.678247
43	8	0.143092	3.043636	0.358280
44	8	-0.716828	5.078390	-0.037389
45	8	2.065002	1.099304	1.457109
46	6	-3.264003	-3.101253	-0.266635
47	1	-3.893923	-2.639067	0.498695
48	1	-3.791664	-3.002813	-1.217976
49	6	-1.063358	-3.996152	1.385692
50	1	-0.489042	-4.560706	0.645884
51	1	-0.531735	-4.081816	2.336176
52	6	-2.434880	-4.656751	1.477167
53	6	-3.113447	-4.570134	0.114940
54	1	-2.318027	-5.695303	1.794671
55	1	-3.053374	-4.154051	2.229107
56	1	-2.511388	-5.101481	-0.630657
57	1	-4.097731	-5.043315	0.134026
58	1	1.763387	1.726799	2.142939
59	1	3.019530	1.257145	1.326109
60	6	-1.001220	-1.068916	-2.956029
61	6	-0.004646	-0.322073	2.819003
62	8	-1.072554	-1.571268	-4.073471
63	8	0.169226	-0.054990	4.011114
64	8	0.634455	2.684713	3.199597
65	1	0.159230	2.800448	2.362271
66	1	0.245925	1.885690	3.593434
67	8	4.774793	1.659630	1.103362
68	1	4.899406	1.848692	0.157932
69	1	5.350216	0.911353	1.287377
70	59	0.222624	0.613198	-0.289651

```
E(RM062X) = -2012.275649 (Hartree)
Zero-point correction=                           0.552745 (Hartree/Particle)
Thermal correction to Energy=                    0.591528
Thermal correction to Enthalpy=                  0.592472
Thermal correction to Gibbs Free Energy=         0.484276
Sum of electronic and Zero-point Energies=          -2011.722905
Sum of electronic and Thermal Energies=             -2011.684121
Sum of electronic and Thermal Enthalpies=           -2011.683177
Sum of electronic and Thermal Free Energies=        -2011.791374
```

Table S10: Optimized Cartesian coordinates (Å) of the [Pr(*CHX*OCTAPA)$_{S,R}$(H_2O)]$^{-}$·2H_2O system obtained with DFT calculations (0 Imaginary Frequencies).

Center Number	Atomic Number	Coordinates (Angstroms) X	Y	Z
1	6	-0.580210	-1.264380	-2.989639
2	6	-1.929810	-1.215456	-2.267061
3	6	-3.133547	-1.479737	-2.907195
4	1	-3.128068	-1.741978	-3.956260
5	6	-4.308492	-1.384477	-2.166822
6	1	-5.267449	-1.573089	-2.633899

7	6	-4.243849	-1.040068	-0.820566
8	1	-5.140630	-0.952458	-0.219940
9	6	-2.993895	-0.807416	-0.252611
10	6	-2.788626	-0.520438	1.212151
11	1	-3.729494	-0.222473	1.683851
12	1	-2.482400	-1.464658	1.673742
13	6	-1.989448	1.853735	1.030711
14	1	-1.566729	2.489875	1.811638
15	6	-3.488909	2.188244	0.999310
16	1	-3.997412	1.582397	0.241868
17	1	-3.926753	1.937751	1.969219
18	6	-3.735655	3.660358	0.677541
19	1	-4.809882	3.857511	0.671523
20	1	-3.295255	4.290074	1.458934
21	6	-3.112151	4.007143	-0.669455
22	1	-3.280331	5.057283	-0.918059
23	1	-3.589778	3.409691	-1.454695
24	6	-1.612417	3.718988	-0.646866
25	1	-1.136364	4.379405	0.086830
26	1	-1.180181	3.957661	-1.620352
27	6	-1.296190	2.254637	-0.296106
28	1	-1.690073	1.620670	-1.098094
29	6	0.815486	2.183943	-1.563470
30	1	0.793879	3.234099	-1.872603
31	1	0.274826	1.588320	-2.304938
32	6	2.256388	1.718064	-1.529777
33	6	3.301711	2.426492	-2.114669
34	1	3.109660	3.365942	-2.617420
35	6	4.590045	1.908211	-2.027110
36	1	5.420633	2.443065	-2.471411
37	6	4.805136	0.710152	-1.356173
38	1	5.788434	0.272791	-1.250699
39	6	3.708785	0.065679	-0.798403
40	6	3.841440	-1.252757	-0.030494
41	6	-1.367331	0.389745	2.901394
42	1	-2.255463	0.532277	3.529700
43	1	-0.657579	1.187142	3.123976
44	6	-0.660006	-0.904988	3.346579
45	6	0.895813	2.789107	0.767183
46	1	0.191627	3.261863	1.453388
47	1	1.455748	3.601959	0.296813
48	6	1.853869	1.978914	1.661190
49	7	-1.876935	-0.893584	-0.973640
50	7	-1.694698	0.442710	1.463219
51	7	0.172447	1.984165	-0.245091
52	7	2.479624	0.567530	-0.895707
53	8	-0.150849	-1.629483	2.417163
54	8	-0.607542	-1.132545	4.547453
55	8	2.734005	-1.752751	0.372389
56	8	4.961005	-1.716799	0.132144
57	8	0.407368	-0.907153	-2.274565
58	8	-0.574077	-1.621659	-4.162934
59	8	1.505691	0.773827	1.889865
60	8	2.827765	2.564131	2.125891

61	8	0.046657	-3.182645	-0.126293
62	1	0.495933	-3.672792	0.588668
63	1	-0.863801	-3.519501	-0.211866
64	8	-2.570351	-3.992305	-0.504000
65	8	1.633997	-3.702708	2.042187
66	1	1.054241	-3.055306	2.483141
67	1	2.288656	-3.148848	1.582299
68	1	-3.226802	-3.920555	0.195229
69	1	-2.998950	-3.633622	-1.288701
70	59	0.493365	-0.701215	0.220244

```
E(RM062X) = -2012.270500 (Hartree)
Zero-point correction=                          0.551693 (Hartree/Particle)
Thermal correction to Energy=                   0.590994
Thermal correction to Enthalpy=                 0.591938
Thermal correction to Gibbs Free Energy=        0.481456
Sum of electronic and Zero-point Energies=         -2011.718807
Sum of electronic and Thermal Energies=            -2011.679506
Sum of electronic and Thermal Enthalpies=          -2011.678561
Sum of electronic and Thermal Free Energies=       -2011.789043
```

Table S11: Optimized Cartesian coordinates (Å) of the $[Pr(OCTAPA)_{S,S}(H_2O)]^{-}\cdot 2H_2O$ system obtained with DFT calculations (0 Imaginary Frequencies).

Center Number	Atomic Number	Coordinates (Angstroms) X	Y	Z
1	7	-2.810368	0.300747	-0.217127
2	7	2.539833	-0.477265	-0.329194
3	7	0.904491	-1.583084	1.627356
4	7	-1.621028	-2.187982	0.042651
5	8	-0.842017	0.400855	2.020892
6	8	0.256866	-1.765559	-1.844860
7	6	-5.556966	0.552309	-0.389963
8	6	4.471604	-1.538970	0.570560
9	6	3.090419	-1.382748	0.479421
10	6	2.138506	-2.277109	1.235514
11	6	-0.130471	-2.567560	2.008866
12	6	1.158461	-0.681264	2.762182
13	6	4.707359	0.201474	-1.058761
14	6	3.322353	0.296396	-1.083748
15	6	2.605841	1.321493	-1.966284
16	6	5.289041	-0.736732	-0.214842
17	6	-4.733375	1.621724	-0.719281
18	6	-3.360347	1.453247	-0.606700
19	6	-2.388997	2.607536	-0.851265
20	6	-4.983909	-0.631764	0.057156
21	6	-3.596405	-0.716759	0.137721
22	6	-2.898819	-1.924209	0.713038
23	6	-0.831506	-3.174759	0.805650
24	6	-1.859781	-2.712728	-1.309926
25	1	-6.633634	0.641698	-0.468612
26	1	4.890268	-2.275382	1.244874

27	1	1.873611	-3.101008	0.567691
28	1	2.641469	-2.713466	2.108630
29	1	-0.855965	-2.057209	2.641927
30	1	2.056320	-0.093801	2.556258
31	1	1.319293	-1.238029	3.694358
32	1	5.293494	0.855949	-1.689503
33	1	6.366180	-0.838879	-0.163830
34	1	-5.122254	2.575122	-1.049388
35	1	-5.595495	-1.478555	0.342030
36	1	-2.698740	-1.701188	1.764440
37	1	-3.558434	-2.801576	0.673330
38	1	-0.095332	-3.605752	0.124516
39	1	-2.608364	-2.086703	-1.805836
40	1	-2.249414	-3.737883	-1.281992
41	8	1.353614	1.203244	-2.032876
42	8	3.307366	2.187924	-2.506965
43	8	-1.183923	2.364693	-0.500951
44	8	-2.832640	3.652701	-1.309369
45	8	1.389972	1.821835	0.938217
46	1	0.862665	2.491485	1.416999
47	1	2.220310	2.253853	0.660452
48	6	-0.587334	-2.665163	-2.172481
49	6	0.024639	0.336882	2.950311
50	8	-0.493050	-3.453952	-3.107246
51	8	0.058138	1.061942	3.948228
52	8	-0.564304	3.280127	2.214914
53	1	-1.044926	2.897724	1.464919
54	1	-0.612363	2.599277	2.907424
55	8	3.708715	3.140241	0.124355
56	1	3.779775	3.016783	-0.837058
57	1	4.515160	2.760697	0.485816
58	59	-0.088586	0.122024	-0.315472
59	1	-1.475387	-4.000296	1.139540
60	1	0.316039	-3.369405	2.612332

```
E(RM062X) = -1856.259007 (Hartree)
Zero-point correction=                    0.458140 (Hartree/Particle)
Thermal correction to Energy=             0.493549
Thermal correction to Enthalpy=           0.494493
Thermal correction to Gibbs Free Energy=  0.393068
Sum of electronic and Zero-point Energies=     -1855.800868
Sum of electronic and Thermal Energies=        -1855.765459
Sum of electronic and Thermal Enthalpies=      -1855.764515
Sum of electronic and Thermal Free Energies=   -1855.865940
```

Table S12: Optimized Cartesian coordinates (Å) of the $[Pr(OCTAPA)_{S,R}(H_2O)]^{-}\cdot 2H_2O$ system obtained with DFT calculations (0 Imaginary Frequencies).

Center Number	Atomic Number	Coordinates (Angstroms) X	Y	Z
1	6	-0.743621	-3.086295	0.298350
2	6	-2.057863	-2.450208	-0.163677

3	6	-3.193240	-3.205987	-0.424873
4	1	-3.158751	-4.276520	-0.276618
5	6	-4.330557	-2.550845	-0.886213
6	1	-5.230334	-3.108425	-1.116031
7	6	-4.300582	-1.171021	-1.056760
8	1	-5.166446	-0.631572	-1.418794
9	6	-3.130405	-0.485133	-0.742584
10	6	-3.041503	1.017776	-0.778458
11	1	-3.808859	1.429430	-1.446532
12	1	-3.262678	1.367450	0.235562
13	6	-1.398629	1.333944	-2.572189
14	1	-0.846816	2.209470	-2.919852
15	6	-0.577035	0.079326	-2.888252
16	1	-1.123426	-0.824976	-2.618457
17	6	1.440008	-1.199448	-2.382862
18	1	1.766896	-1.311650	-3.424705
19	1	0.763959	-2.025223	-2.143616
20	6	2.633188	-1.279695	-1.450222
21	6	3.820499	-1.920332	-1.790675
22	1	3.934822	-2.373383	-2.767437
23	6	4.850439	-1.957564	-0.856140
24	1	5.785250	-2.448017	-1.099015
25	6	4.674010	-1.357458	0.385005
26	1	5.444439	-1.357101	1.143743
27	6	3.457932	-0.740095	0.646696
28	6	3.157229	-0.081198	1.996872
29	6	-1.656861	2.949233	-0.737979
30	1	-2.573725	3.477898	-1.026910
31	1	-0.820030	3.439525	-1.235915
32	6	-1.426564	3.163824	0.768945
33	6	1.553371	1.209552	-2.595451
34	1	1.357624	1.475751	-3.639263
35	1	2.605587	0.924148	-2.524117
36	6	1.391721	2.447981	-1.700601
37	7	-2.041099	-1.123249	-0.313388
38	7	-1.705572	1.527501	-1.131804
39	7	0.715411	0.066640	-2.175129
40	7	2.480975	-0.703283	-0.257204
41	8	-1.084395	2.132487	1.453333
42	8	-1.563886	4.300426	1.201775
43	8	1.951665	0.330867	2.136787
44	8	4.057655	-0.010320	2.819987
45	8	0.232561	-2.277080	0.363711
46	8	-0.746051	-4.288627	0.541307
47	8	1.208739	2.206917	-0.464687
48	8	1.462469	3.558790	-2.225780
49	8	-1.022398	-0.589473	2.607959
50	1	-0.814594	0.059453	3.306622
51	1	-1.988585	-0.714839	2.584133
52	8	-3.741891	-1.063985	2.368046
53	8	0.115785	1.557520	3.875692
54	1	-0.309806	2.055666	3.154104
55	1	0.958361	1.258809	3.493192
56	1	-4.385210	-0.362043	2.233025

57	1	-4.001624	-1.765974	1.761634
58	59	0.131724	0.213904	0.477708
59	1	-0.413756	0.054844	-3.975364
60	1	-2.323269	1.295340	-3.160101

E(RM062X) = -1856.252311 (Hartree)
Zero-point correction= 0.457529 (Hartree/Particle)
Thermal correction to Energy= 0.493441
Thermal correction to Enthalpy= 0.494385
Thermal correction to Gibbs Free Energy= 0.391068
Sum of electronic and Zero-point Energies= -1855.794781
Sum of electronic and Thermal Energies= -1855.758870
Sum of electronic and Thermal Enthalpies= -1855.757926
Sum of electronic and Thermal Free Energies= -1855.861243

Table S13: Optimized Cartesian coordinates (Å) of the [Gd(*CHX*OCTAPA)$_{S,S}$(H_2O)]$^-$·$2H_2O$ system obtained with DFT calculations (0 Imaginary Frequencies).

Center Number	Atomic Number	Coordinates (Angstroms) X	Y	Z
1	7	-1.956762	1.975458	-0.182735
2	7	2.315375	-1.011736	-0.522078
3	7	0.076890	-1.915016	0.902734
4	7	-2.135324	-0.748915	-0.750480
5	8	-0.676610	0.431247	1.956426
6	8	0.095586	-0.693095	-2.270165
7	6	-4.185427	3.599806	-0.383764
8	6	3.484174	-3.047248	-0.146127
9	6	2.320699	-2.279192	-0.121654
10	6	0.994877	-2.879047	0.271786
11	6	-1.329958	-2.435347	0.938642
12	6	0.516728	-1.614990	2.277613
13	6	4.634349	-1.153745	-1.058428
14	6	3.435259	-0.459236	-0.990817
15	6	3.312932	0.995752	-1.440863
16	6	4.655188	-2.473697	-0.622276
17	6	-2.916645	4.159307	-0.292613
18	6	-1.830062	3.305449	-0.180413
19	6	-0.414214	3.823477	0.046170
20	6	-4.319056	2.218378	-0.338258
21	6	-3.172195	1.434218	-0.230626
22	6	-3.249993	-0.061964	-0.080566
23	6	-2.071517	-2.201016	-0.387328
24	6	-2.275477	-0.620419	-2.211439
25	1	-5.061166	4.230483	-0.476912
26	1	3.463217	-4.073012	0.199902
27	1	0.529864	-3.241261	-0.649891
28	1	1.179464	-3.747586	0.913181
29	1	-1.840406	-1.847962	1.708508
30	1	1.594104	-1.434835	2.271888
31	1	0.317363	-2.436094	2.974007
32	1	5.513708	-0.655751	-1.443914

33	1	5.572293	-3.049327	-0.652051
34	1	-2.749072	5.227578	-0.296281
35	1	-5.292868	1.747660	-0.385431
36	1	-3.196428	-0.280448	0.990504
37	1	-4.224511	-0.398590	-0.449578
38	1	-1.491703	-2.679879	-1.184666
39	1	-2.512390	0.421006	-2.451161
40	1	-3.083691	-1.239917	-2.613079
41	8	2.138936	1.447237	-1.487106
42	8	4.363683	1.604282	-1.689534
43	8	0.418435	2.911858	0.366321
44	8	-0.206961	5.027377	-0.046189
45	8	2.058908	0.886691	1.490663
46	6	-3.467310	-2.856627	-0.360461
47	1	-4.086985	-2.355431	0.388348
48	1	-3.955148	-2.714325	-1.327540
49	6	-1.386368	-3.919574	1.353456
50	1	-0.829763	-4.521736	0.630063
51	1	-0.894535	-4.046890	2.320576
52	6	-2.804774	-4.478735	1.395475
53	6	-3.432340	-4.334898	0.013355
54	1	-2.774203	-5.525019	1.707493
55	1	-3.409083	-3.937159	2.131827
56	1	-2.845741	-4.902235	-0.717890
57	1	-4.447378	-4.738020	-0.002196
58	1	1.791936	1.552968	2.153107
59	1	3.012646	1.020769	1.331038
60	6	-0.962854	-0.957332	-2.932161
61	6	-0.114228	-0.324395	2.813177
62	8	-1.019773	-1.415425	-4.069100
63	8	0.016213	-0.068939	4.013350
64	8	0.739926	2.598743	3.236228
65	1	0.276813	2.710600	2.392390
66	1	0.306270	1.830571	3.645128
67	8	4.780948	1.388531	1.102911
68	1	4.919730	1.571192	0.158625
69	1	5.338685	0.629074	1.295484
70	64	0.258948	0.550053	-0.242426

E(RM062X) = -2015.308368 (Hartree)
Zero-point correction= 0.553528 (Hartree/Particle)
Thermal correction to Energy= 0.592053
Thermal correction to Enthalpy= 0.592997
Thermal correction to Gibbs Free Energy= 0.485502
Sum of electronic and Zero-point Energies= -2014.754840
Sum of electronic and Thermal Energies= -2014.716315
Sum of electronic and Thermal Enthalpies= -2014.715371
Sum of electronic and Thermal Free Energies= -2014.822866

Table S14: Optimized Cartesian coordinates (Å) of the [Gd(*CHX*OCTAPA)$_{S,R}$(H_2O)]$^{-}$·2H_2O system obtained with DFT calculations (0 Imaginary Frequencies).

Center Atomic Coordinates (Angstroms)

Number	Number	X	Y	Z
1	6	0.041640	-1.479066	-2.874532
2	6	-1.374458	-1.595256	-2.309051
3	6	-2.449875	-2.057393	-3.056765
4	1	-2.286822	-2.371617	-4.078570
5	6	-3.706069	-2.081100	-2.458808
6	1	-4.571670	-2.420172	-3.014718
7	6	-3.844064	-1.661651	-1.138953
8	1	-4.808124	-1.666494	-0.645952
9	6	-2.708919	-1.238577	-0.454435
10	6	-2.702830	-0.871419	1.002725
11	1	-3.718835	-0.704179	1.370847
12	1	-2.307877	-1.743359	1.533551
13	6	-2.245822	1.592360	0.809326
14	1	-1.887013	2.322095	1.542744
15	6	-3.774199	1.731021	0.764761
16	1	-4.196037	0.991303	0.076107
17	1	-4.176455	1.518329	1.759381
18	6	-4.215392	3.110641	0.284952
19	1	-5.305990	3.168343	0.290338
20	1	-3.845547	3.884106	0.967651
21	6	-3.664009	3.355089	-1.115197
22	1	-3.988553	4.323773	-1.501323
23	1	-4.053818	2.589501	-1.796210
24	6	-2.137800	3.302734	-1.092598
25	1	-1.778241	4.138581	-0.486199
26	1	-1.750258	3.452016	-2.102596
27	6	-1.594623	1.968647	-0.545953
28	1	-1.844635	1.196579	-1.279500
29	6	0.564987	2.200267	-1.718959
30	1	0.433695	3.233152	-2.059699
31	1	0.126070	1.532525	-2.466427
32	6	2.047514	1.900943	-1.615290
33	6	3.012691	2.587785	-2.346259
34	1	2.722965	3.387400	-3.016418
35	6	4.348132	2.230260	-2.190386
36	1	5.117152	2.752400	-2.746584
37	6	4.688134	1.205511	-1.314473
38	1	5.710821	0.891031	-1.157376
39	6	3.664072	0.570049	-0.625935
40	6	3.918213	-0.596286	0.329230
41	6	-1.621638	0.246655	2.788089
42	1	-2.581372	0.129304	3.306754
43	1	-1.182339	1.188133	3.116549
44	6	-0.673290	-0.858957	3.287464
45	6	0.396024	2.917215	0.586573
46	1	-0.273735	3.767144	0.715993
47	1	1.350317	3.324857	0.244455
48	6	0.675847	2.305480	1.965017
49	7	-1.513091	-1.204955	-1.041575
50	7	-1.792603	0.251130	1.320428
51	7	-0.102756	1.952901	-0.423794
52	7	2.391067	0.926566	-0.775681

53	8	-0.026139	-1.513516	2.391106
54	8	-0.599103	-1.033729	4.496143
55	8	2.856866	-1.162587	0.771574
56	8	5.073399	-0.903923	0.582878
57	8	0.862761	-0.906270	-2.093812
58	8	0.248481	-1.918773	-4.000710
59	8	1.063220	1.095274	1.969192
60	8	0.524671	3.026118	2.951963
61	8	0.654614	-2.988017	0.031983
62	1	1.116377	-3.372712	0.801793
63	1	-0.187559	-3.465580	-0.078791
64	8	-1.758266	-4.289248	-0.394332
65	8	2.071526	-3.278673	2.377924
66	1	1.329781	-2.731591	2.697568
67	1	2.644657	-2.617728	1.951306
68	1	-2.428665	-4.361006	0.291532
69	1	-2.240270	-4.056519	-1.195249
70	64	0.599091	-0.523158	0.296389

```
E(RM062X) = -2015.308706 (Hartree)
Zero-point correction=                          0.553037 (Hartree/Particle)
Thermal correction to Energy=                   0.591795
Thermal correction to Enthalpy=                 0.592739
Thermal correction to Gibbs Free Energy=        0.484922
Sum of electronic and Zero-point Energies=         -2014.755669
Sum of electronic and Thermal Energies=            -2014.716911
Sum of electronic and Thermal Enthalpies=          -2014.715967
Sum of electronic and Thermal Free Energies=       -2014.823784
```

Table S15: Optimized Cartesian coordinates (Å) of the $[Gd(OCTAPA)_{S,S}(H_2O)]^{-}\cdot 2H_2O$ system obtained with DFT calculations (0 Imaginary Frequencies).

Center Number	Atomic Number	Coordinates (Angstroms) X	Y	Z
1	7	-2.736399	0.268967	-0.255494
2	7	2.491827	-0.423933	-0.296291
3	7	0.893225	-1.619736	1.607511
4	7	-1.564759	-2.195812	-0.040560
5	8	-0.886254	0.317697	1.997131
6	8	0.360188	-1.653088	-1.813151
7	6	-5.480925	0.559868	-0.450913
8	6	4.459634	-1.481998	0.524069
9	6	3.074400	-1.341031	0.473697
10	6	2.151493	-2.270797	1.220895
11	6	-0.122920	-2.642235	1.935105
12	6	1.100011	-0.755289	2.779701
13	6	4.629297	0.291768	-1.077379
14	6	3.243525	0.368759	-1.061517
15	6	2.482634	1.385661	-1.912154
16	6	5.245591	-0.652832	-0.265221
17	6	-4.639838	1.625188	-0.748062
18	6	-3.271288	1.435519	-0.624443

19	6	-2.279252	2.576482	-0.822962
20	6	-4.926225	-0.640829	-0.026863
21	6	-3.540678	-0.745496	0.064329
22	6	-2.862744	-1.978056	0.603999
23	6	-0.786422	-3.210560	0.694236
24	6	-1.759040	-2.654057	-1.423988
25	1	-6.555575	0.664853	-0.537208
26	1	4.904942	-2.228757	1.169404
27	1	1.918754	-3.098393	0.545587
28	1	2.662637	-2.697134	2.093948
29	1	-0.871100	-2.174451	2.574811
30	1	1.991092	-0.144272	2.619690
31	1	1.243601	-1.341468	3.696297
32	1	5.188958	0.963693	-1.713862
33	1	6.324930	-0.741654	-0.244652
34	1	-5.011630	2.591301	-1.060506
35	1	-5.550051	-1.487294	0.231226
36	1	-2.699025	-1.809751	1.671640
37	1	-3.515868	-2.854428	0.496268
38	1	-0.028332	-3.602904	0.013929
39	1	-2.502120	-2.014438	-1.910130
40	1	-2.129907	-3.685635	-1.461123
41	8	1.231010	1.250254	-1.927514
42	8	3.149646	2.262848	-2.478979
43	8	-1.091935	2.300074	-0.443495
44	8	-2.689484	3.640326	-1.269781
45	8	1.322655	1.743825	1.035738
46	1	0.771213	2.403198	1.500339
47	1	2.121224	2.212845	0.726902
48	6	-0.458257	-2.537437	-2.233538
49	6	-0.060966	0.230200	2.962897
50	8	-0.315221	-3.263381	-3.212003
51	8	-0.090096	0.910444	3.991738
52	8	-0.663610	3.175481	2.331858
53	1	-1.121897	2.776678	1.577551
54	1	-0.700324	2.488931	3.019933
55	8	3.551233	3.192704	0.173763
56	1	3.615899	3.097104	-0.790846
57	1	4.381416	2.846978	0.515019
58	64	-0.080348	0.105406	-0.251317
59	1	0.332547	-3.456124	2.514639
60	1	-1.429598	-4.055652	0.975535

E(RM062X) = -1859.291253 (Hartree)
Zero-point correction= 0.458749 (Hartree/Particle)
Thermal correction to Energy= 0.493965
Thermal correction to Enthalpy= 0.494909
Thermal correction to Gibbs Free Energy= 0.393982
Sum of electronic and Zero-point Energies= -1858.832504
Sum of electronic and Thermal Energies= -1858.797288
Sum of electronic and Thermal Enthalpies= -1858.796343
Sum of electronic and Thermal Free Energies= -1858.897271

Table S16: Optimized Cartesian coordinates (Å) of the $[Gd(OCTAPA)_{S,R}(H_2O)]^{-}\cdot 2H_2O$ system obtained with DFT calculations (0 Imaginary Frequencies).

Center Number	Atomic Number	Coordinates (Angstroms) X	Y	Z
1	7	-2.736399	0.268967	-0.255494
2	7	2.491827	-0.423933	-0.296291
3	7	0.893225	-1.619736	1.607511
4	7	-1.564759	-2.195812	-0.040560
5	8	-0.886254	0.317697	1.997131
6	8	0.360188	-1.653088	-1.813151
7	6	-5.480925	0.559868	-0.450913
8	6	4.459634	-1.481998	0.524069
9	6	3.074400	-1.341031	0.473697
10	6	2.151493	-2.270797	1.220895
11	6	-0.122920	-2.642235	1.935105
12	6	1.100011	-0.755289	2.779701
13	6	4.629297	0.291768	-1.077379
14	6	3.243525	0.368759	-1.061517
15	6	2.482634	1.385661	-1.912154
16	6	5.245591	-0.652832	-0.265221
17	6	-4.639838	1.625188	-0.748062
18	6	-3.271288	1.435519	-0.624443
19	6	-2.279252	2.576482	-0.822962
20	6	-4.926225	-0.640829	-0.026863
21	6	-3.540678	-0.745496	0.064329
22	6	-2.862744	-1.978056	0.603999
23	6	-0.786422	-3.210560	0.694236
24	6	-1.759040	-2.654057	-1.423988
25	1	-6.555575	0.664853	-0.537208
26	1	4.904942	-2.228757	1.169404
27	1	1.918754	-3.098393	0.545587
28	1	2.662637	-2.697134	2.093948
29	1	-0.871100	-2.174451	2.574811
30	1	1.991092	-0.144272	2.619690
31	1	1.243601	-1.341468	3.696297
32	1	5.188958	0.963693	-1.713862
33	1	6.324930	-0.741654	-0.244652
34	1	-5.011630	2.591301	-1.060506
35	1	-5.550051	-1.487294	0.231226
36	1	-2.699025	-1.809751	1.671640
37	1	-3.515868	-2.854428	0.496268
38	1	-0.028332	-3.602904	0.013929
39	1	-2.502120	-2.014438	-1.910130
40	1	-2.129907	-3.685635	-1.461123
41	8	1.231010	1.250254	-1.927514
42	8	3.149646	2.262848	-2.478979
43	8	-1.091935	2.300074	-0.443495
44	8	-2.689484	3.640326	-1.269781
45	8	1.322655	1.743825	1.035738
46	1	0.771213	2.403198	1.500339
47	1	2.121224	2.212845	0.726902
48	6	-0.458257	-2.537437	-2.233538

49	6	-0.060966	0.230200	2.962897
50	8	-0.315221	-3.263381	-3.212003
51	8	-0.090096	0.910444	3.991738
52	8	-0.663610	3.175481	2.331858
53	1	-1.121897	2.776678	1.577551
54	1	-0.700324	2.488931	3.019933
55	8	3.551233	3.192704	0.173763
56	1	3.615899	3.097104	-0.790846
57	1	4.381416	2.846978	0.515019
58	64	-0.080348	0.105406	-0.251317
59	1	0.332547	-3.456124	2.514639
60	1	-1.429598	-4.055652	0.975535

```
E(RM062X) = -1859.291253 (Hartree)
Zero-point correction=                          0.458749 (Hartree/Particle)
Thermal correction to Energy=                   0.493965
Thermal correction to Enthalpy=                 0.494909
Thermal correction to Gibbs Free Energy=        0.393982
Sum of electronic and Zero-point Energies=         -1858.832504
Sum of electronic and Thermal Energies=            -1858.797288
Sum of electronic and Thermal Enthalpies=          -1858.796343
Sum of electronic and Thermal Free Energies=       -1858.897271
```

Table S17: Optimized Cartesian coordinates (Å) of the [Yb(*CHX*OCTAPA)$_{S,S}$(H_2O)]$^{-}$·2H_2O system obtained with DFT calculations (0 Imaginary Frequencies).

Center Number	Atomic Number	Coordinates (Angstroms) X	Y	Z
1	7	-1.602959	2.169144	-0.231606
2	7	2.097687	-1.288375	-0.514650
3	7	-0.215541	-1.933091	0.884449
4	7	-2.196056	-0.463786	-0.782970
5	8	-0.657121	0.471811	1.943968
6	8	0.064898	-0.419919	-2.232139
7	6	-3.543760	4.131436	-0.455388
8	6	3.011121	-3.467939	-0.248978
9	6	1.956624	-2.560160	-0.157167
10	6	0.577459	-3.002817	0.255637
11	6	-1.678246	-2.266056	0.885028
12	6	0.224930	-1.721873	2.275872
13	6	4.359775	-1.704057	-1.146574
14	6	3.263420	-0.866671	-1.009443
15	6	3.312735	0.606763	-1.403156
16	6	4.228519	-3.030759	-0.751173
17	6	-2.202566	4.480172	-0.349480
18	6	-1.269892	3.463825	-0.216750
19	6	0.192218	3.743705	0.099822
20	6	-3.896400	2.790085	-0.397473
21	6	-2.890804	1.832894	-0.279090
22	6	-3.205758	0.370592	-0.120149
23	6	-2.346974	-1.913198	-0.450750
24	6	-2.293347	-0.289578	-2.241925

25	1	-4.306199	4.893694	-0.560268
26	1	2.869703	-4.494591	0.064833
27	1	0.066393	-3.319214	-0.658328
28	1	0.663635	-3.882407	0.902558
29	1	-2.125492	-1.627542	1.653603
30	1	1.316025	-1.687097	2.300262
31	1	-0.103712	-2.519955	2.948866
32	1	5.280368	-1.305707	-1.550890
33	1	5.063509	-3.715758	-0.833599
34	1	-1.865900	5.507852	-0.344737
35	1	-4.932685	2.479814	-0.442500
36	1	-3.193364	0.150522	0.951841
37	1	-4.216545	0.185460	-0.498570
38	1	-1.820334	-2.454561	-1.246191
39	1	-2.479440	0.765154	-2.463387
40	1	-3.113475	-0.864607	-2.681942
41	8	2.215277	1.216501	-1.329676
42	8	4.415467	1.076583	-1.719091
43	8	0.820417	2.710153	0.504228
44	8	0.612833	4.890173	0.008740
45	8	2.094418	0.497521	1.556876
46	6	-3.820147	-2.367684	-0.462477
47	1	-4.377541	-1.796582	0.285652
48	1	-4.265384	-2.142803	-1.434197
49	6	-1.941172	-3.734717	1.274174
50	1	-1.451413	-4.398138	0.556146
51	1	-1.498268	-3.939649	2.251407
52	6	-3.423360	-4.096601	1.273496
53	6	-3.995700	-3.842985	-0.116881
54	1	-3.541747	-5.142996	1.563327
55	1	-3.965133	-3.492245	2.009632
56	1	-3.476936	-4.472030	-0.848950
57	1	-5.055591	-4.103436	-0.159065
58	1	1.918547	1.183012	2.230532
59	1	3.046906	0.556803	1.351092
60	6	-0.977238	-0.652291	-2.930965
61	6	-0.248887	-0.367723	2.813218
62	8	-1.004320	-1.088159	-4.077016
63	8	-0.149721	-0.149286	4.022049
64	8	1.049030	2.367640	3.331799
65	1	0.647481	2.579035	2.474582
66	1	0.466690	1.687743	3.709367
67	8	4.840652	0.782071	1.085770
68	1	4.998668	0.976294	0.147663
69	1	5.338904	-0.021602	1.261635
70	70	0.325150	0.471163	-0.143251

E(RM062X) = -2018.821676 (Hartree)
Zero-point correction= 0.554013 (Hartree/Particle)
Thermal correction to Energy= 0.592475
Thermal correction to Enthalpy= 0.593419
Thermal correction to Gibbs Free Energy= 0.486002
Sum of electronic and Zero-point Energies= -2018.267663
Sum of electronic and Thermal Energies= -2018.229201

```
Sum of electronic and Thermal Enthalpies=          -2018.228257
Sum of electronic and Thermal Free Energies=       -2018.335674
```

Table S18: Optimized Cartesian coordinates (Å) of the [Yb(*CHX*OCTAPA)$_{S,R}$(H_2O)]$^-$·2H_2O system obtained with DFT calculations (0 Imaginary Frequencies).

Center Number	Atomic Number	Coordinates (Angstroms) X	Y	Z
1	6	0.107629	1.404343	2.849925
2	6	-1.307376	1.570588	2.302320
3	6	-2.360218	2.057826	3.065130
4	1	-2.176311	2.356280	4.088127
5	6	-3.621056	2.125142	2.480834
6	1	-4.471133	2.483233	3.048630
7	6	-3.781848	1.725661	1.157682
8	1	-4.748492	1.765950	0.671452
9	6	-2.666048	1.276028	0.458667
10	6	-2.679750	0.926569	-1.000300
11	1	-3.700008	0.780869	-1.365000
12	1	-2.271742	1.796365	-1.523425
13	6	-2.288152	-1.542642	-0.841377
14	1	-1.938066	-2.270508	-1.580725
15	6	-3.819140	-1.648901	-0.815625
16	1	-4.232862	-0.915841	-0.114859
17	1	-4.207912	-1.408030	-1.809184
18	6	-4.289440	-3.030402	-0.369749
19	1	-5.380761	-3.068971	-0.386811
20	1	-3.926354	-3.795029	-1.065909
21	6	-3.757527	-3.316379	1.030609
22	1	-4.102072	-4.288441	1.389977
23	1	-4.143731	-2.560914	1.724860
24	6	-2.230067	-3.287287	1.029129
25	1	-1.873905	-4.116358	0.411390
26	1	-1.858618	-3.461673	2.041182
27	6	-1.664328	-1.949836	0.515975
28	1	-1.917396	-1.188908	1.259865
29	6	0.481969	-2.232762	1.700974
30	1	0.331735	-3.271303	2.015279
31	1	0.053505	-1.575532	2.463317
32	6	1.967091	-1.954881	1.595802
33	6	2.930118	-2.652250	2.319082
34	1	2.636931	-3.449205	2.990957
35	6	4.268024	-2.307498	2.153976
36	1	5.036047	-2.837889	2.703701
37	6	4.610909	-1.282309	1.279560
38	1	5.634972	-0.974118	1.118500
39	6	3.586506	-0.636124	0.601896
40	6	3.828222	0.542751	-0.335344
41	6	-1.587726	-0.178572	-2.783055
42	1	-2.530703	-0.023076	-3.322072
43	1	-1.163157	-1.121675	-3.125553
44	6	-0.601535	0.921029	-3.211126

45	6	0.320727	-2.896638	-0.615992
46	1	-0.379529	-3.711586	-0.795681
47	1	1.243075	-3.360206	-0.258236
48	6	0.672270	-2.257548	-1.963396
49	7	-1.466109	1.194451	1.032492
50	7	-1.790315	-0.208787	-1.321756
51	7	-0.172827	-1.942508	0.408499
52	7	2.312716	-0.983895	0.754543
53	8	0.022156	1.526341	-2.264457
54	8	-0.479975	1.147328	-4.407233
55	8	2.758457	1.122249	-0.738225
56	8	4.977444	0.853286	-0.612167
57	8	0.896198	0.809519	2.052718
58	8	0.345918	1.826152	3.976487
59	8	1.041214	-1.042413	-1.926559
60	8	0.589949	-2.963368	-2.968456
61	8	0.680731	2.872559	0.052781
62	1	1.163702	3.265226	-0.699054
63	1	-0.156890	3.362413	0.139996
64	8	-1.682293	4.287673	0.416558
65	8	2.107767	3.292350	-2.296573
66	1	2.657991	2.608545	-1.875581
67	1	1.349717	2.760038	-2.603233
68	1	-2.337606	4.386507	-0.280495
69	1	-2.188396	4.086170	1.210977
70	70	0.580538	0.474628	-0.264306

E(RM062X) = -2018.828540 (Hartree)
Zero-point correction= 0.553783 (Hartree/Particle)
Thermal correction to Energy= 0.592275
Thermal correction to Enthalpy= 0.593219
Thermal correction to Gibbs Free Energy= 0.486227
Sum of electronic and Zero-point Energies= -2018.274757
Sum of electronic and Thermal Energies= -2018.236265
Sum of electronic and Thermal Enthalpies= -2018.235321
Sum of electronic and Thermal Free Energies= -2018.342312

Table S19: Optimized Cartesian coordinates (Å) of the $[Yb(OCTAPA)_{S,S}(H_2O)]^{-}\cdot 2H_2O$ system obtained with DFT calculations (0 Imaginary Frequencies).

Center Number	Atomic Number	Coordinates (Angstroms) X	Y	Z
1	7	-2.665172	0.235038	-0.295446
2	7	2.444529	-0.405800	-0.299383
3	7	0.895423	-1.692297	1.557790
4	7	-1.512865	-2.200850	-0.144017
5	8	-0.911118	0.193653	1.989508
6	8	0.257444	-1.311069	-1.921491
7	6	-5.407019	0.544879	-0.538984
8	6	4.445819	-1.493041	0.399098
9	6	3.059888	-1.346364	0.413938
10	6	2.174177	-2.301730	1.170731

11	6	-0.101395	-2.753843	1.812336
12	6	1.061148	-0.891947	2.781296
13	6	4.551111	0.325967	-1.153027
14	6	3.169097	0.408696	-1.070742
15	6	2.380644	1.464921	-1.840252
16	6	5.200114	-0.644889	-0.398932
17	6	-4.554762	1.613299	-0.789946
18	6	-3.191208	1.414092	-0.636204
19	6	-2.191144	2.556911	-0.729024
20	6	-4.865755	-0.665765	-0.126784
21	6	-3.482831	-0.779558	-0.012540
22	6	-2.814528	-2.025826	0.501937
23	6	-0.729376	-3.251155	0.525757
24	6	-1.704460	-2.574876	-1.552053
25	1	-6.479054	0.656532	-0.646503
26	1	4.914124	-2.260942	1.002032
27	1	1.972017	-3.144978	0.504998
28	1	2.702034	-2.705018	2.044322
29	1	-0.869132	-2.343279	2.467758
30	1	1.944580	-0.258834	2.677154
31	1	1.191673	-1.527081	3.666219
32	1	5.082561	1.017493	-1.792486
33	1	6.278834	-0.737882	-0.429702
34	1	-4.915265	2.590114	-1.081859
35	1	-5.497320	-1.514435	0.103667
36	1	-2.663131	-1.889718	1.575627
37	1	-3.460739	-2.901675	0.355831
38	1	0.049557	-3.574630	-0.168907
39	1	-2.508318	-1.969063	-1.979564
40	1	-1.988158	-3.628643	-1.657322
41	8	1.129891	1.376271	-1.746910
42	8	3.029103	2.331830	-2.443919
43	8	-1.037074	2.254247	-0.275602
44	8	-2.564463	3.642381	-1.154783
45	8	1.297351	1.634644	1.175989
46	1	0.740951	2.274090	1.662666
47	1	2.056091	2.139068	0.825124
48	6	-0.444613	-2.278850	-2.369509
49	6	-0.124391	0.057341	2.983887
50	8	-0.220265	-2.938262	-3.378111
51	8	-0.212853	0.666270	4.051766
52	8	-0.696978	3.029103	2.504282
53	1	-1.132313	2.673695	1.714833
54	1	-0.770772	2.313158	3.157783
55	8	3.433221	3.193168	0.258107
56	1	3.492863	3.155736	-0.709943
57	1	4.283141	2.870463	0.572395
58	70	-0.059751	0.115510	-0.162484
59	1	0.364179	-3.590387	2.349085
60	1	-1.356417	-4.129600	0.731497

E(RM062X) = -1862.804368 (Hartree)
Zero-point correction= 0.459732 (Hartree/Particle)
Thermal correction to Energy= 0.494664

```
Thermal correction to Enthalpy=                  0.495608
Thermal correction to Gibbs Free Energy=         0.395531
Sum of electronic and Zero-point Energies=          -1862.344636
Sum of electronic and Thermal Energies=             -1862.309704
Sum of electronic and Thermal Enthalpies=           -1862.308760
Sum of electronic and Thermal Free Energies=        -1862.408837
```

Table S20: Optimized Cartesian coordinates (Å) of the $[Yb(OCTAPA)_{S,R}(H_2O)]^{-}\cdot 2H_2O$ system obtained with DFT calculations (0 Imaginary Frequencies).

Center Number	Atomic Number	Coordinates (Angstroms) X	Y	Z
1	6	-0.698005	-2.976056	0.396554
2	6	-2.015048	-2.370722	-0.082032
3	6	-3.151393	-3.135640	-0.303902
4	1	-3.116307	-4.198597	-0.108717
5	6	-4.289509	-2.500470	-0.790856
6	1	-5.190267	-3.066294	-0.995236
7	6	-4.257920	-1.129057	-1.016646
8	1	-5.123993	-0.601309	-1.394968
9	6	-3.085332	-0.433493	-0.736266
10	6	-2.984366	1.062523	-0.826272
11	1	-3.740703	1.463697	-1.512132
12	1	-3.203978	1.448158	0.174416
13	6	-1.356019	1.313544	-2.631346
14	1	-0.765267	2.159977	-2.987227
15	6	-0.593354	0.019629	-2.928824
16	1	-1.184464	-0.857622	-2.663836
17	6	1.381746	-1.309474	-2.361264
18	1	1.733226	-1.433505	-3.393276
19	1	0.691570	-2.124917	-2.129673
20	6	2.546155	-1.371674	-1.393600
21	6	3.729340	-2.057583	-1.646185
22	1	3.869363	-2.576877	-2.586034
23	6	4.722228	-2.054436	-0.670635
24	1	5.654263	-2.578240	-0.844794
25	6	4.510850	-1.376524	0.524595
26	1	5.249277	-1.349605	1.314290
27	6	3.298113	-0.722497	0.695747
28	6	2.919702	-0.000503	1.986021
29	6	-1.526940	2.934923	-0.784423
30	1	-2.435030	3.498724	-1.030548
31	1	-0.691780	3.410294	-1.297900
32	6	-1.249419	3.080156	0.719258
33	6	1.556941	1.084925	-2.596294
34	1	1.351800	1.381236	-3.629203
35	1	2.597426	0.754905	-2.559748
36	6	1.473289	2.307350	-1.675246
37	7	-1.991324	-1.051680	-0.291761
38	7	-1.635290	1.522194	-1.190128
39	7	0.675243	-0.029403	-2.177978
40	7	2.364333	-0.715023	-0.249334

41	8	-0.959068	1.998220	1.348771
42	8	-1.318022	4.197919	1.211994
43	8	1.700728	0.396279	2.030482
44	8	3.762234	0.129193	2.861353
45	8	0.271555	-2.157113	0.395300
46	8	-0.685385	-4.161939	0.708633
47	8	1.208438	2.055123	-0.456912
48	8	1.678562	3.418145	-2.162020
49	8	-0.972564	-0.539723	2.389541
50	1	-0.749698	0.092158	3.099203
51	1	-1.944681	-0.603133	2.366767
52	8	-3.730252	-0.871487	2.335198
53	8	0.029775	1.593055	3.867502
54	1	0.853445	1.267100	3.464292
55	1	-0.392006	2.035315	3.107048
56	1	-4.359612	-0.150620	2.239831
57	1	-4.071287	-1.579599	1.778383
58	70	0.118458	0.194031	0.352389
59	1	-0.403128	-0.022012	-4.010389
60	1	-2.285319	1.315047	-3.212438

```
E(RM062X) = -1862.808784 (Hartree)
Zero-point correction=                           0.459481 (Hartree/Particle)
Thermal correction to Energy=                    0.494509
Thermal correction to Enthalpy=                  0.495453
Thermal correction to Gibbs Free Energy=         0.395416
Sum of electronic and Zero-point Energies=          -1862.349304
Sum of electronic and Thermal Energies=             -1862.314275
Sum of electronic and Thermal Enthalpies=           -1862.313331
Sum of electronic and Thermal Free Energies=        -1862.413368
```

Table S21: Optimized Cartesian coordinates (Å) of the [Lu(*CHX*OCTAPA)$_{S,S}$(H_2O)]$^-$·2H_2O system obtained with DFT calculations (0 Imaginary Frequencies).

Center Number	Atomic Number	Coordinates (Angstroms) X	Y	Z
1	7	-1.531660	2.198751	-0.227652
2	7	2.045719	-1.341102	-0.531321
3	7	-0.275904	-1.937328	0.872141
4	7	-2.209189	-0.413036	-0.792716
5	8	-0.647321	0.470868	1.950114
6	8	0.055349	-0.284406	-2.239800
7	6	-3.413727	4.216732	-0.479576
8	6	2.900398	-3.548917	-0.297243
9	6	1.872826	-2.612830	-0.186585
10	6	0.486918	-3.023869	0.235638
11	6	-1.746569	-2.231319	0.870218
12	6	0.168894	-1.750414	2.265711
13	6	4.291043	-1.813865	-1.184951
14	6	3.220722	-0.946806	-1.028716
15	6	3.316306	0.531379	-1.395917
16	6	4.125381	-3.141159	-0.804891

17	6	-2.064680	4.526685	-0.356525
18	6	-1.163700	3.484316	-0.209227
19	6	0.298597	3.723171	0.136091
20	6	-3.804800	2.886322	-0.419875
21	6	-2.829041	1.900696	-0.286595
22	6	-3.190285	0.449733	-0.124649
23	6	-2.402160	-1.857073	-0.465634
24	6	-2.304628	-0.230196	-2.250791
25	1	-4.152613	5.000009	-0.597089
26	1	2.731960	-4.574407	0.006860
27	1	-0.037929	-3.328254	-0.674496
28	1	0.556944	-3.906528	0.880431
29	1	-2.177805	-1.582742	1.639523
30	1	1.260505	-1.744897	2.291384
31	1	-0.181743	-2.545243	2.931208
32	1	5.219857	-1.436462	-1.590473
33	1	4.939853	-3.848563	-0.902079
34	1	-1.698113	5.544036	-0.348339
35	1	-4.848856	2.605255	-0.475024
36	1	-3.180109	0.229333	0.947224
37	1	-4.208174	0.297336	-0.498748
38	1	-1.889580	-2.410619	-1.262109
39	1	-2.517505	0.820410	-2.467149
40	1	-3.107189	-0.822847	-2.698951
41	8	2.244221	1.179302	-1.289382
42	8	4.430411	0.967321	-1.720599
43	8	0.889700	2.668868	0.542650
44	8	0.751908	4.858215	0.065017
45	8	2.101761	0.412924	1.569231
46	6	-3.887672	-2.269718	-0.480732
47	1	-4.429179	-1.684853	0.268537
48	1	-4.325231	-2.028698	-1.452101
49	6	-2.050594	-3.693290	1.255023
50	1	-1.577519	-4.368264	0.536514
51	1	-1.616398	-3.913113	2.232851
52	6	-3.542370	-4.013744	1.250492
53	6	-4.105379	-3.740378	-0.139884
54	1	-3.690439	-5.057181	1.537261
55	1	-4.068370	-3.396480	1.987376
56	1	-3.603502	-4.381741	-0.873049
57	1	-5.172108	-3.970840	-0.184218
58	1	1.947237	1.094389	2.252583
59	1	3.054051	0.451268	1.356854
60	6	-0.978442	-0.549126	-2.939669
61	6	-0.270055	-0.389033	2.813995
62	8	-0.989435	-0.978568	-4.088193
63	8	-0.174132	-0.185290	4.025358
64	8	1.117942	2.293013	3.363146
65	1	0.723789	2.530959	2.509398
66	1	0.509584	1.632557	3.733958
67	8	4.851665	0.620771	1.086286
68	1	5.017563	0.825931	0.152049
69	1	5.323459	-0.201544	1.248649
70	71	0.343329	0.464688	-0.119674

```
----------------------------------------------------------------------
E(RM062X) = -2019.403572 (Hartree)
Zero-point correction=                           0.554078 (Hartree/Particle)
Thermal correction to Energy=                    0.592577
Thermal correction to Enthalpy=                  0.593521
Thermal correction to Gibbs Free Energy=         0.485566
Sum of electronic and Zero-point Energies=          -2018.849494
Sum of electronic and Thermal Energies=             -2018.810995
Sum of electronic and Thermal Enthalpies=           -2018.810051
Sum of electronic and Thermal Free Energies=        -2018.918007
```

Table S22: Optimized Cartesian coordinates (Å) of the [Lu(*CHX*OCTAPA)$_{S,R}$(H_2O)]$^-$·2H_2O system obtained with DFT calculations (0 Imaginary Frequencies).

Center Number	Atomic Number	Coordinates (Angstroms) X	Y	Z
1	7	-1.531660	2.198751	-0.227652
2	7	2.045719	-1.341102	-0.531321
3	7	-0.275904	-1.937328	0.872141
4	7	-2.209189	-0.413036	-0.792716
5	8	-0.647321	0.470868	1.950114
6	8	0.055349	-0.284406	-2.239800
7	6	-3.413727	4.216732	-0.479576
8	6	2.900398	-3.548917	-0.297243
9	6	1.872826	-2.612830	-0.186585
10	6	0.486918	-3.023869	0.235638
11	6	-1.746569	-2.231319	0.870218
12	6	0.168894	-1.750414	2.265711
13	6	4.291043	-1.813865	-1.184951
14	6	3.220722	-0.946806	-1.028716
15	6	3.316306	0.531379	-1.395917
16	6	4.125381	-3.141159	-0.804891
17	6	-2.064680	4.526685	-0.356525
18	6	-1.163700	3.484316	-0.209227
19	6	0.298597	3.723171	0.136091
20	6	-3.804800	2.886322	-0.419875
21	6	-2.829041	1.900696	-0.286595
22	6	-3.190285	0.449733	-0.124649
23	6	-2.402160	-1.857073	-0.465634
24	6	-2.304628	-0.230196	-2.250791
25	1	-4.152613	5.000009	-0.597089
26	1	2.731960	-4.574407	0.006860
27	1	-0.037929	-3.328254	-0.674496
28	1	0.556944	-3.906528	0.880431
29	1	-2.177805	-1.582742	1.639523
30	1	1.260505	-1.744897	2.291384
31	1	-0.181743	-2.545243	2.931208
32	1	5.219857	-1.436462	-1.590473
33	1	4.939853	-3.848563	-0.902079
34	1	-1.698113	5.544036	-0.348339
35	1	-4.848856	2.605255	-0.475024
36	1	-3.180109	0.229333	0.947224

37	1	-4.208174	0.297336	-0.498748
38	1	-1.889580	-2.410619	-1.262109
39	1	-2.517505	0.820410	-2.467149
40	1	-3.107189	-0.822847	-2.698951
41	8	2.244221	1.179302	-1.289382
42	8	4.430411	0.967321	-1.720599
43	8	0.889700	2.668868	0.542650
44	8	0.751908	4.858215	0.065017
45	8	2.101761	0.412924	1.569231
46	6	-3.887672	-2.269718	-0.480732
47	1	-4.429179	-1.684853	0.268537
48	1	-4.325231	-2.028698	-1.452101
49	6	-2.050594	-3.693290	1.255023
50	1	-1.577519	-4.368264	0.536514
51	1	-1.616398	-3.913113	2.232851
52	6	-3.542370	-4.013744	1.250492
53	6	-4.105379	-3.740378	-0.139884
54	1	-3.690439	-5.057181	1.537261
55	1	-4.068370	-3.396480	1.987376
56	1	-3.603502	-4.381741	-0.873049
57	1	-5.172108	-3.970840	-0.184218
58	1	1.947237	1.094389	2.252583
59	1	3.054051	0.451268	1.356854
60	6	-0.978442	-0.549126	-2.939669
61	6	-0.270055	-0.389033	2.813995
62	8	-0.989435	-0.978568	-4.088193
63	8	-0.174132	-0.185290	4.025358
64	8	1.117942	2.293013	3.363146
65	1	0.723789	2.530959	2.509398
66	1	0.509584	1.632557	3.733958
67	8	4.851665	0.620771	1.086286
68	1	5.017563	0.825931	0.152049
69	1	5.323459	-0.201544	1.248649
70	71	0.343329	0.464688	-0.119674

```
E(RM062X) = -2019.403572 (Hartree)
Zero-point correction=                           0.554078 (Hartree/Particle)
Thermal correction to Energy=                    0.592577
Thermal correction to Enthalpy=                  0.593521
Thermal correction to Gibbs Free Energy=         0.485566
Sum of electronic and Zero-point Energies=          -2018.849494
Sum of electronic and Thermal Energies=             -2018.810995
Sum of electronic and Thermal Enthalpies=           -2018.810051
Sum of electronic and Thermal Free Energies=        -2018.918007
```

Table S23: Optimized Cartesian coordinates (Å) of the $[Lu(OCTAPA)_{S,S}(H_2O)]^{-}\cdot 2H_2O$ system obtained with DFT calculations (0 Imaginary Frequencies).

Center Number	Atomic Number	Coordinates (Angstroms) X	Y	Z
1	7	-2.643450	0.235296	-0.298775
2	7	2.426325	-0.411710	-0.321297

3	7	0.901089	-1.717624	1.540787
4	7	-1.503785	-2.207011	-0.166562
5	8	-0.904899	0.159068	2.007598
6	8	0.177561	-1.192257	-1.969938
7	6	-5.381641	0.547204	-0.578612
8	6	4.437413	-1.507145	0.336483
9	6	3.052081	-1.358729	0.374536
10	6	2.179553	-2.319321	1.139991
11	6	-0.093125	-2.784898	1.782290
12	6	1.069688	-0.937892	2.777408
13	6	4.520825	0.325803	-1.199978
14	6	3.140813	0.411013	-1.094471
15	6	2.345203	1.484685	-1.832104
16	6	5.180195	-0.653800	-0.466308
17	6	-4.526154	1.618018	-0.807283
18	6	-3.165281	1.418108	-0.633406
19	6	-2.165698	2.563782	-0.683431
20	6	-4.846408	-0.665800	-0.165334
21	6	-3.465356	-0.781004	-0.032558
22	6	-2.803517	-2.030635	0.482639
23	6	-0.720660	-3.265395	0.489580
24	6	-1.701104	-2.566763	-1.577939
25	1	-6.451916	0.659296	-0.701937
26	1	4.913743	-2.280533	0.925960
27	1	1.976360	-3.164154	0.476706
28	1	2.718661	-2.719782	2.007800
29	1	-0.861445	-2.385384	2.443882
30	1	1.951757	-0.301744	2.681328
31	1	1.203594	-1.588331	3.650505
32	1	5.043116	1.023763	-1.839921
33	1	6.258102	-0.748264	-0.515328
34	1	-4.882739	2.597618	-1.094694
35	1	-5.481214	-1.515584	0.051571
36	1	-2.650617	-1.895140	1.556150
37	1	-3.453162	-2.903698	0.335563
38	1	0.058287	-3.578814	-0.210182
39	1	-2.536601	-1.990279	-1.984427
40	1	-1.941352	-3.629555	-1.696121
41	8	1.096876	1.412271	-1.704109
42	8	2.988743	2.350520	-2.442700
43	8	-1.025206	2.255426	-0.199528
44	8	-2.528441	3.654712	-1.103380
45	8	1.310316	1.602285	1.211830
46	1	0.759723	2.236108	1.712678
47	1	2.059071	2.113632	0.849458
48	6	-0.472268	-2.199075	-2.411172
49	6	-0.117424	0.004898	2.999389
50	8	-0.224342	-2.838374	-3.426929
51	8	-0.207066	0.592695	4.078500
52	8	-0.667614	2.988910	2.570935
53	1	-1.107336	2.660759	1.771759
54	1	-0.755353	2.260189	3.207981
55	8	3.419840	3.181407	0.269220
56	1	3.468898	3.160036	-0.699778

57	1	4.278126	2.867184	0.568958
58	71	-0.058399	0.119061	-0.133774
59	1	0.375073	-3.627495	2.307195
60	1	-1.346632	-4.147427	0.683477

```
E(RMN062X) = -1863.386128 (Hartree)
Zero-point correction=                          0.459671 (Hartree/Particle)
Thermal correction to Energy=                   0.494687
Thermal correction to Enthalpy=                 0.495632
Thermal correction to Gibbs Free Energy=        0.394936
Sum of electronic and Zero-point Energies=         -1862.926457
Sum of electronic and Thermal Energies=            -1862.891441
Sum of electronic and Thermal Enthalpies=          -1862.890497
Sum of electronic and Thermal Free Energies=       -1862.991193
```

Table S24: Optimized Cartesian coordinates (Å) of the $[Lu(OCTAPA)_{S,R}(H_2O)]^{-}\cdot 2H_2O$ system obtained with DFT calculations (0 Imaginary Frequencies).

Center Number	Atomic Number	Coordinates (Angstroms) X	Y	Z
1	6	-0.694558	-2.968998	0.404425
2	6	-2.011624	-2.365861	-0.075611
3	6	-3.147973	-3.131687	-0.293726
4	1	-3.112562	-4.193812	-0.094129
5	6	-4.286338	-2.498680	-0.782854
6	1	-5.187160	-3.065322	-0.984662
7	6	-4.254653	-1.128236	-1.013924
8	1	-5.120785	-0.601712	-1.393813
9	6	-3.081952	-0.431570	-0.736754
10	6	-2.981352	1.063824	-0.832354
11	1	-3.736966	1.462444	-1.520474
12	1	-3.202047	1.453063	0.166652
13	6	-1.353516	1.310074	-2.637326
14	1	-0.760100	2.154222	-2.994191
15	6	-0.594520	0.013416	-2.931525
16	1	-1.188715	-0.861720	-2.666481
17	6	1.377490	-1.317929	-2.358430
18	1	1.729824	-1.443143	-3.389974
19	1	0.686890	-2.132828	-2.126328
20	6	2.540784	-1.377578	-1.389469
21	6	3.723872	-2.065464	-1.636851
22	1	3.864965	-2.589636	-2.573833
23	6	4.715281	-2.057997	-0.659754
24	1	5.647261	-2.583244	-0.829812
25	6	4.502343	-1.374227	0.531869
26	1	5.239381	-1.344185	1.322772
27	6	3.289534	-0.718986	0.697710
28	6	2.906654	0.007447	1.983606
29	6	-1.520141	2.932853	-0.790696
30	1	-2.427288	3.498904	-1.035175
31	1	-0.684513	3.407154	-1.304339
32	6	-1.241612	3.073561	0.712986

33	6	1.556160	1.075382	-2.595545
34	1	1.350598	1.373188	-3.627862
35	1	2.595869	0.742861	-2.561005
36	6	1.477022	2.296978	-1.673414
37	7	-1.987349	-1.047599	-0.290721
38	7	-1.631814	1.520785	-1.196648
39	7	0.671769	-0.037116	-2.177067
40	7	2.357558	-0.715153	-0.248900
41	8	-0.955645	1.988602	1.339291
42	8	-1.306452	4.189825	1.209634
43	8	1.686218	0.399954	2.023467
44	8	3.746538	0.144193	2.860452
45	8	0.274632	-2.149763	0.397947
46	8	-0.681071	-4.153380	0.722047
47	8	1.206898	2.045103	-0.456219
48	8	1.690504	3.407045	-2.158254
49	8	-0.969716	-0.537446	2.378397
50	1	-0.744966	0.094750	3.087021
51	1	-1.942203	-0.594618	2.355537
52	8	-3.729262	-0.855995	2.333982
53	8	0.024619	1.599809	3.862888
54	1	-0.397540	2.037160	3.099792
55	1	0.847808	1.272785	3.459564
56	1	-4.357264	-0.133784	2.239881
57	1	-4.075132	-1.564829	1.781106
58	71	0.117804	0.192134	0.346071
59	1	-0.401478	-0.030665	-4.012444
60	1	-2.282604	1.313513	-3.218730

```
---------------------------------------------------------------------
E(RM062X) = -1863.392114 (Hartree)
Zero-point correction=                          0.459589 (Hartree/Particle)
Thermal correction to Energy=                   0.494582
Thermal correction to Enthalpy=                 0.495527
Thermal correction to Gibbs Free Energy=        0.395573
Sum of electronic and Zero-point Energies=         -1862.932525
Sum of electronic and Thermal Energies=            -1862.897531
Sum of electronic and Thermal Enthalpies=          -1862.896587
Sum of electronic and Thermal Free Energies=       -1862.996541
```

References

(1) Tircsó, G.; Regueiro-Figueroa, M.; Nagy, V.; Garda, Z.; Garai, T.; Kálmán, F. K.; Esteban-Gómez, D.; Tóth, É.; Platas-Iglesias, C. Approaching the Kinetic Inertness of Macrocyclic Gadolinium(III)-Based MRI Contrast Agents with Highly Rigid Open-Chain Derivatives. *Chem. - Eur. J.* **2016**, *22* (3), 896–901. https://doi.org/10.1002/chem.201503836.

(2) Ramogida, C. F.; Cawthray, J. F.; Boros, E.; Ferreira, C. L.; Patrick, B. O.; Adam, M. J.; Orvig, C. H $_2$ *CHX* Dedpa and H $_4$ *CHX* Octapa—Chiral Acyclic Chelating Ligands for $^{67/68}$ Ga and ^{111} In Radiopharmaceuticals. *Inorg. Chem.* **2015**, *54* (4), 2017–2031. https://doi.org/10.1021/ic502942a.
